# Lipid‑disrupting effects of an organochlorine mixture in Sertoli TM4 cells reveal testicular obesogen mechanisms

**DOI:** 10.1007/s00204-026-04446-4

**Published:** 2026-05-20

**Authors:** Ishita Virmani, Eliška Sychrová, Eliška Řehůřková, Darshak Gadara, Zdeněk Spáčil, Iva Sovadinová

**Affiliations:** RECETOX, Faculty of Science, Masaryk University, Kotlarska 2, Brno, Czech Republic

**Keywords:** Endocrine-disrupting chemicals, Lipidomics, Male reproductive toxicity, Mixture assessment, Obesogens, Organochlorines, Sertoli cells

## Abstract

Male reproductive health is declining globally, reflected by reduced sperm counts, impaired semen quality, increasing infertility, and a rising incidence of testicular cancer. Endocrine‑disrupting chemicals include metabolic disruptors, within which obesogens represent a specific subgroup that dysregulate lipid metabolism and cellular energy homeostasis and are increasingly implicated in these adverse trends. Although obesogens act across multiple tissues, their lipid‑metabolism‑related effects on testicular cells remain comparatively understudied. We investigated male reprotoxicity of an environmentally and human‑relevant organochlorine mixture (OC‑MIX), consisting of 20 persistent contaminants, including polychlorinated biphenyls (PCBs), chlordane, and dichlorodiphenyltrichloroethane/dichlorodiphenyldichloroethane (DDT/DDE), originally identified in ringed seal blubber and representative of Arctic food‑chain exposures. Although OC‑MIX has shown male reproductive toxicity in animal models, its cellular mechanisms in Sertoli cells remain insufficiently defined. Using immature murine Sertoli TM4 cells, we demonstrated that OC‑MIX (5–50 µg/mL) disrupted lipid homeostasis and induced oxidative stress, culminating in lipotoxicity, processes known to compromise Sertoli cell support of spermatogenesis. These responses differed from those elicited by a fatty acid mixture or the lipotoxicant amiodarone and did not appear to involve androgen receptor, aryl hydrocarbon receptor, or peroxisome proliferator‑activated α signaling, suggesting distinct lipid‑centered mechanisms. Comparative profiling in immature murine Leydig TM3 cells revealed cell‑type‑specific responses, likely reflecting intrinsic differences in lipid composition. Notably, OC‑MIX selectively altered phospholipid and lysophospholipid species in Sertoli TM4 cells, indicating interference with membrane remodeling and lipid‑dependent signaling. Overall, these findings support the hypothesis that persistent organochlorines act as testicular obesogens, contributing to male reproductive dysfunction through selective, lipid‑disrupting mechanisms.

**Supplementary Information:**

The online version contains supplementary material available at 10.1007/s00204-026-04446-4.

## Introduction

Male reproductive health is declining worldwide, reflected by reduced sperm count and quality, hormonal disturbances, anatomical defects, and rising testicular cancer incidence (Le Cornet et al. 2014; Babakhanzadeh et al. 2020). Infertility affects 13–18% of couples globally, with male and female factors contributing equally (Barratt et al. 2017). Environmental exposures, particularly endocrine‑disrupting chemicals (EDCs) and their mixtures, are considered major contributors to this trend (Sharma et al. 2020).

Metabolic disruptors, a subset of EDCs, interfere with metabolic signaling, mitochondrial function, and cellular stress responses (Amon et al. 2024). Obesogens within this group promote lipid accumulation through peroxisome proliferator-activated g/α (PPARg/α), estrogen (ER), androgen (AR), and aryl hydrocarbon (AhR) receptors, disrupting energy balance and potentially impairing Leydig and Sertoli cell function (La Merrill et al. 2024). Oxidative stress, implicated in up to 80% of male infertility cases, is further exacerbated by obesity and metabolic disruption (Tremellen 2008; Wright et al. 2014).

Organochlorines, historically used in agriculture and vector control, remain a concern due to their persistence, bioaccumulation, and metabolic toxicity despite decades‑long bans (Jayaraj et al. 2016). Human body burdens typically reach tens to hundreds of ng/g lipid in adipose tissue and sub‑µg/L serum concentrations, with higher levels in historically exposed or occupationally exposed populations (Aneck-Hahn et al. 2007; Arrebola et al. 2012; Richardson et al. 2014; Artacho-Cordón et al. 2015; Achour et al. 2017, 2023; Bornman et al. 2018; Bandow et al. 2020). Continued environmental inputs and slow degradation sustain chronic, low‑dose global exposure. Arctic Inuit communities exhibit particularly elevated burdens and reduced life expectancy (Donaldson et al. 2010).

Prenatal and lifelong exposures to DDT/DDE (dichlorodiphenyltrichloroethane/dichlorodiphenyldichloroethylene) and related organochlorines have been associated with obesity, disrupted lipid metabolism, and impaired reproductive function (Cano-Sancho et al. 2017; La Merrill et al. 2020; Merrill et al. 2024). Epidemiological evidence further links these compounds to reduced male fertility, testicular dysgenesis syndrome, and increased testicular cancer risk (Cook et al. 2011; Rato and Sousa 2021; Xu et al. 2023). Yet the mechanisms underlying organochlorine‑induced testicular toxicity, particularly their metabolic and lipid‑disrupting actions under realistic exposure scenarios, remain poorly understood.

To address this gap, we investigated OC‑MIX (Table S1, Fig. S1), a 20‑component organochlorine mixture composed primarily of chlordane, DDT/DDE, polychlorinated biphenyls (PCBs; individual congeners, Aroclor 1254/1260), toxaphene, and lindane. OC-MIX reflects contamination of the Northern Canadian food chain and was originally identified in ringed seal blubber (Campagna et al. 2001). It impairs male reproductive function in vivo, causing delayed puberty, reduced testis weight, disrupted spermatogenesis, decreased testosterone, and multigenerational effects on sperm quality and fertility (Anas et al. 2005; Maurice et al. 2018; Lessard et al. 2019). In Leydig cell models (MA‑10, MLTC‑1, TM3), OC‑MIX induces oxidative stress, disrupts steroidogenesis, and alters mitochondrial and lipid metabolism; spermatozoa exhibit reduced motility and viability (Campagna et al. 2002, 2009; Enangue Njembele et al. 2014, 2022; Virmani et al. 2026). Despite this evidence, OC‑MIX has not been studied in Sertoli cells.

Building on our previous work in immature murine Leydig TM3 cells (Virmani et al. 2026), we now characterized OC‑MIX effects in immature murine TM4 Sertoli cells and directly compared responses across both testicular cell types. Using an in vitro test battery (Fig. S2), we evaluated cell viability, oxidative stress, and lipid homeostasis, with emphasis on lipidomics, and compared OC‑MIX activity with lipotoxic stressors (amiodarone, fatty acids) and receptor ligands to identify potential cell‑type‑specific vulnerabilities during sensitive developmental windows.

## Materials and methods

### Chemicals and experimental design

Table S1 summarizes the composition, vendor information, catalog numbers, and purity of all OC‑MIX components, and Table S2 lists all reagents and materials. Stock solutions were prepared in dimethyl sulfoxide (DMSO; final exposure concentration primarily 0.1%), with complete dissolution and vortexing before each experiment to ensure consistent dosing. Based on previous studies (Enangue Njembele et al. 2014, 2022; Virmani et al. 2026), cumulative test concentrations ranged from 2.5 to 50 µg/mL (~ 7–143 µM) (Table S1, Fig. S1), and each experiment included untreated, solvent, and positive controls (Fig. S2). Experimental concentration ranges were further optimized based on preliminary testing to ensure endpoint sensitivity, with cytotoxicity assessed before and alongside each assay, because persistent OC‑MIX components accumulate in lipid compartments or media (Jayaraj et al. 2016). Substantial total concentration shifts were not expected.

### Cell culture and maintenance

Murine Sertoli TM4 cells (American Type Culture Collection ATCC CRL‑1715) were used due to the absence of human Sertoli cell lines. Cells were maintained at passages 15–30 following established protocols (Kubincová et al. 2019; Yawer et al. 2022), as detailed in Text S1.

### Cell viability assays

Real-time cell analysis (RTCA) was conducted using the xCELLigence RTCA SP instrument (ACEA Biosciences, San Diego, CA, USA). Cell viability was also assessed using alamarBlue (dehydrogenase activity), CFDA‑AM (esterase activity and membrane integrity), neutral red (lysosomal function), and MTT (metabolic activity), following established procedures (Yawer et al. 2022) described in Text S2.

### ROS assay

Reactive oxygen species (ROS) were quantified using CellRoX™ Green reagent (Thermo Fisher Scientific, Cat. No. C10444) as described in Virmani et al. (2026) (details in Text S3). Menadione (100 µM) served as a positive control, showing time‑dependent ROS induction and reduced cell numbers (Fig. S3).

### Lipid accumulation assay

Neutral lipid accumulation was monitored using BODIPY™ 493/50,326 (Invitrogen, Cat. No. 1987239), as described previously (Virmani et al. 2026). Images were acquired and analyzed using the BioTek Cytation 5 Cell Imaging Multimode Reader (details in Text S4). Positive controls—oleic/palmitic acid (OAPA, 2:1 in DMSO) and amiodarone—robustly induced lipid accumulation in Sertoli TM4 cells (Figs. S4, S5).

#### LC-MS/MS lipidomics

Lipidomics followed the 48‑h lipid accumulation study design, except that samples were prepared in transparent 96‑well plates. Sample preparation and LC‑MS/MS analysis were performed as described in Virmani et al. (2026) (Text S5). Four wells per group were analyzed, and each experiment was repeated three times using independently prepared cells (12 samples/condition). Lipid concentrations were calculated using internal standards and not normalized to protein content, as selected concentrations did not affect total protein (Fig. S6). Data were expressed as log₂ fold change relative to the solvent control (DMSO 0.1%).

Lipid nomenclature followed LIPID MAPS guidelines (Liebisch et al. 2020) and included five lipid categories and 21 subclasses (Table S3): (1) Fatty acyls: acylcarnitines (CAR); (2) glycerolipids: diacylglycerols (DG), triacylglycerols (TG); (3) Sterol lipids: free cholesterol (FC), cholesteryl esters (CE); (4) Glycerophospholipids: phosphatidylcholines (PC), phosphatidylethanolamines (PE), ether-linked phosphatidylethanolamines (PE-O), alkenylphosphatidylcholines (PC-P), alkylphosphatidylcholines (PC-O), lysophosphatidylethanolamines (LPE), lysophosphatidylcholines (LPC), alkyl ether-linked lysophosphatidylcholines (LPC-O), phosphatidylglycerols (PG), phosphatidylinositols (PI), phosphatidylserines (PS); and (5) Sphingolipids: dihydroceramides (dhCer), ceramides (Cer), hexosylceramides (HexCer), dihexosylceramides (Hex2Cer), and sphingomyelins (SM).

Pathway enrichment was conducted using BioPAN (Bioinformatics Methodology for Pathway Analysis; Gaud et al. (2021). Fifteen species (HexCer, Hex2Cer, CAR), were not included in the BioPAN database. A total of 143 species (41 glycerolipids, 100 glycerophospholipids, 2 sphingolipids) were mapped to at least one reaction. Default settings were applied, with significance at *p* < 0.05.

### Protein estimation

Protein quantification mirrored the lipidomic experimental design. Total protein was measured using a BCA (bicinchoninic acid) assay kit (ThermoFisher Scientific, Cat. No. 23225), with absorbance recorded at 562 nm using a Biotek Synergy microplate reader.

### RT-PCR

Expression of selected receptors (mouse AhR, AR, PPARα) and reference genes (EEF2—eukaryotic elongation factor 2, RPL38—ribosomal protein L38) was assessed using the protocol primer sequences reported in Virmani et al. (2026) (Text S6).

### Data and statistical analysis

All experiments were conducted independently at least three times with ≥ 3 replicates. Blank values were subtracted when applicable. Data were normalized to negative controls and expressed as a fraction of control (FOC). Statistical analysis was performed using Sigmaplot 12.3 (Systat Software, San Jose, CA, USA), and graphs were generated with GraphPad Prism 10 (GraphPad Software, La Jolla, CA, USA). Normality was assessed by Shapiro–Wilk. For normally distributed data with equal variance, one‑way ANOVA with Dunnett’s or Dunn’s post hoc tests or two‑tailed t‑tests were applied. Otherwise, Mann–Whitney U or Kruskal–Wallis ANOVA with appropriate post hoc tests were used. Statistical significance was set at *p* ≤ 0.05.

Dose‑response curves were fitted using a four‑parameter nonlinear sigmoidal model in GraphPad Prism 10. EC_50_ (the concentration inducing a 50% response) and ET_50_ (the time for a 50% response) values were derived, and geometric means with 95% CIs (confidence intervals) were calculated across independent experiments.

## Results

### Cell viability and oxidative stress

**Fig. 1:**
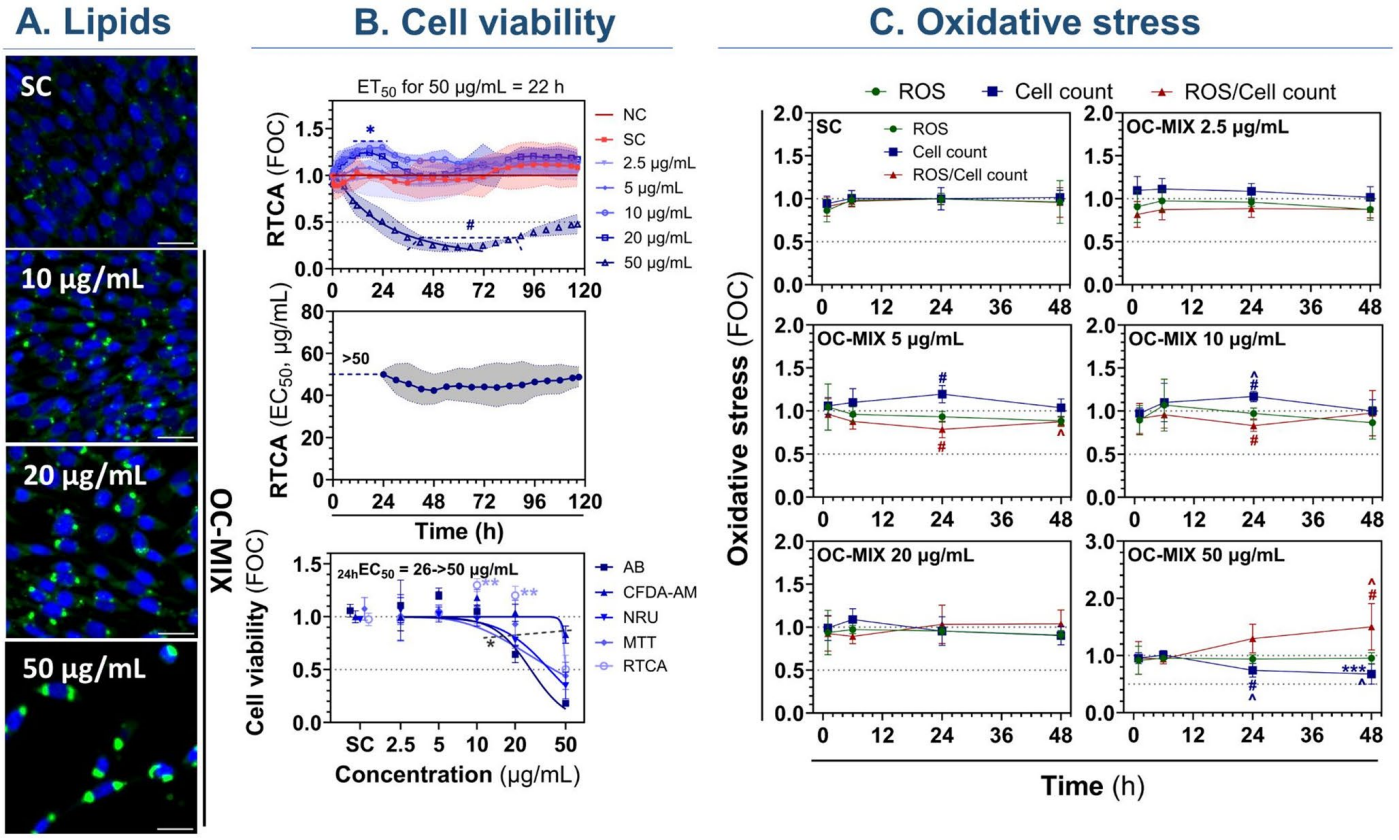
Direct impact of the organochlorine mixture OC‑MIX on Sertoli TM4 cells. **A** Neutral lipid accumulation after 48 h, visualized with BODIPY™ 493/503 (lipid droplets, green) and DAPI (nuclei, blue). Quantification for 24 h and 48 h, including lower concentrations, is provided in Fig. S9. **B** Cell viability monitored by real‑time cell analysis (RTCA) over 117 h, with EC_50_ values shown as mean ± CI. Viability at 1, 24, and 117 h was also assessed using AB, CFDA‑AM, NR, and MTT assays (Fig. S7). **C** Oxidative stress was evaluated by ROS production using CellROX from 1 to 48 h. Data represent mean ± SD from ≥ 3 independent experiments (*n* = 3–6). Significant differences vs. the solvent control (SC, 0.1% DMSO) are indicated by * or ^#^, and differences vs. the 1‑h exposure are marked with ^. Statistical tests: one-way ANOVA with Dunnett’s test (*p ≤ 0.05, **p ≤ 0.01, ***p ≤ 0.001) or Kruskal-Wallis with Dunnett’s/Dunn’s tests (#, ^ *p* ≤ 0.05)

Sertoli TM4 cell viability was monitored for 117 h after OC‑MIX exposure. Within the first hour, OC-MIX caused small but significant reductions in esterase activity and membrane integrity (14% at 20 µg/mL; 37% at 50 µg/mL) (Figs. 1B, S7). Between 3 and 36 h, impedance showed modest increases (1.1–1.3× at 10–20 µg/mL) without consistent metabolic or membrane changes. By 24 h, viability decreased by up to 40% at 20 µg/mL, while 50 µg/mL reduced viability below 50% in all assays except CFDA‑AM (ET_50_ = 22 h). Prolonged exposure did not enhance toxicity; instead, partial recovery occurred, and EC_50_ values stabilized near 50 µg/mL by 117 h.

ROS measurements (1–48 h; Fig. 1C) showed that 5 and 10 µg/mL OC-MIX modestly increased cell numbers and reduced ROS per cell at 24 h. No significant effects occurred at 2.5–20 µg/mL. At 50 µg/mL, total ROS remained unchanged, but reduced cell numbers resulted in elevated ROS per cell at 48 h. Menadione (100 µM) induced early ROS production (1 h) followed by combined cytotoxic and oxidative effects over 6–48 h (Fig. S3).

### Lipid accumulation

Neutral lipid accumulation was measured by BODIPY 493/503 staining. The fatty acid mixture (OAPA) induced strong lipid accumulation at 24 and 48 h (up to 7-fold droplet area; 9-fold total signal per cell) (Figs. S4, S5). Amiodarone increased lipid accumulation only at ≥ 5 µM, producing a modest ~ 2‑fold rise at 24 h and a ~ 6‑fold increase at 48 h at 10 µM, accompanied by ~ 20–40% reductions in cell number (Figs. S4, S8).

OC-MIX significantly increased neutral lipid accumulation at 20 and 50 µg/mL after 24 h (6–13‑fold droplet area; 8–29‑fold signal per cell), with even stronger effects at 48 h (up to 38‑fold and 93‑fold, respectively), accompanied by 30–90% reductions in cell number (Figs. 1A, S9). Moderate increases (2–3‑fold) occurred at lower, non‑cytotoxic concentrations (2.5–10 µg/mL), with significant effects at 10 µg/mL at 48 h.

To explore receptor involvement, we tested ligands for AR, AhR, and PPARα, given the known antiandrogenic and dioxin‑like actions of OC‑MIX (Enangue Njembele et al. 2022; Virmani et al. 2026) and the roles of PPARα in lipid metabolism and testicular pathophysiology (Bougarne et al. 2018; Monrose et al. 2021). All three receptors were expressed in Sertoli TM4 cells (mRNA,  Fig. S10; AR protein also detected (Yawer et al. 2022). However, none of the ligands induced lipid accumulation (Fig. S11), indicating these pathways do not mediate OC‑MIX‑induced lipid dysregulation.

### Lipidomics

**Fig. 2:**
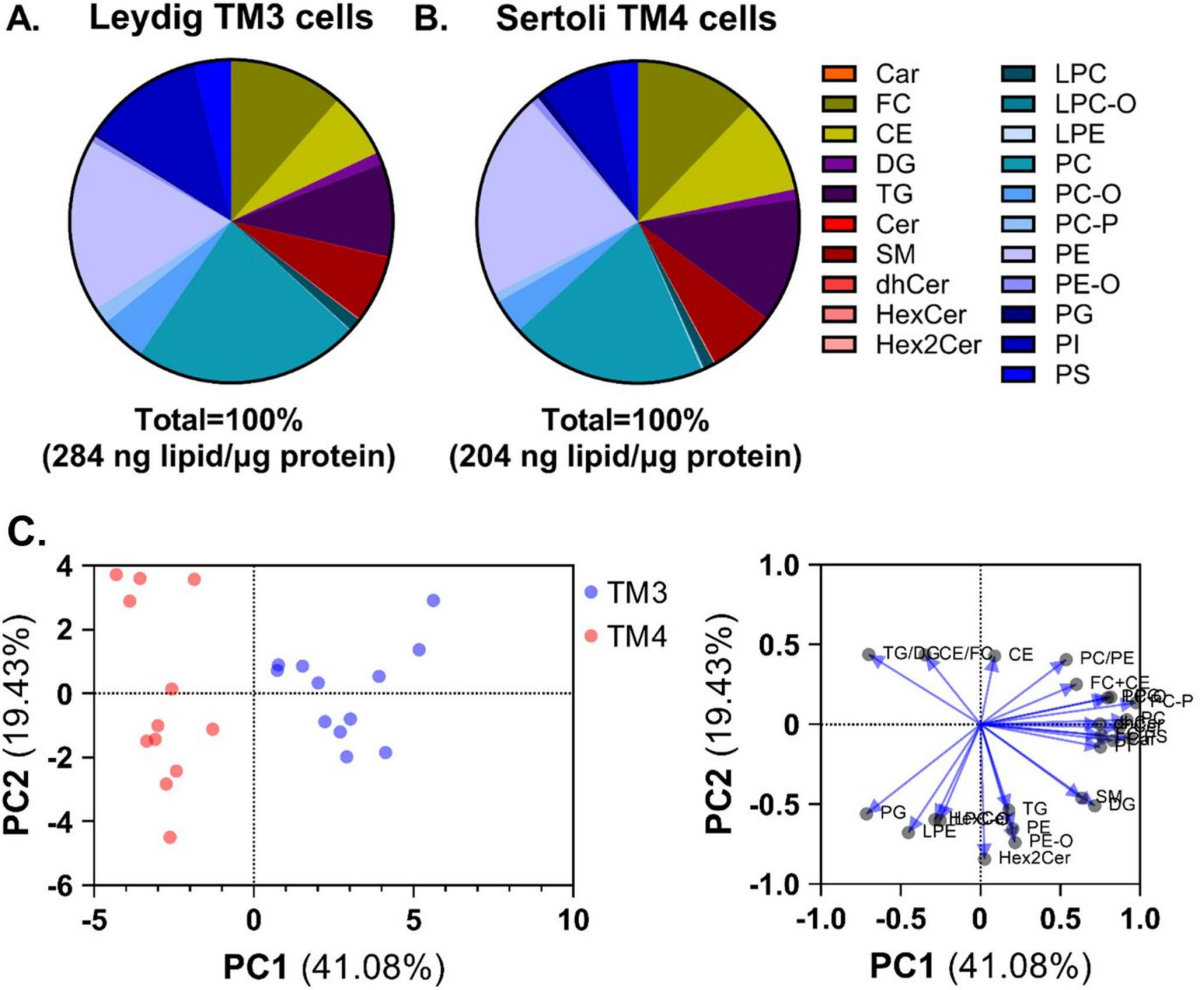
Comparative lipid profiling of untreated Leydig TM3 and Sertoli TM4 cells at the subclass level. **A**, **B** Relative lipid concentrations normalized to protein (ng/µg) and subclass contributions (% of total lipids) in Leydig TM3 (**A**) and Sertoli TM4 (**B**) cells. Full quantitative data and statistics are provided in Table S4. **C** Principal component analysis (PCA) score and loading plots illustrating lipid profile differences between the two cell types

We compared the lipid profiles of Sertoli TM4 cells (this study) with Leydig TM3 cells (Virmani et al. 2026) (Fig. 2, Table S4). Total lipid content (normalized to protein) was 1.4× higher in Leydig TM3 cells. Both cell types shared the same overall lipid category ranking (glycerophospholipids > sterol lipids > glycerolipids > sphingolipids > fatty acyls), but Leydig TM3 cells contained more sterol lipids, sphingolipids, and glycerophospholipids, whereas glycerolipids were similar. Fatty acyls CAR were least abundant but 12× higher in Leydig TM3 cells.

Principal component analysis clearly separated the two cell types. Leydig TM3 cells had 1.6–2.5× higher levels of PC, LPC, PC-O, PC-P, PI, and PS, while LPC-O, LPE, PE, and PE-O were comparable. FC was 1.3× higher; CE levels were unchanged; DG levels were 1.6× higher; and TG levels were similar. SM and dhCer were also elevated (1.4× and 1.8×). PG was the only subclass more abundant in Sertoli TM4 cells (1.7×).

Lipid subclass rankings were broadly similar, with PC and PE most abundant. Leydig TM3 cells showed a higher PC: PE ratio, whereas Sertoli TM4 cells showed a lower PC: PE ratio due to lower PC levels. TG ranked higher in Sertoli TM4 cells (3rd vs. 5th), while DG ranked higher in Leydig TM3 cells, yielding a higher TG: DG ratio in Sertoli TM4 cells. PI ranked higher in Leydig TM3 cells (3rd vs. 6th); FC ranked fourth in both cell types; CE ranked higher in Sertoli TM4 cells (5th vs. 7th). CER was more abundant in Leydig TM3 cells (15th vs. 20th), while HexCer was slightly higher in Sertoli TM4 cells (17th vs. 20th).

Of 275 detected species (Table S5), 245 were shared, and 15 were unique to each cell type. Sertoli TM4‑specific species included CE (3), TG (3), HexCer (1), PC (2), PC‑O (1), PC‑P (1), PE‑O (1), PG (1), and PI (2). TM3‑specific species included Cer (4), SM (2), PC (5), PE (1), and PI (3). Quantitative analysis (Fig. S12) confirmed these differences: among neutral lipids, 23 DG/TG species were similar, 22 were higher, and 19 were lower in Sertoli TM4 cells. For PI, five species (PI32:1, 34:1, 36:1, 38:6, 40:5) were 2–18× higher, whereas three (PI36:4, 38:4, 38:5) were 3–4× lower in Sertoli TM4 cells compared to Leydig TM3 cells.

**Fig. 3:**
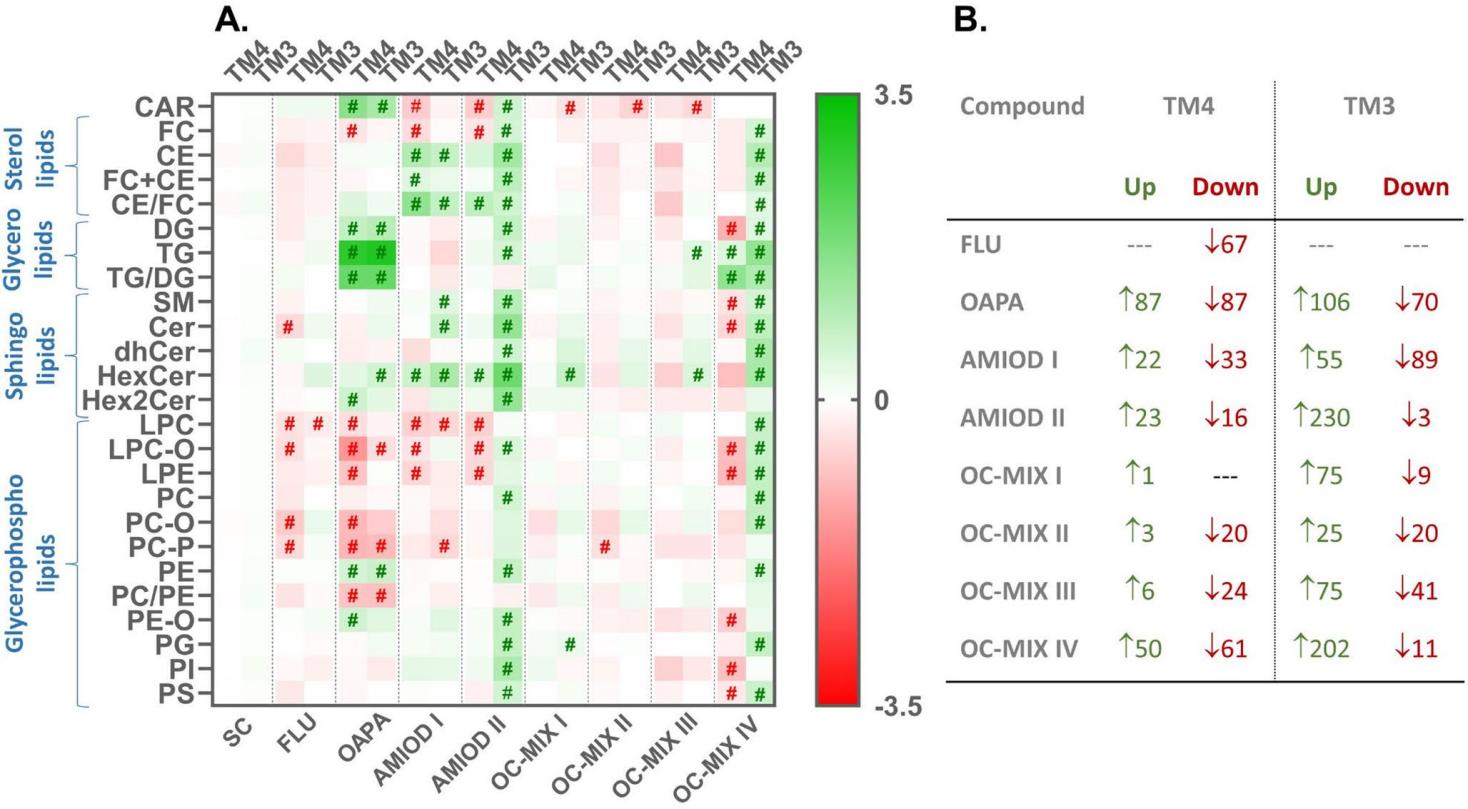
Lipid profile alterations in Sertoli TM4 and Leydig TM3 cells after 48-h exposure to the organochlorine mixture OC-MIX and control compounds. **A** Mean Log₂(fold-change) in lipid subclasses relative to solvent control (SC, 0.1% DMSO) following treatment with flutamide (FLU, 1 µM), oleic/palmitic acid mixture (2:1 ratio; OAPA, 100 µM), amiodarone (AMIOD; I: 2.5 µM, II: 5 µM), and OC-MIX (I: 2.5 µg/mL, II: 5 µg/mL, III: 10 µg/mL, IV: 20 µg/mL). Color scale: green (upregulation), red (downregulation), white (no change). Significant differences (#, *p* < 0.05) were determined by Kruskal–Wallis ANOVA with Dunnett’s/Dunn’s post hoc tests. **B** Number of significantly altered lipid species per treatment. Data represent three independent experiments performed in quadruplicate (*n* = 12). Lipid data for Leydig TM3 cells were previously published (Virmani et al. 2026)

We conducted LC-MS/MS lipidomics to evaluate the effects of OC-MIX (2.5–20 µg/mL) and positive controls —fatty acid mixture (100 µM), amiodarone (2.5, 5 µM), and the AR antagonist flutamide (1 µM)—on the Sertoli TM4 lipidome at non‑ or minimally cytotoxic concentrations. Flutamide induced only modest lipid changes across a limited number of subclasses and species (Figs. 3, S13A). In contrast, OC-MIX and the positive controls produced broader and more pronounced lipid disruptions (Figs. 3, S13B–D, S14).

OC-MIX had little effect on total CAR but reduced 3 long‑chain unsaturated CAR species to ~ 70% of control at 5–20 µg/mL (Figs. 3, S15). Amiodarone decreased total CAR to 60% and reduced 5 individual CAR species—overlapping with those affected by OC‑MIX—by up to 75%. The fatty acid mixture produced the opposite effect, increasing total CAR and the same species up to 3-fold.

Consistent with the BODIPY data, OC-MIX and positive controls increased neutral lipids in a subclass-dependent manner (Figs. 3, S16). At 20 µg/mL, OC-MIX elevated total TG by 1.4-7-fold and upregulated 38 of 56 TG species (up to 3-fold), while reducing total DG to 50% and lowering 8 of 11 DG species up to 60%, resulting in a 3- to 4-fold rise in the TG: DG ratio. The fatty mixture induced stronger TG accumulation (7‑fold total; 47 species up to 21‑fold) and moderately increased DG (2‑fold total), yielding a similar TG: DG shift. Amiodarone caused only minor reductions in select TG and DG species at 2.5 µM.

Unlike TG and DG, OC-MIX did not markedly affect CE, FC, or the CE: FC ratio (Figs. 3, S16). The fatty acid mixture increased 3 of 9 CE species (up to 1.7-fold) and decreased 1 by 50%. Amiodarone produced stronger CE accumulation (2‑fold at 2.5 µM; 1.4‑fold at 5 µM), elevating 7 CE species by up to 10‑fold. Both positive controls decreased FC by 20–30%, raising the CE: FC ratio—up to 3‑fold for amiodarone and 1.4‑fold for the fatty acid mixture—indicating preferential CE storage.

Alterations in glycerophospholipids were strongly treatment‑specific (Figs. 3, S17, S18). OC-MIX primarily reduced lysophospholipids, lowering total LPC-O, LPE, and PE-O by up to 40% (5 of 9 PE-O species down by 80%). Total LPC, PC, PE, and the PC: PE ratio were unchanged, although individual PE species varied (6 increased up to 4‑fold; 4 decreased up to 37%). OC-MIX increased LPC22:5 (up to 2-fold), slightly reduced 2 LPC species (up to 60%), and decreased PC-O and PC-P (except at the lowest dose). It also reduced total PI and 7 PI species and reduced 4 PS species (up to 40%), while PG remained unchanged.

Amiodarone reduced total lysophospholipids by ~ 30%, decreasing 4 LPC, all LPC-O, and 2 LPE species (25–40%). Class‑level changes in other subclasses were minimal, although PI36:1 (1.5‑fold), PI36:3, PG36:4, and PG32:1 (~ 3‑fold) were upregulated.

The fatty acid mixture had the strongest effect, reducing LPC-O (60%), LPE (40%), and LPC (30%). All LPC-O species dropped to 20%, 2 of 3 LPE species to 50%, and all LPC species (up to 40%). It increased total PE 1.5-fold (lowering the PC: PE ratio by 40%) and modulated individual species (7 PE up to 2.5‑fold; 5 PE down up to 40%; 12 PC species down 80–90%). Total PC‑O and PC‑P decreased ~ 50%, whereas PE‑O increased 1.5‑fold. PI changes included 2 species up (~ 2‑fold) and 4 down, along with reductions in 3 PG and 5 PS species (to ~ 30%).

Sphingolipid changes (Figs. 3, S19) showed that OC‑MIX reduced total CER by 30% (e.g., Cer(d18:2/24:1) down 50%) and total SM by 20% (6 of 24 species down up to 35%). Although the 50% decrease in total HexCer was not significant, OC‑MIX significantly reduced 4 of 6 HexCer and 3 of 5 Hex2Cer species (down up to ~ 50%).

In contrast, amiodarone increased total HexCer 1.5‑fold, elevating 4 species up to 3.5‑fold, and increased dhCer(d18:0/16:0) 1.7‑fold, 2 CER species (up to 1.8‑fold), 2 Hex2Cer (up to 1.5‑fold), and 3 SM species (up to 1.6‑fold).

The fatty acid mixture similarly increased total HexCer (1.3‑fold), 4 HexCer species (up to 3.7‑fold), and 3 Hex2Cer species (up to 2.4‑fold), while reducing others to 30–40%. It lowered dhCer(d18:1/24:0) and Cer(d18:1/24:0) to ~ 30% but increased Cer(d18:2/16:0) and Cer(d18:2/24:1) up to 1.6‑fold. Eleven SM species decreased (to 20%), and 3 increased slightly (~ 1.3‑fold).

### Lipid pathway enrichment analysis

**Fig. 4:**
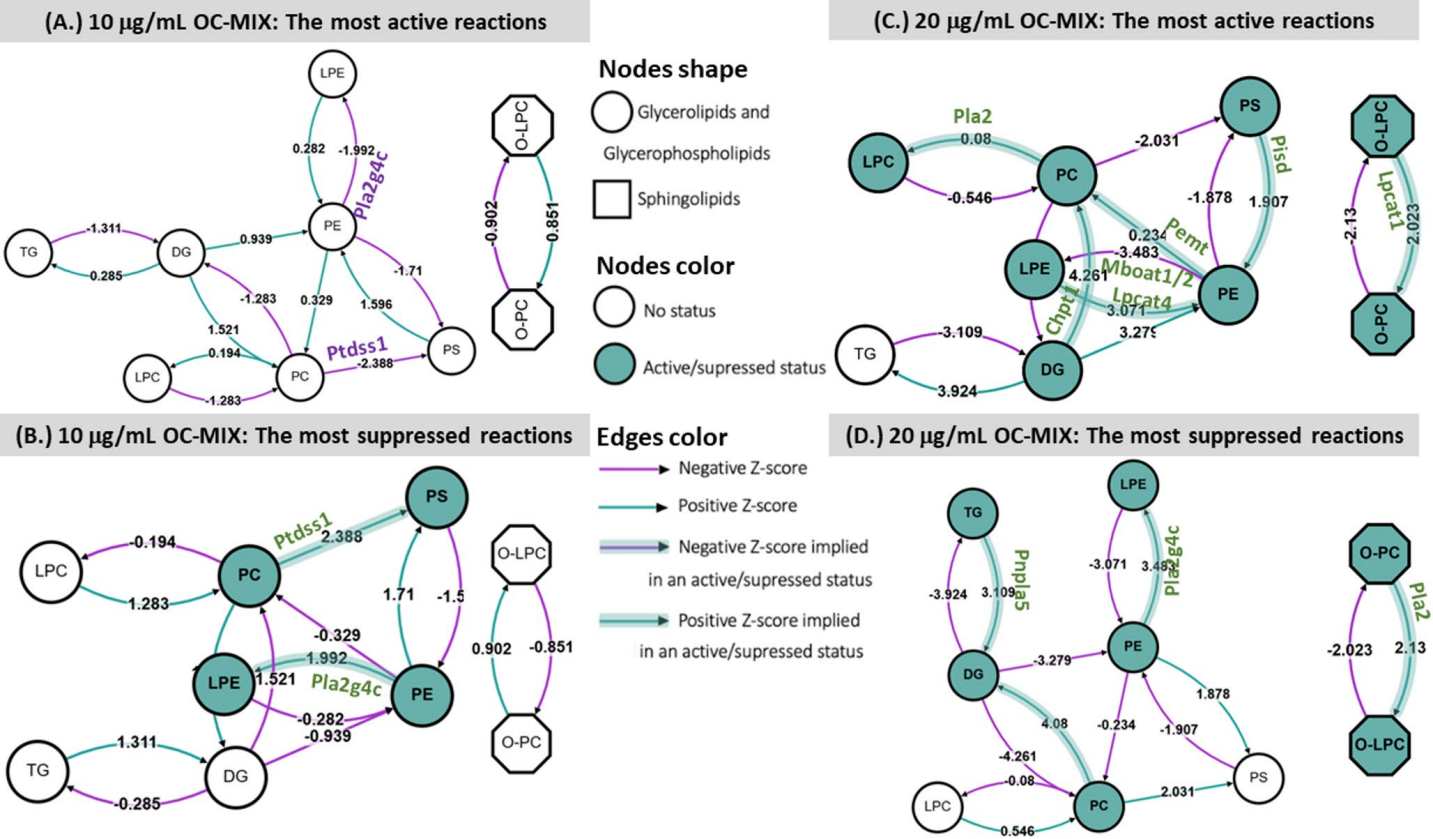
Lipid pathway enrichment in Sertoli TM4 cells after 48-h exposure to the organochlorine mixture OC-MIX (10 and 20 µg/mL). BioPAN analysis identified the most active (**A**, **C**) and suppressed (**B**, **D**) reactions relative to the solvent control (0.1% DMSO) and suggested the enzymes involved. The default BioPAN settings were applied, with a significance threshold of *p* ≤ 0.05. Z-scores indicate the direction and magnitude of lipid pathway changes: positive values reflect activation, while negative values indicate suppression, helping to pinpoint key enzymatic reactions affected by chemical exposure

BioPAN analysis identified dose‑dependent alterations in lipid metabolic pathways induced by OC‑MIX and the positive controls (Figs. 4, S20-S22; Table S6). At 2.5 µg/mL, OC-MIX suppressed PE → PC conversion via Pemt (phosphatidylethanolamine N-methyltransferase). At 5 µg/mL, it activated O‑PC → O‑LPC through Pla2 (phospholipase A2), suppressing the reverse O‑LPC → O‑PC and LPC → PC reactions mediated by Lpcat1/4 (lysophosphatidylcholine acyltransferase 1/4). At 10 µg/mL, no reactions were activated, but PC → PS (Ptdss1, phosphatidylserine synthase 1) and PE → LPE (Pla2g4c) were suppressed, suggesting enhanced PC and PE synthesis. At 20 µg/mL, multiple reactions were activated, including DG → PC (Chpt1, choline phosphotransferase 1), PC → LPC (Pla2), O‑LPC → O‑PC (Lpcat1), LPE → PE (Mboat1/2, membrane-bound O-acyltransferases 1/2, or Lpcat4), PE → PC (Pemt), and PS → PE (Pisd, phosphatidylserine decarboxylase), indicating accelerated PC, PE, and LPC biosynthesis. Suppression of TG → DG (Pnpla5) suggested reduced TG breakdown, favoring TG accumulation.

For amiodarone, 2.5 µM activated LPC → PC (Lpcat1/4) and PC → PS (Ptdss1), while suppressing PS → PE (Pisd) (Fig. S21; Table S5). It also activated LPC‑O → PC‑O (Lpcat1) and suppressed PC → LPC (Pla2). At 5 µM, amiodarone further stimulated PC → PS (Ptdss1), activated PE → LPE (Pla2g4c), and suppressed LPC → PC and O‑LPC → O‑PC (Lpcat1/4), as well as PC → DG and DG → TG (Dgat2, diacylglycerol acyltransferase 2), consistent with inhibition of TG synthesis.

The fatty acid mixture (100 µM) strongly promoted TG biosynthesis by activating PC → DG and DG → TG via Dgat2 (Fig. S22; Table S5). It also activated LPE → PE (Mboat1/2 or Lpcat4), PS → PE (Pisd), and LPC‑O → PC‑O (Lpcat1), while suppressing PE → PC (Pemt), indicating enhanced TG formation accompanied by remodeling of glycerophospholipid pathways.

To assess cell-specific responses, we compared the lipid-disrupting effects of OC-MIX and the positive controls in Sertoli TM4 cells with our previous findings in Leydig TM3 cells (Virmani et al. 2026). OC‑MIX produced distinct, cell‑specific patterns, with clear PCA separation at 20 µg/mL (Fig. S23A). Leydig TM3 cells showed broader lipidomic disruption, affecting more subclasses and species (Fig. 3). TG was the only subclass consistently increased in both cell types. Several lipid subclasses that were downregulated in Sertoli TM4 cells—DG, SM, CER, LPC‑O, LPE, PE‑O, PI, and PS—were unchanged or upregulated in Leydig TM3 cells, and OC‑MIX did not alter FC or CE in Sertoli TM4 cells, whereas both changed markedly in Leydig TM3 cells.

Amiodarone also elicited cell‑specific effects. PCA indicated partial separation at 2.5 µM and clear separation at 5 µM (Fig. S23B, C). Sertoli TM4 cells did not display a dose‑dependent pattern, unlike Leydig TM3 cells (Fig. 3). At 2.5 µM, both cell types shared changes in CE, HexCer, and LPC; however, Sertoli TM4 cells uniquely showed reductions in CAR, FC, LPC‑O, and LPE, while TM3 cells exhibited changes in SM, CER, and PC‑P. At 5 µM, Leydig TM3 cells displayed broad lipid induction, whereas Sertoli TM4 cells exhibited decreases in CAR, FC, LPC‑O, LPC, and LPE—lipids that increased in Leydig TM3 cells.

The fatty acid mixture produced similar effects on 6 lipid subclasses (CAR, DG, TG, LPC‑O, PC‑P, PE) in both cell types, but induced unique changes in FC, Hex2Cer, LPC, LPE, PC‑O, and PE‑O in Sertoli TM4 cells. In contrast, Leydig TM3 cells showed only a unique increase in HexCer, indicating a narrower sphingolipid‑related response (Fig. 3).

## Discussion

Obesity, metabolic syndrome, and lipid dysregulation are strongly linked to impaired male reproductive health, with elevated body mass index (BMI) consistently associated with reduced sperm quality (Chaudhuri et al. 2022). Sertoli cells rely heavily on lipid metabolism for energy production and membrane remodeling, and naturally accumulate lipid droplets during maturation, accompanied by rising TG and declining CER (Shi et al. 2018; Voigt et al. 2023a, b). Disruption of these pathways—including by EDCs such as obesogens—can impair Sertoli and Leydig cell number and function and contribute to idiopathic male infertility.

**Fig. 5:**
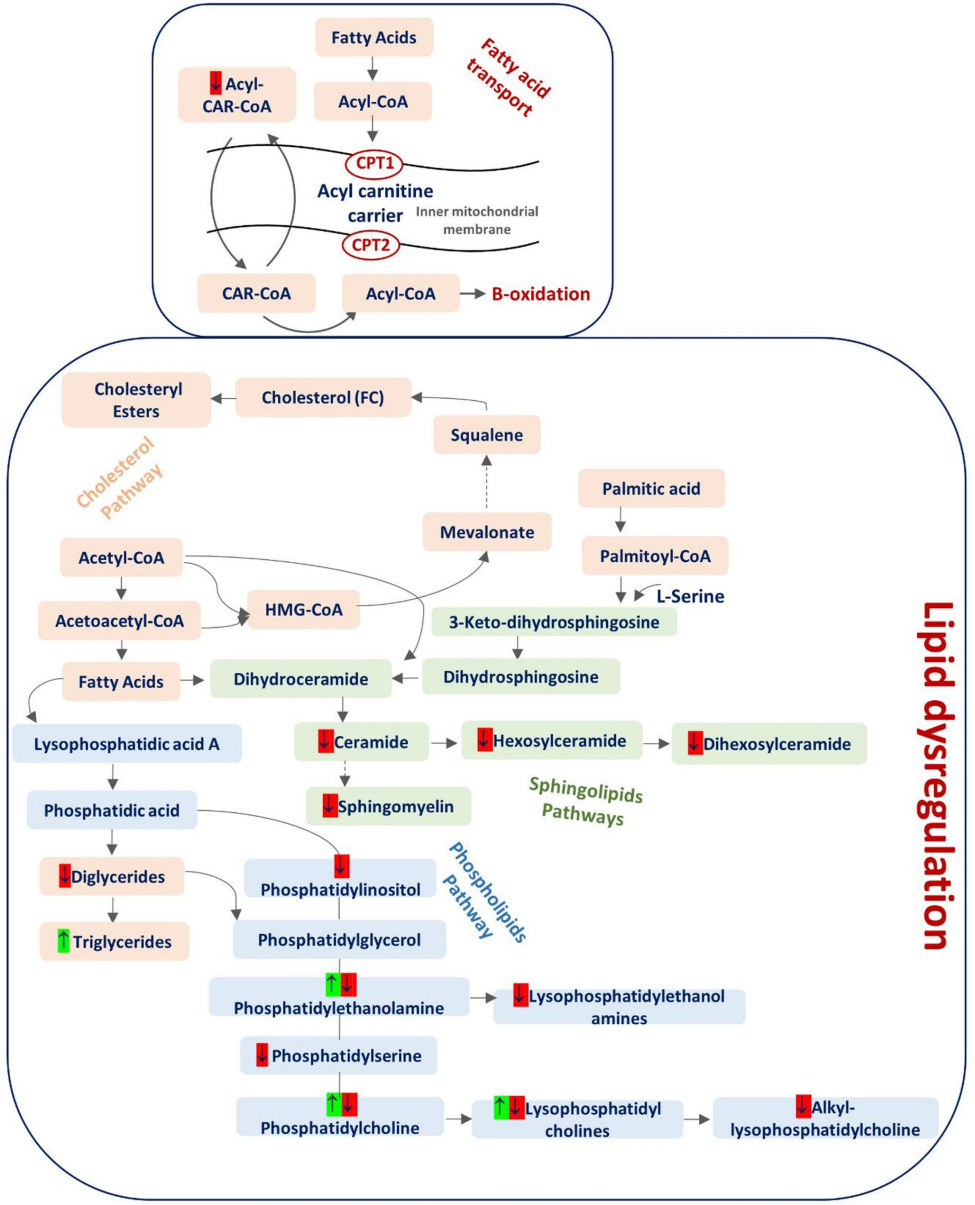
Proposed mechanism of OC-MIX-induced lipid disruption in Sertoli TM4 cells. A schematic summary illustrates how OC‑MIX triggers lipotoxicity and oxidative stress by dysregulating lipid metabolism. These effects appear to occur likely independently of androgen receptor (AR), peroxisome proliferator‑activated receptor‑α (PPARα), and aryl hydrocarbon receptor (AhR) signaling

Here, the environmentally relevant organochlorine mixture OC-MIX induced concentration- and time-dependent lipid accumulation and widespread dysregulation in Sertoli TM4 cells, accompanied by oxidative stress, reduced viability, and functional impairment relevant to spermatogenic support (Fig. 5). Amiodarone produced a similar lipotoxic signature, whereas the fatty acid mixture caused lipid dysregulation without overt cytotoxicity. These effects appear independent of AhR, AR, and PPARα pathways, despite known dioxin-like and antiandrogenic activities of OC-MIX (Virmani et al. 2026).

Fatty acid β-oxidation and the carnitine shuttle are essential for Sertoli cell metabolism, facilitating mitochondrial fatty acid transport and supporting energy production (Xiong et al. 2009; Reuter and Evans 2012). OC‑MIX reduced CAR levels, similar to amiodarone, suggesting increased β oxidation demand and carnitine depletion—paralleling findings in Leydig TM3 cells(Virmani et al. 2026) and PFOA (perfluorooctanoic acid)-treated mouse Leydig MLTC-1 cells (Huang et al. 2022). In contrast, the fatty acid mixture increased CAR, consistent with impaired β oxidation, as observed in OC-MIX-treated Leydig TM3 cells (Virmani et al. 2026) and palmitate-treated primary Sertoli cells, where CAR accumulation accompanied mitochondrial dysfunction and apoptosis (Luo et al. 2020; Xu et al. 2022).

Although all treatments induced neutral lipid accumulation, their lipidomic fingerprints diverged. OC-MIX elevated TG while reducing DG, producing a higher TG: DG ratio consistent with enhanced TG synthesis and reduced DG turnover, similar to protective responses in Sertoli cells exposed to palmitic acid (Ge et al. 2018) or mild hyperthermia (Vallés et al. 2014). The fatty acid mixture increased TG and DG, indicative of active lipid remodeling, whereas amiodarone minimally affected total TG and DG. Cholesterol homeostasis also differed: OC‑MIX altered neither FC nor CE, whereas the fatty acid mixture and amiodarone increased CE and shifted the CE: FC balance, which may impair membrane composition (Shi et al. 2018).

Glycerophospholipids, essential for membrane integrity and signaling (Hishikawa et al. 2014), showed clear treatment‑specific changes. Neither OC‑MIX nor amiodarone altered total PC, PE, or the PC/PE ratio, but both modulated several individual species. In contrast, the fatty acid mixture enhanced PE biosynthesis via LPE→PE and PS→PE conversions and lowered the PC: PE ratio by diverting PC toward DG. Although PC: PE imbalance is linked to metabolic disease (van der Veen et al. 2017), its relevance to testicular (patho)physiology remains unclear. OC‑MIX also markedly decreased total and individual LPE, PE‑O, LPC‑O, PS, and several LPC, PC‑O, and PC‑P species, even at 5 µg/mL. PI species, key regulators of membrane signaling and trafficking, and cell proliferation, survival, metabolism, and migration (Epand 2017; Wang et al. 2020), were consistently downregulated by OC‑MIX at ≥ 5 µg/mL, whereas amiodarone (2.5 µM) increased them, and the fatty acid mixture produced mixed effects.

Sphingolipids, essential for membrane structure and for regulating differentiation, apoptosis, and stress‑related signaling (Wang et al. 2020), also showed treatment-specific responses. OC‑MIX reduced HexCer, SM, and selected Hex2Cer and Cer species; amiodarone broadly increased species across these subclasses; the fatty acid mixture produced mixed effects, elevating some species while reducing others.

Comparisons with Leydig TM3 cells (Virmani et al. 2026) revealed cell type-specific vulnerabilities. Leydig TM3 cells had higher total lipid content, markedly elevated CAR (12-fold), and more abundant FC, consistent with their steroidogenic and metabolic demands. Although Sertoli TM4 cells contained less total lipid, lipid metabolism remains critical for their supportive role in spermatogenesis (Voigt et al. 2023a). These intrinsic differences likely underlie the broader lipid disruption, ROS generation, and protein loss observed in OC MIX–treated Leydig TM3 cells. In Sertoli TM4 cells, most lipid subclasses were downregulated, whereas many were upregulated in Leydig TM3 cells; TG was the only lipid subclass consistently increased in both cell types. Sertoli TM4 cells also showed unique alterations in phospholipids and lysophospholipids, suggesting impaired membrane remodeling and signaling, underscoring the importance of cell-specific lipid biology in testicular toxicity. Amiodarone showed similarly divergent effects. At 5 µM, it broadly altered lipid subclasses and induced moderate cytotoxicity in Leydig TM3 cells (Virmani et al. 2026), whereas Sertoli TM4 cells remained more resistant. Even at a non‑cytotoxic concentration (2.5 µM), amiodarone modulated different lipid subsets in each cell type—5 in Sertoli TM4 cells (CAR, FC, Hex2Cer, LPC O, LPE) and 3 in Leydig TM3 cells (SM, CER, PC-P)—demonstrating that cytotoxicity alone does not account for their differing lipid responses. The fatty acid mixture produced similar effects in both cell types with respect to neutral lipid accumulation, TG biosynthesis, and PC: PE remodeling, but uniquely altered several subclasses in Sertoli TM4 cells (FC, Hex2Cer, LPC, LPE, PC-O, PE-O), with particularly strong reductions in LPE and PC-O (~ 50%). Overall, the fatty acid mixture induced broader lipid alterations in Sertoli TM4 cells than in Leydig TM3 cells—opposite to the patterns observed with OC-MIX and amiodarone.

OC‑MIX disrupted lipid homeostasis in Sertoli TM4 cells at concentrations ≥ 5 µg/mL ( ~ ≥ 14 µM), corresponding to main individual organochlorine component levels (e.g., chlordane, p,p′ -DDT/DDE, PCBs) of ~ 2–3 µM. Highly exposed human populations frequently show serum levels of these compounds in the tens to hundreds of µg/L—and even higher in occupational settings—where they have been linked to adverse male reproductive outcomes (Bjerregaard and Hansen 2000; Ayotte et al. 2001; Martin et al. 2002; Aneck-Hahn et al. 2007; Brenda et al. 2009; Norie et al. 2010; Richardson et al. 2014; Bornman et al. 2018). Because these chemicals are highly lipophilic, serum concentrations likely underestimate testicular burdens. Indeed, residues such as HCB, HCH isomers, and p, p′ -DDE have been detected in human testes at 0.004–0.216 µg/g (Szymczynski and Waliszewski 1983), equivalent to ~ 4–208 µg/L assuming a tissue density of 1.038 g/mL (Handelsman and Staraj 1985). Thus, the exposure levels used here align with biologically relevant high-exposure scenarios.

In conclusion, OC MIX disrupted lipid metabolism in Sertoli TM4 cells at environmentally relevant concentrations, leading to oxidative stress and impaired cellular function. These lipid disturbances differed markedly from those observed in Leydig TM3 cells (Virmani et al. 2026), reflecting the distinct lipid compositions and physiological roles of the two testicular cell types. Such cell-specific lipid disruptions may contribute to testicular abnormalities, reduced sperm quality, male subfertility, and post-implantation losses reported in animal studies following prenatal or lactational OC MIX exposure (Anas et al. 2005; Maurice et al. 2018; Lessard et al. 2019). Similar divergences were noted for amiodarone and the fatty acid mixture, both associated with male reproductive dysfunction (Dobs et al. 1991; Bombil et al. 2024; George et al. 2024). Together, these findings support the concept that certain environmental contaminants and pharmaceuticals may act as testicular obesogens, contributing to adverse male reproductive outcomes through cell-specific, lipid-disrupting mechanisms.

## Supplementary Information

Below is the link to the electronic supplementary material.

Supplementary Material 1

## Data Availability

The datasets generated and/or analyzed during the current study are available from the corresponding author on reasonable request.
