# Supplementary material for: Lipid‑disrupting effects of an organochlorine mixture in Sertoli TM4 cells reveal testicular obesogen mechanisms
Source: Arch Toxicol. 2026 May 20;100(9):4199–211. doi: 10.1007/s00204-026-04446-4 (PMC13454004; doi:10.1007/s00204-026-04446-4)
Supplement: Supplementary file 1 [file 204_2026_4446_MOESM1_ESM.pdf]

Lipid-Disrupting Effects of an Organochlorine Mixture in Sertoli TM4 Cells Reveal Testicular Obesogen Mechanisms

*Ishita Virmani, Eliška Sychrová, Eliška Řehůřková, Darshak Gadara, Zdeněk Spáčil, Iva Sovadinová**

RECETOX, Faculty of Science, Masaryk University, Kotlarska 2, Brno, Czech Republic

*Corresponding author
Iva Sovadinová – RECETOX, Faculty of Science, Masaryk University, Kotlarska 2, Brno, Czech Republic, Tel.: (+420) 549 494 738, Fax: (+420) 549 49 2840
E-mail: iva.sovadinova@recetox.muni.cz

Table of Content **Pages**

Supplementary Text:

**Supplementary Text S1:** An overview of cell culturing	S3
**Supplementary Text S2:** An overview of cell viability assays	S3
**Supplementary Text S3:** An overview of ROS assay	S3
**Supplementary Text S4:** An overview of lipid accumulation assay	S3-S4
**Supplementary Text S5:** An overview of LC-MS/MS-based lipidome analysis	S4
**Supplementary Text S6:** An overview of RT-PCR conditions	S4

Supplementary Tables:

**Supplementary Table S1:** Chemical composition of the organochlorine mixture of interest, OC-MIX	S5
**Supplementary Table S2:** A list of regencies and materials used in this study	S6
**Supplementary Table S3:** Total numbers of detected and evaluated lipid classes in Sertoli TM4 cells	S7
**Supplementary Table S4:** Lipid concentration per protein (ng/µg) and percentage of total lipid for each lipid class in untreated Leydig TM3 (A.) and Sertoli TM4 (B.) cells	S8
**Supplementary Table S5:** Comparison of detected and evaluated lipid classes in Sertoli TM4 and Leydig TM3 cells	S9-10
**Supplementary Table S6:** List of the most active and suppressed reactions among lipid classes and individual lipid species identified by the BIOPAN tool	S11-S12

Supplementary Figures:

**Supplementary Figure S1:** Composition of the organochlorine mixture of interest, OC-MIX	S13
**Supplementary Figure S2:** Schematic representation of the experimental design involving direct interactions with murine non-tumorigenic Sertoli TM4 cells, a model of immature Sertoli cells	S14
**Supplementary Figure S3:** ROS production induced by menadione (MD, 100µM) in Sertoli TM4 cells after 1-h up to 48-h exposures	S15
**Supplementary Figure S4:** Induction of neutral lipid accumulation observed in Sertoli TM4 cells after 48-h exposure to amiodarone (AMIOD, 10 µM) and the fatty acid mixture (OAPA)	S16
**Supplementary Figure S5**: Representative images of neutral lipid accumulation induced by the mixture of oleic and palmitic acid (OAPA, 2:1 ratio, 100 µM) and amiodarone (AMIOD, 10 µM) in Sertoli TM4 cells along with the solvent control (DMSO 0.1%) after 48-h exposure	S17
**Supplementary Figure S6:** The impact of the organochlorine mixture OC-MIX and amiodarone (5 µM) and the fatty acid mixture (OAPA, 100 µM) on protein levels in Sertoli TM4 cells after 48 h	S17

Supplementary Figure S7: The impact of the organochlorine mixture (OC-MIX) on Sertoli cell TM4 viability determined by using a variety of conventional assays, including alamarBlue (AB), CFDA-AM, neutral red (NR), and MTT assays at 1 h, 24 h, and 117 h. S18
Supplementary Figure S8: Concentration-dependent lipid accumulation induced by amiodarone in Sertoli TM4 cells after 48-h exposures S19
Supplementary Figure S9: Induction of lipid droplet (LD) accumulation by the organochlorine mixture OC-MIX in Sertoli TM4 cells after 24- and 48-h exposures S19
Supplementary Figure S10: Expression of selected receptors in Sertoli TM4 cells and mouse adult testis at the mRNA level S20
Supplementary Figure S11: No significant neutral lipid accumulation in Sertoli TM4 cells after 48-h exposure to androgen receptor antagonist flutamide (1 µM), peroxisome proliferator-activated receptor alpha PPARα agonist GW76647 (1 µM), and aryl hydrocarbon receptor agonist TCDD (0.05 µM) S21
Supplementary Figure S12: Targeted lipidomic analysis of Sertoli TM4 cells compared to Leydig TM3 cells, focusing on individual lipid species levels S22
Supplementary Figure S13: Targeted lipidomic analysis of Sertoli TM4 cells exposed to flutamide (A.) and positive controls: amiodarone (2.5 µM (B.) and 5 µM (C.) and the mixture of oleic and palmitic acid (OAPA, 2:1 ratio, 100 µM) (D.) for 48 h, focusing on individual lipid species levels S23
Supplementary Figure S14: Targeted lipidomic analysis of Sertoli TM4 cells exposed to the organochlorine mixture OC-MIX for 48 h, focusing on individual lipid species levels at 2.5 (A.), 5 (B.), 10 (C.), and 20 µg/mL (D.). S24
Supplementary Figure S15: Changes in individual acylcarnitines (CAR) in Sertoli TM4 cells after 48-h exposure to the OC-MIX and control compounds. S25
Supplementary Figure S16: Impact of the organochlorine mixture OC-MIX along with negative and positive (OAPA, AMIOD) controls on individual neutral lipid species of cholesteryl esters (CE, A.), diacylglycerols (DGs, B.), and triacylglycerols (TGs, B.) — in Sertoli TM4 cells following 48-h exposure S26
Supplementary Figure S17: Impact of the organochlorine mixture OC-MIX along with negative and positive (OAPA, AMIOD) controls on individual lipid species of lipid subclasses — phosphatidylcholines (PCs, A.), phosphatidylethanolamines (PEs, B.), alkenylphosphatidylcholines (PC-P, C.), alkyl ether-linked phosphatidylcholines (PC-O, D.), and alkyl ether-linked phosphatidylethanolamines (PE-O, E.) — in Sertoli TM4 cells following 48-h exposure S27
Supplementary Figure S18: Impact of the organochlorine mixture OC-MIX along with negative and positive (OAPA, AMIOD) controls on individual lipid species of lipid subclasses — lysophosphatidylethanolamines (LPEs, A.), lysophosphatidylcholines (LPCs, B.), alkyl ether-linked lysophosphatidylcholines (LPC-O, C.), phosphatidylglycerols (PG, D.), phosphatidylinositols (PIs, E.), and phosphatidylserines (PSs, E.) — in Sertoli TM4 cells following 48-h exposure S28
Supplementary Figure S19: Impact of the organochlorine mixture OC-MIX along with negative and positive (OAPA, AMIOD) controls on individual lipid species of lipid subclasses — dihydroceramides (dhCers, A.), ceramides (Cers, B.), hexosylceramides (HexCers, C.), dihexosylceramides (Hex2Cers, D.), and sphingomyelins (SMs, E.) — in Sertoli TM4 cells following 48-h exposure S29
Supplementary Figure S20: Lipid pathway enrichment analysis in Sertoli TM4 cells treated with the organochlorine mixture (OC-MIX, 2.5 and 5 µg/mL) following 48-h exposure S30
Supplementary Figure S21: Lipid pathway enrichment analysis in Sertoli TM4 cells treated with amiodarone (AMIOD, 2.5 and 5 µM) following 48-h exposure S31
Supplementary Figure S22: Lipid pathway enrichment analysis in Sertoli TM4 cells treated with a mixture of fatty acids (OAPA) following 48-h exposure S32
Supplementary Figure S23: Differential lipid-disrupting effects of OC-MIX (A) and amiodarone (B, C) in Sertoli TM4 and Leydig TM3 cells S33
References S34

Supplementary Texts

Supplementary Text S1: An overview of cell culturing

Sertoli TM4 cells were cultivated following established protocols (Kubincová et al., 2019; Yawer et al., 2022) in DMEM/F12 with 5% horse serum, 2.5% fetal bovine serum, and 2.5 mM L-glutamine. The growth medium initially contained phenol red, but the exact composition without phenol red was used for exposure experiments. Cells were cultured in T25 flasks at 37°C with 5% CO_2 and regularly monitored for contamination, including mycoplasma testing. We used 0.25% trypsin-EDTA for cell passage every 2-3 days before reaching 80-90% confluency. All microplates and dishes were pre-coated with 0.1% (w/v) gelatin in PBS (phosphate-buffered saline (PBS: NaCl 137 mM, KCl 2.7 mM, Na_2HPO_4 10 mM, KH_2PO_4 7.4 mM; pH 7.4). To mitigate evaporation, only the inner 60 wells of the 96-well plates were used. Each microplate included negative and positive controls to minimize experimental condition differences.

Supplementary Text S2: An overview of cell viability assays

We continuously monitored cell behaviour using impedance-based technology with the xCELLigence RTCA SP instrument (ACEA Biosciences, San Diego, CA, USA), as detailed in our previous study (Virmani et al., 2026). At selected time points (1, 24, and/or 117 h), we assessed cell viability using resazurin (alamarBlue), CFDA-AM, neutral red uptake (NRU), and MTT assays (Sychrová et al., 2022; Virmani et al., 2026). Briefly, Sertoli TM4 cells were seeded at densities of 40,000 cells/cm^2, 20,000 cells/cm^2, and 17,000 cells/cm^2 for 1-, 24-, and 117-h exposure, respectively, reaching 80%, 50%, and 15-20% confluency before exposure. We conducted the RTCA (real-time cell analysis) experiment in a 96-well E-plate as described previously, the 3-dye combination assays (resazurin, CFDA-AM, and neutral red) in a black 96-well plate with a clear bottom, and the MTT assay in a transparent 96-well plate. Cells were seeded 24 h prior to OC-MIX exposure, followed by washing twice with 100 µL of PBS. We did the 3-dye combination assay and MTT assay, as previously mentioned (Sychrová et al., 2022; Virmani et al., 2026).

Supplementary Text S3: An overview of ROS assay

Briefly, we seeded cells at a density of 44,000 cells/cm^2 for 1- and 6-h exposures and 33,000 cells/cm^2 for 24- and 48-h exposures in a 96-well black plate with a transparent bottom (Greiner Bio-One, Frickenhausen, Germany, Cat. No. 655090). The cell confluency before exposure was 70% for the 1- and 6-h exposures and 50% for the 24- and 48-h exposures. Following exposure, cells were stained with 5 µM CellRoX™ Green reagent (Thermo Fischer Scientific, Waltham, Massachusetts, U.S., Cat. No. C10444) and 10 µg/mL Hoechst 33342 for 30 min. Fluorescence measurements for ROS (reactive oxygen species)-activated CellROX (excitation/emission: 485/520) and Hoechst nuclear staining (excitation/emission: 350/461) were done using a Synergy Microplate reader (Biotek, Winooski, Vermont, U.S.).

Supplementary Text S4: An overview of lipid accumulation assay

Neutral lipid accumulation was monitored using BODIPY™ 493/503 (Invitrogen, Cat No. 1987239), as previously published (Virmani et al., 2026). We used an image-based assay to evaluate lipid accumulation. Cells were seeded at a density of 44,000 cells/cm^2 for 24-h exposure and 33,000 cells/cm^2 for 48-h exposure in a 96-well black plate with a transparent bottom (Greiner Bio-One, Cat. No. 655090). Cell confluency before the 24 h exposure was 50% and increased to 80-90% at the time of imaging. For the 48-h exposure, confluency was 20-30% before exposure and reached 80-90% at the time of imaging. After 24 h of cultivation, cells were exposed to OC-MIX for 24 and 48 h. Following exposure, the media were discarded, and cells were rinsed twice with PBS. Cells were then fixed with 100 µL of 4% paraformaldehyde for 15 min at room temperature. Subsequently, cells were stained with BODIPY™ 493/503 (1.25 µg/mL) in the dark for 30 min. After two additional PBS washes, cells were co-stained with 1 µg/mL DAPI solution. Images were captured and analyzed using a BioTek Cytation 5 Imaging Multimode reader, as previously described (Virmani et al., 2026) Briefly, images were acquired using the Biotek Cytation 5 reader with a 20× objective, capturing a 3×3 montage in brightfield, DAPI (377/447 nm), and GFP (469/525 nm) channels. Cell counts were determined by quantifying DAPI-stained nuclei. Nine images (total area: 0.75 mm^2) per well were analyzed using Gen5 Prime 3.10 software (Biotek). Lipid accumulation was characterized by:

- **Droplet area per cell**: Sum of droplet areas (µm^2) divided by cell count.
- **Total signal per cell**: Integral of droplet signals divided by cell count.
- **Droplet count per cell**: Droplet count divided by cell count.
- **Average droplet area**: Droplet sum area divided by droplet count.

•**Average mean droplet intensity**: Sum of mean pixel intensity divided by droplet count.
•**Average integral droplet intensity**: Sum of pixel intensity divided by droplet count.

Supplementary Text S5: An overview of LC-MS/MS-based lipidome analysis

The experimental design mirrored that of the 48-h lipid accumulation assay, except that a transparent 96-well plate was used. Sample preparation and LC-MS/MS-based lipidome analysis followed the protocol described previously (Virmani et al., 2026). After 48-h exposure, cells were washed twice with ice-cold PBS. Lipids were extracted using 120 µL of isopropanol containing internal lipid standards (Cat. No. 330707, SPLASH LIPIDOMIX Mass Spec Standard, and LM6002, Cer/Sph Mixture I, Avanti Polar, Alabaster, AL, USA). The plate was sealed with a silicone lid, sonicated at 37 Hz for 15 min, and centrifuged at 2,880 rcf for 10 min. Then, 80 µL of the supernatant was transferred to a new 96-well plate.

The LC-MS analysis was performed as previously described (Gadara et al., 2024; Negi et al., 2023; Virmani et al., 2026) using a 1290 Infinity II UHPLC system coupled to a 6495 Triple Quadrupole mass spectrometer (Agilent, Santa Clara, CA, USA). Lipid extracts (1 µL) were injected twice onto a reverse-phase microbore column (CSH, 1 mm × 100 mm, 1.7 µm, Waters, Prague, Czechia) and separated at a flow rate of 100 µL/min over 15 min. The gradient elution used mobile phase A (10 mM ammonium formate in acetonitrile:water, 60:40) and mobile phase B (10 mM ammonium formate in isopropanol:acetonitrile, 90:10) with a specific gradient program: 0 min 15% B, 1.86 min 30% B, 2.32 min 48% B, 9.5 min 82% B, 12.5 to 13.5 min 99% B, and 13.5 to 15 min column re-equilibration.

The mass spectrometer operated in positive mode with specific source parameters: gas temperature at 200 °C, gas flow rate at 14 L/min, nebulizer pressure at 45 psi, sheath gas temperature at 400 °C, and sheath gas flow rate at 8 L/min. The capillary voltage was set to 4 kV, nozzle voltage to 500 V, and unit resolution was maintained for both Q1 and Q3. Data were acquired using dynamic multiple reaction monitoring (MRM) mode, with a 2-min retention time window for each transition. The raw data files were processed using Mass Hunter Quantitative analysis software (B.07.00, Agilent Technologies). SRM (selected-reaction monitoring)-based targeted lipid identification was performed using a prepared list of lipid species verified by a pooled sample quality control sample.

Supplementary Text S6: An overview of RT-PCR conditions

Sertoli TM4 cells were cultured to 60-80% confluence for RNA isolation. Mouse testis tissue from 6-month-old C57 males served as a control for gene expression analysis. Following (Virmani et al., 2026), total RNA from testis was isolated using TRI reagent® (Sigma Aldrich, Cat. No. T9424), and total RNA from Sertoli TM4 cells was isolated using the RNeasy Mini Kit (Qiagen, Cat. No. 74104). RNA quantity and quality were assessed using a Nanodrop spectrophotometer (ND 1000, Thermo Fisher Scientific). Primer details are in Virmani et al. (2026). cDNA synthesis was performed using the SensiFAST™ cDNA Synthesis Kit (Bioline Ltd, Cat. No. BIO-65053). PCR was run for 30 or 40 cycles in an automated thermocycler (TProfessional Thermocycler, Biometra, Göttingen, Germany). Gel electrophoresis was conducted, stained with ethidium bromide (0.5 µg/mL), and documented using the MF-ChemiBis gel imaging system (DNR Bio-Imaging Systems, Neve Yamin, Israel).

Supplementary Tables

Supplementary Table S1: Chemical composition of the organochlorine mixture of interest, OC-MIX. This table is adapted from our previous study (Virmani et al., 2026). A graphical representation of OC-MIX composition is in **Supplementary Figure S1**.

OC-MIX component	CAS No.	Company, Cat. No.	Purity (%)	MW	Weight[b] (%)	μg within 50 μg/mL OC-MIX[c]	μM within 50 μg/mL (=143 μM) OC-MIX[d]
**Technical Chlordane**	12789-03-6	Santa Cruz Biotechnology, Cat. No. Sc227594	99.0	409.76	**21.40**	**10.70**	**26.11**
***p,p'*-DDE** (*p,p'*-Dichlorodiphenyldichloroethylene)	72-55-9	Supelco, Cat. No. 49017	96.0	318.02	**19.30**	**9.70**	**30.34**
**Aroclor 1260**	11096-82-5	VWR, Cat. No. SUPL48704	CRM[a]	360.88	**19.08**	**9.54**	**26.43**
**Aroclor 1254**	11097-69-1	Sigma Aldrich, Cat. No. 48707	CRM[a]	326.43	**12.73**	**6.37**	**19.50**
***p,p'*-DDT** (*p,p'*- Dichlorodiphenyltrichloroethane)	50-29-3	Supelco, Cat. No. 31041	≥98.0	354.49	**6.79**	**3.40**	**9.60**
**Technical Toxaphene**	8001-35-2	VWR, Cat. No. EHERC10940000	100.0	413.81	**6.54**	**3.27**	**7.90**
**α-HCH** (α-Hexachlorocyclohexane)	319-84-6	Sigma Aldrich, Cat. No. 33856	≥98.0	290.83	**6.17**	**3.09**	**10.61**
**Aldrin**	309-00-2	VWR, Cat. No. 36666	≥98.0	364.90	**2.52**	**1.26**	**6.45**
**Dieldrin**	60-57-1	Merck, Cat. No. 33491	≥ 95%	380.91	**2.09**	**1.05**	**2.74**
**1,2,4,5-Tetrachlorobenzene**	95-94-3	VWR, Cat. No. EHERC17354500	98.5	215.89	**0.86**	**0.43**	**1.99**
***p,p'*-DDD** (1,1-Dichloro-2,2-bis(4-chlorophenyl)ethane)	72-54-8	Merck, Cat. No. 35486	≥98.0	320.03	**0.49**	**0.25**	**0.77**
**β-HCH** (β-Hexachlorocyclohexane)	319-85-7	Sigma Aldrich, Cat. No. 33376	98.0	290.80	**0.46**	**0.23**	**0.79**
**HCB** (*Hexachlorobenzene)*	118-74-1	Sigma Aldrich, Cat. No. 171050	99.0	284.80	**0.35**	**0.18**	**0.61**
**PCB28** (2,4,4'-trichlorobiphenyl)	7012-37-5	VWR, Cat. No. EHERC20002800	99.5	257.54	**0.32**	**0.16**	**0.63**
**PCB47** (2,2',4,4'-tetrachlorobiphenyl)	2437-79-8	VWR, Cat. No. EHERC20004700	99.0	291.98	**0.26**	**0.13**	**0.44**
**Mirex**	2385-85-5	Merck, Cat. No. 36170	≥95.0	545.51	**0.23**	**0.12**	**0.21**
**Lindane** (γ-HCH)	58-89-9	Sigma Aldrich, Cat. No. 233390	97.0	290.81	**0.20**	**0.10**	**0.34**
**Pentachlorobenzene**	608-93-5	Merck, Cat. No. 35886	≥98.0	250.32	**0.18**	**0.09**	**0.36**
**PCB126** (3,3',4,4',5-pentachlorobiphenyl)	57465-28-8	VWR, Cat. No. EHERC20012600	98.0	326.42	**0.006**	**0.003**	**0.01**
**PCB77** (3,3',4,4'-tetrachlorobiphenyl)	32598-13-3	VWR, Cat. No. EHERC20007700	99.5	291.98	**0.001**	**0.001**	**0.002**

[a] CRM, certified reference material; [b] Weight %, mixing proportion in a percentage for individual components of OC-MIX (sum = 100%); [c] μg of an individual mixture component within OC-MIX at the concentration of 50 μg/mL; [d] μM of an individual mixture component within OC-MIX at the concentration of 50 μg/mL (=143 μM)

Supplementary Table S2: A list of regencies and materials used in this study

Reagent/ Material	Vendor	Catalog No.
**96 well black plate with clear bottom**	Greiner Bio-One	655090
**96 well transparent plate**	TPP	92696
**96 well E-plate**	ACEA Biosciences	300600910
**alamarBlue™**	Invitrogen	DAL1025
**BODIPY™ 493/503**	Thermo Fischer Scientific	D3922
**CellRoX™ green reagent**	Thermo Fischer Scientific	C10444
**CFDA-AM (5-carboxyfluorescein diacetate acetoxymethyl ester (CFDA-AM)**	Invitrogen	C1354
**DAPI solution**	Sigma Aldrich	MBD0015
**DMEM-F12 w phenol red**	Sigma-Aldrich	D6434
**DMEM-F12 w/o phenol red**	Sigma-Aldrich	D6421
**DMSO**		
**Fetal Bovine Serum**	Biosera	FB1001
**Flutamide**	Sigma-Aldrich	F9397
**Gelatin**	Sigma-Aldrich	G9391
**GW76647**	Sigma-Aldrich	G6793-5MG
**Horse Serum**	Sigma-Aldrich	16050122
**Isopropanol**	Biosolve	162641
**L-Glutamine**	Biosera	XC-T1715
**MTT**	Sigma-Aldrich	M2128
**Neutral Red**	Sigma-Aldrich	N2889
**Oleic Acid**	Sigma Aldrich	01008
**Palmitic Acid**	Sigma Aldrich	P5585
**Paraformaldehyde**	Lach-Ner	40127-CP0-G1000-1
**RNeasy Mini Kit**	Qiagen	74104
**SensiFAST™ cDNA Synthesis Kit**	Bioline Ltd	65053
**T25 cell culture flasks**	TPP	90026
**TCDD**	LCG	CIL-ED-901-B
**TRI agent**	Sigma Aldrich	T9424
**Trypsin EDTA**	Biosera	LMT1706

Supplementary Table S3: **Total numbers of detected and evaluated lipid classes in Sertoli TM4 cells**

Lipid categories[a]	Lipid subclasses[a]	Abbreviation[a]	Detected lipid species[b]
Fatty Acyls	Acylcarnitines	CAR	9
Glycerolipids	Diacylglycerols	DG	11
	Triacylglycerols	TG	56
Sterol Lipids	Free cholesterol	FC	1
	Cholesteryl esters	CE	9
Glycerophospholipids	Phosphatidylcholines	PC	28
	Phosphatidylethanolamines	PE	18
	Ether-linked phosphatidylethanolamines	PE O	9
	Alkenylphosphatidylcholines	PC P	9
	Alkylphosphatidylcholines	PC O	20
	Lysophosphatidylethanolamines	LPE	3
	Lysophosphatidylcholines	LPC	9
	Alkyl ether-linked lysophosphatidylcholines	LPC O	3
	Phosphatidylglycerols	PG	6
	Phosphatidylinositols	PI	10
	Phosphatidylserines	PS	9
Sphingolipids	Dihydroceramides	dhCER	7
	Ceramides	CER	8
	Hexosylceramides	HexCER	6
	Dihexosylceramides	Hex2CER	5
	Sphingomyelins	SM	24
**Total**		**21**	**260**

[a] Lipid categories, subclasses, and their abbreviations according to LIPID MAPS(Fahy et al., 2009; Liebisch et al., 2020) / BioPAN as reported previously(Gaud et al., 2021; Huynh et al., 2019; Liebisch et al., 2013), [b] Number of all lipid species detected and evaluated in this study.

Supplementary Table S4: Lipid concentration per protein (ng/µg) and percentage of total lipid for each lipid class in untreated Leydig TM3 (A.) and Sertoli TM4 (B.) cells. Data are presented as mean ± SD from three independent experiments, each performed in quadruplicate (n = 12). Symbols (#) and red color indicate statistical significance (p≤0.05) from Leydig TM3 cells (non-parametric t-test, Mann-Whitney Rank Sum Test). Graphical presentations are in **Figure 2**.

	Fatty Acyls	Sterol Lipids		Glycero lipids		Sphingolipids					Glycerophospholipids											
	CAR	FC	CE	DG	TG	Cer	SM	dhCer	Hex Cer	Hex2 Cer	LPC	LPC -O	LPE	PC	PC -O	PC -P	PE	PE -O	PG	PI	PS	Sum
**A. Leydig TM3 cells**																						
ng lipid/ µg protein	0.04	33	19	3.5	26	0.26	19	0.15	0.09	0.11	3.6	0.24	0.18	64	13	4.7	50	1.8	0.92	34	11	284
	±0.02	±5.8	±6.3	±0.6	±2.8	±0.05	±5.3	±0.05	±0.03	±0.03	±1.1	±0.04	±0.04	±9.9	±5.6	±1.0	±15.4	±0.7	±0.14	±7.8	±1.8	±27
Order	21.	4.	7.	12.	5.	15.	6.	18.	20.	19.	11.	16.	17.	1.	8.	10.	2.	13.	14.	3.	9.	
	0.04	51		30		20					183											
	±0.02	±9.3		±3.0		±5.2					±17.5											
Order	5.	2.		3.		4.					1.											
%	0.014	11	6.6	1.2	9	0.09	6.7	0.05	0.03	0.04	1.25	0.09	0.06	23	4.6	1.7	17	0.62	0.33	12	3.7	100
	±0.006	±1.7	±1.9	±0.2	±1.2	±0.02	±1.6	±0.02	±0.01	±0.01	±0.31	±0.01	±0.01	±3.2	±1.8	±0.3	±4.8	±0.21	±0.05	±3.1	±0.5	
	0.014	18		11		7					65											
	±0.006	±2.3		±1.3		±1.5																
**B. Sertoli TM4 cells**																						
ng lipid/ µg protein	0.003#	25#	19	2.2#	26	0.08#	13.4#	0.09#	0.11	0.11	2.0#	0.30	0.34	40#	7.2#	1.9#	43	1.6	1.6#	14#	6.2#	204#
	±0.001	±4.8	±6.9	±0.8	±6.3	±0.03	±3.0	±0.03	±0.04	±0.03	±0.22	±0.07	±0.16	±8.5	±3.4	±0.05	±11.7	±0.54	±0.36	±3.3	±1.3	±27
Order	21.	4.	5.	10.	3.	20.	7.	19.	17.	18.	11.	16.	15.	2.	8.	12.	1.	14.	13.	6.	9.	
	0.003#	44#		28		14#					119#											
	±0.001	±7.9		±6.9		±3.1					±19.5											
Order	5.	2.		3.		4.					1.											
%	0.002#	12	10#	1.1	12#	0.04#	6.8	0.04	0.05#	0.05#	1.02	0.15#	0.16#	20	3.5#	0.91#	21	0.74	0.77#	7#	3.0#	100
	±0.001	±2.3	±3.5	±0.3	±2.3	±0.01	±0.9	±0.01	±0.01	±0.01	±0.22	±0.02	±0.06	±2.9	±1.5	±0.23	±4.2	±0.19	±0.13	±1.5	±0.5	
	0.002#	22#		14#		7					58											
	±0.001	±4.2		±2.4		±0.9					±3.6											

Supplementary Table S5: **Comparison of detected and evaluated lipid species in Sertoli TM4 and Leydig TM3 cells**. Lipid species unique to each testicular cell type are indicated in bold.

TM4	TM3	TM4	TM3	TM4	TM3
FC	FC	TG 54:3/NL-18:1	TG 54:3/NL-18:1	Hex2Cer(d18:1/24:0)	Hex2Cer(d18:1/24:0)
CAR18:1	CAR18:1	TG 54:4/NL-18:0	TG 54:4/NL-18:0	Hex2Cer(d18:1/24:1)	Hex2Cer(d18:1/24:1)
CAR12:0	CAR12:0	TG 54:4/NL-18:2	TG 54:4/NL-18:2	LPC 14:0	LPC 14:0
CAR14:1	CAR14:1	**TG 54:5/NL-18:1**		LPC 16:0	LPC 16:0
CAR14:0	CAR14:0	TG 54:6/NL-18:2	TG 54:6/NL-18:2	LPC 16:1	LPC 16:1
CAR15:0	CAR15:0	TG 56:0/	TG 56:0/	LPC 18:0	LPC 18:0
CAR16:0	CAR16:0	TG 56:3/NL-18:1	TG 56:3/NL-18:1	LPC 18:1	LPC 18:1
CAR16:1	CAR16:1	TG 56:4/NL-18:2	TG 56:4/NL-18:2	LPC 18:2	LPC 18:2
CAR18:0	CAR18:0	TG 56:5/NL-20:3	TG 56:5/NL-20:3	LPC 20:1	LPC 20:1
CAR 20:0	CAR 20:0	TG 56:6/NL-20:4	TG 56:6/NL-20:4	LPC 22:1	LPC 22:1
CE18:1	CE18:1	TG 56:7/NL-22:6	TG 56:7/NL-22:6	LPC 22:5	LPC 22:5
CE18:2	CE18:2	TG 58:1/	TG 58:1/	LPC O-16:0	LPC O-16:0
**CE18:3**		TG 58:3/	TG 58:3/	LPC O-18:0	LPC O-18:0
**CE20:2**		TG 58:8/NL-22:6	TG 58:8/NL-22:6	LPC O-18:1	LPC O-18:1
CE20:3	CE20:3	TG 60:1/	TG 60:1/	LPE 16:0	LPE 16:0
CE20:4	CE20:4	TG 60:3/	TG 60:3/	LPE 18:0	LPE 18:0
CE20:5	CE20:5	Cer(d16:1/16:0)	Cer(d16:1/16:0)	LPE 18:1	LPE 18:1
**CE22:5**		Cer(d18:1/16:0)	Cer(d18:1/16:0)	PC 28:0	PC 28:0
CE22:6	CE22:6		**Cer(d18:1/14:0)**	PC 30:0	PC 30:0
DG(14:0_16:0)	DG(14:0_16:0)		**Cer(d18:1/18:0)**	PC 30:1	PC 30:1
DG(16:0_16:0)	DG(16:0_16:0)		**Cer(d18:1/20:0)**	PC 32:0	PC 32:0
DG(16:0_16:1)	DG(16:0_16:1)	Cer(d18:1/22:0)	Cer(d18:1/22:0)	PC 32:1	PC 32:1
DG(16:0_18:1)	DG(16:0_18:1)	Cer(d18:1/23:0)	Cer(d18:1/23:0)	**PC 32:2**	
DG(16:0_18:2)	DG(16:0_18:2)	Cer(d18:1/24:0)	Cer(d18:1/24:0)		**PC 32:3**
DG(16:1_18:1)	DG(16:1_18:1)	Cer(d18:1/24:1)	Cer(d18:1/24:1)	PC 33:1	PC 33:1
DG(18:0_18:1)	DG(18:0_18:1)	Cer(d18:2/16:0)	Cer(d18:2/16:0)	PC 33:2	PC 33:2
DG(18:0_18:2)	DG(18:0_18:2)	Cer(d18:2/24:1)	Cer(d18:2/24:1)	PC 34:0	PC 34:0
DG(18:0_20:4)	DG(18:0_20:4)		**Cer(d18:2/18:0)**	PC 34:1	PC 34:1
DG(18:1_18:1)	DG(18:1_18:1)	SM 31:1	SM 31:1	PC 34:2	PC 34:2
DG(18:1_20:4)	DG(18:1_20:4)	SM 32:0	SM 32:0		**PC 34:3**
TG 42:0/NL-14:0	TG 42:0/NL-14:0	SM 32:1	SM 32:1		**PC 35:1**
TG 44:0/NL-14:0	TG 44:0/NL-14:0	SM 32:2	SM 32:2	PC 35:2	PC 35:2
TG 44:1/NL-14:0	TG 44:1/NL-14:0	SM 33:1	SM 33:1	PC 36:1	PC 36:1
TG 46:0/NL-14:0	TG 46:0/NL-14:0	SM 34:0	SM 34:0	PC 36:2	PC 36:2
TG 46:1/NL-14:0	TG 46:1/NL-14:0	SM 34:1	SM 34:1	PC 36:3	PC 36:3
TG 46:2/NL-14:0	TG 46:2/NL-14:0	SM 34:2	SM 34:2		**PC 36:4**
TG 48:0/NL-16:0	TG 48:0/NL-16:0	SM 35:1	SM 35:1	PC 36:5	PC 36:5
TG 48:1/NL-18:1	TG 48:1/NL-18:1	SM 36:1	SM 36:1	**PC 37:4**	
TG 48:2/NL-14:1	TG 48:2/NL-14:1	SM 37:1	SM 37:1	PC 38:1	PC 38:1
TG 48:2/NL-16:0	TG 48:2/NL-16:0	SM 38:1	SM 38:1	PC 38:2	PC 38:2
TG 48:2/NL-16:1	TG 48:2/NL-16:1		**SM 38:2**	PC 38:4	PC 38:4
TG 48:2/NL-18:1	TG 48:2/NL-18:1	SM 38:3	SM 38:3		**PC 38:5**
**TG 48:3/NL-16:1**		SM 39:1	SM 39:1	PC 38:6	PC 38:6
TG 48:3/NL-18:2	TG 48:3/NL-18:2	SM 40:0	SM 40:0	PC 38:7	PC 38:7
TG 49:1/NL-16:0	TG 49:1/NL-16:0	SM 40:1	SM 40:1	PC 40:1	PC 40:1
TG 49:1/NL-18:1	TG 49:1/NL-18:1	SM 40:2	SM 40:2	PC 40:2	PC 40:2
TG 50:0/NL-18:0	TG 50:0/NL-18:0		**SM 40:3**	PC 40:3	PC 40:3
TG 50:1/NL-14:0	TG 50:1/NL-14:0	SM 41:0	SM 41:0	PC 40:4	PC 40:4
TG 50:1/NL-18:1	TG 50:1/NL-18:1	SM 41:1	SM 41:1	PC 40:5	PC 40:5
TG 50:2/NL-18:0	TG 50:2/NL-18:0	SM 41:2	SM 41:2	PC 40:7	PC 40:7
TG 50:2/NL-18:1	TG 50:2/NL-18:1	SM 42:1	SM 42:1	PC O-28:0	PC O-28:0
TG 50:2/NL-18:2	TG 50:2/NL-18:2	SM 42:2	SM 42:2	PC O-30:0	PC O-30:0
TG 50:3/NL-14:1	TG 50:3/NL-14:1	SM 44:2	SM 44:2	PC O-30:1	PC O-30:1
TG 50:3/NL-16:1	TG 50:3/NL-16:1	SM 44:3	SM 44:3	PC O-32:0	PC O-32:0
TG 50:3/NL-18:1	TG 50:3/NL-18:1	dhCer(d18:0/16:0)	dhCer(d18:0/16:0)	PC O-32:1	PC O-32:1
**TG 51:1/NL-18:1**		dhCer(d18:0/18:0)	dhCer(d18:0/18:0)	PC O-32:2	PC O-32:2
TG 51:2/NL-15:0	TG 51:2/NL-15:0	dhCer(d18:1/18:0)	dhCer(d18:1/18:0)	PC O-34:0	PC O-34:0
TG 51:2/NL-16:0	TG 51:2/NL-16:0	dhCer(d18:1/20:0)	dhCer(d18:1/20:0)	PC O-34:1	PC O-34:1
TG 51:2/NL-18:1	TG 51:2/NL-18:1	dhCer(d18:1/22:0)	dhCer(d18:1/22:0)	PC O-34:2	PC O-34:2
TG 52:0/NL-16:0	TG 52:0/NL-16:0	dhCer(d18:1/24:0)	dhCer(d18:1/24:0)	PC O-36:1	PC O-36:1
TG 52:1/NL-18:0	TG 52:1/NL-18:0	dhCer(d18:1/24:1)	dhCer(d18:1/24:1)	PC O-36:2	PC O-36:2
TG 52:2/NL-16:0	TG 52:2/NL-16:0	HexCer(d18:1/16:0)	HexCer(d18:1/16:0)	PC O-36:4	PC O-36:4
TG 52:3/NL-16:1	TG 52:3/NL-16:1	HexCer(d18:1/18:0)	HexCer(d18:1/18:0)	PC O-36:6	PC O-36:6
TG 52:3/NL-18:2	TG 52:3/NL-18:2	**HexCer(d18:1/20:0)**		PC O-38:1	PC O-38:1
TG 52:4/NL-16:0	TG 52:4/NL-16:0	HexCer(d18:1/22:0)	HexCer(d18:1/22:0)	PC O-38:2	PC O-38:2
TG 52:4/NL-18:1	TG 52:4/NL-18:1	HexCer(d18:1/24:0)	HexCer(d18:1/24:0)	PC O-38:3	PC O-38:3
TG 53:2/NL-17:0	TG 53:2/NL-17:0	HexCer(d18:1/24:1)	HexCer(d18:1/24:1)	**PC O-38:4**	
TG 54:0/NL-18:0	TG 54:0/NL-18:0	Hex2Cer(d18:1/16:0)	Hex2Cer(d18:1/16:0)	PC O-38:5	PC O-38:5
TG 54:1/NL-18:1	TG 54:1/NL-18:1	Hex2Cer(d18:1/18:0)	Hex2Cer(d18:1/18:0)	PC O-38:6	PC O-38:6
TG 54:2/NL-18:0	TG 54:2/NL-18:0	Hex2Cer(d18:1/22:0)	Hex2Cer(d18:1/22:0)	PC O-40:5	PC O-40:5

TM4	TM3
PC P-30:0	PC P-30:0
PC P-32:0	PC P-32:0
PC P-32:1	PC P-32:1
PC P-34:0	PC P-34:0
PC P-34:1	PC P-34:1
**PC P-34:2**	
PC P-36:3	PC P-36:3
PC P-36:4	PC P-36:4
PC P-38:5	PC P-38:5
PE 32:0	PE 32:0
PE 32:1	PE 32:1
PE 34:1	PE 34:1
PE 34:2	PE 34:2
	**PE 34:3**
PE 35:1	PE 35:1
PE 35:2	PE 35:2
PE 36:1	PE 36:1
PE 36:2	PE 36:2
PE 36:3	PE 36:3
PE 38:2	PE 38:2
PE 38:3	PE 38:3
PE 38:4	PE 38:4
PE 38:6	PE 38:6
PE 40:3	PE 40:3
PE 40:4	PE 40:4
PE 40:5	PE 40:5
PE 40:6	PE 40:6
PE 40:7	PE 40:7
PE O-34:2	PE O-34:2
PE O-36:2	PE O-36:2
PE O-36:5	PE O-36:5
**PE O-38:6**	
PE O-38:7	PE O-38:7
PE O-40:4	PE O-40:4
PE O-40:6	PE O-40:6
PE O-40:7	PE O-40:7
PE O-40:8	PE O-40:8
**PG 32:1**	
PG 34:1	PG 34:1
PG 34:2	PG 34:2
PG 36:2	PG 36:2
PG 36:3	PG 36:3
PG 36:4	PG 36:4
PI 32:1	PI 32:1
PI 34:1	PI 34:1
PI 36:1	PI 36:1
	**PI 36:2**
**PI 36:3**	
PI 36:4	PI 36:4
	**PI 38:3**
PI 38:4	PI 38:4
PI 38:5	PI 38:5
PI 38:6	PI 38:6
**PI 40:4**	
PI 40:5	PI 40:5
	**PI 40:6**
PS 34:1	PS 34:1
PS 34:2	PS 34:2
PS 36:1	PS 36:1
PS 36:2	PS 36:2
PS 38:3	PS 38:3
PS 38:4	PS 38:4
PS 40:4	PS 40:4
PS 40:5	PS 40:5
PS 40:6	PS 40:6

Supplementary Table S6: List of the most active and suppressed reactions among lipid classes and individual lipid species identified by the BIOPAN tool

Chemicals	Most active reaction	Most suppressed reaction
**OAPA (100 µM) *vs.* SC**	•**PC**(30:0), (32:0), (32:1), (34:1), (34:2), (36:1), (36:2), (38:4) → **DG**(30:0), (32:0), (32:1), (34:1), (34:2), (36:1), (36:2), (38:4) → **TG**(44:0), (46:0), (46:1), (48:0), (48:1), (48:2), (50:0), (50:1), (52:0), (52:1), (54:0), (54:1), (50:2), (54:5), (56:0), (50:3), (52:2), (54:2), (54:6), (52:3), (56:6), (58:1), (52:4), (54:3), (56:3), (56:7), (58:3), (60:1), (54:4), (60:3), (56:4), (56:5)<br>•**LPE**(16:0), (18:0), (18:1) → **PE**(32:0), (32:1), (34:1), (34:2), (35:2), (36:1), (36:2), (36:3), (40:5), (36:3), (38:2), (40:6), (38:6), (38:4), (40:3), (40:4), (40:7)<br>•**PE**(32:0), (32:1), (34:1), (34:2), (35:2), (36:1), (36:2), (36:3), (38:2), (38:4), (38:4), (38:6), (40:3), (40:4), (40:5), (40:7) → **PC**(32:0), (32:1), (34:1), (34:2), (35:2), (36:1), (36:2), (36:3), (38:2), (38:4), (38:6), (40:3), (40:4), (40:5), (40:7)<br>•**PS**(34:1), (34:2), (36:1), (36:2), (38:3), (38:4), (40:4), (40:5), (40:6) → **PE**(34:1), (34:2), (36:1), (36:2), (38:3), (38:4), (40:4), (40:5), (40:6)<br>•**O-LPC**(16:0), (18:0), (18:1) → **O-PC**(30:0), (32:0), (32:1), (34:0), (34:1), (34:2), (36:1), (38:1), (38:5), (36:2), (40:5), (38:2)	•**TG**(46:0), (48:0), (48:1), (50:1), (50:2), (52:1), (52:2), (54:4), (54:5) → **DG**(30:0), (32:0), (32:1), (34:1), (34:2), (36:1), (36:2), (38:4), (38:5)<br>•**DG**(30:0), (32:0), (32:1), (34:1), (34:2), (36:1), (36:2), (38:4) → **PC**(30:0), (32:0), (32:1), (34:1), (34:2), (36:1), (36:2), (38:4)<br>•**PC**(34:1), (34:2), (36:1), (36:2), (38:4), (40:4), (40:5) →**PS**(34:1), (34:2), (36:1), (36:2), (38:4), (40:4), (40:5)<br>•**PS**(34:1), (34:2), (36:1), (36:2), (38:3), (38:4), (40:4), (40:5), (40:6) → **PE**(34:1), (34:2), (36:1), (36:2), (38:3), (38:4), (40:4), (40:5), (40:6)<br>•**PE**(32:0), (34:1) → **LPE**(16:0), (18:0), (18:1)<br>•**O-PC**(32:0), (34:0), (34:1) → **O-LPC**(16:0), (18:0), (18:1)
**AMIOD (2.5 µM) *vs.* SC**	•**LPC**(14:0), (16:0), (16:1), (18:0), (18:1), (18:2) → **PC**(28:2), (30:0), (30:1), (32:0), (32:1), (32:2), (34:0), (34:1), (36:1), (36:5), (38:1), (34:2), (40:1), (36:2), (38:2), (38:6), (40:2), (40:5), (36:3), (40:3), (40:7)<br>•**PC**(34:1), (34:2), (36:1), (36:2), (38:4), (40:4), (40:5) → **PS**(34:1), (34:2), (36:1), (36:2), (38:4), (40:4), (40:5)<br>•**PS**(34:1), (34:2), (36:1), (36:2), (38:3), (38:4), (40:4), (40:5), (40:6) → **PE**(34:1), (34:2), (36:1), (36:2), (38:3), (38:4), (40:4), (40:5), (40:6)<br>•**O-LPC**(16:0), (18:0), (18:1) → **O-PC**(30:0), (32:0), (32:1), (34:0), (34:1), (34:2), (36:1), (38:1), (38:5), (36:2), (40:5), (38:2)	•**PC**(30:0), (32:0), (32:1), (34:0), (34:1), (34:2), (36:1), (38:1), (38:6) → **LPC**(14:0), (16:0), (16:1), (18:0), (18:1), (18:2), (20:1), (22:1), (22:5)
**AMIOD (5 µM) *vs.* SC**	•**PC**(34:1), (34:2), (36:1), (36:2), (38:4), (40:4), (40:5) → **PC**(34:1), (34:2), (36:1), (36:2), (38:4), (40:4), (40:5)<br>•**PE**(32:0), (34:1) → **LPE**(16:0), (18:0), (18:1)	•**LPC**(14:0), (16:0), (16:1), (18:0), (18:1), (18:2) → **PC**(28:2), (30:0), (30:1), (32:0), (32:1), (32:2), (34:0), (34:1), (36:1), (36:5), (38:1), (34:2), (40:1), (36:2), (38:2), (38:6), (40:2), (40:5), (36:3), (40:3), (40:7)<br>•**PC**(30:0), (32:0), (32:1), (34:1), (34:2), (36:1), (36:2), (38:4) → **DG**(30:0), (32:0), (32:1), (34:1), (34:2), (36:1), (36:2), (38:4) → **TG**(44:0), (46:0), (46:1), (48:0), (48:1), (48:2), (50:0), (50:1), (52:0), (52:1), (54:0), (54:1), (50:2), (56:5), (56:0), (50:3), (52:2), (54:2), (54:2), (54:6), (52:3), (56:6), (58:1), (52:4), (54:3), (56:3), (56:7), (58:3), (60:1), (54:4), (60:3), (56:4), (56:5)<br>•**O-LPC**(16:0), (18:0), (18:1) → **O-PC**(30:0), (32:0), (32:1), (34:0), (34:1), (34:2), (36:1), (38:1), (38:5), (36:2), (40:5), (38:2)
**OC-MIX (2.5 µg/mL) *vs.* SC**	*None identified*	•**PE**(32:0), (32:1), (34:1), (34:2), (35:2), (36:1), (36:2), (36:3), (38:2), (38:4), (38:6), (40:3), (40:4), (40:5), (40:7) → **PC**(32:0), (32:1), (34:1), (34:2), (35:2), (36:1), (36:2), (36:3), (38:2), (38:4), (38:6), (40:3), (40:4), (40:5), (40:7)
**OC-MIX (5 µg/mL) *vs.* SC**	•**O-PC**(32:0), (34:0), (34:1) → **O-LPC**(16:0), (18:0), (18:1)	•**LPC**(14:0), (16:0), (18:0), (18:1), (18:2) → **PC**(28:0), (30:0), (30:1), (32:0), (32:1), (32:2), (34:0), (34:1), (36:1), (36:5), (38:1), (34:2), (40:1), (36:2), (38:2), (38:6), (40:2), (40:5), (36:3), (40:3), (40:7)

		•**O-LPC**(16:0), (18:0), (18:1) → **O-PC**(30:0), (32:0), (32:1), (34:0), (34:1), (34:2), (36:1), (38:1), (38:5), (36:2), (40:5), (38:2)
**OC-MIX (10 µg/mL) *vs*. SC**	*None identified*	•**PC**(34:1), (34:2), (36:1), (36:2), (38:4), (40:4), (40:5) → **PS**(34:1), (34:2), (36:1), (36:2), (38:4), (40:4), (40:5)<br>•PE (32:0), (34:1) → LPE (16:0), (18:0), (18:1)
**OC-MIX (20 µg/mL) *vs*. SC**	•**DG**(30:0), (32:0), (32:1), (34:1), (34:2), (36:1), (36:2), (38:4) → **PC**(30:0), (32:0), (32:1), (34:1), (34:2), (36:1), (36:2), (38:4)<br>•**PC**(30:0), (32:0), (32:1), (34:0), (34:1), (34:2), (36:1), (38:1), (38:6) → **LPC**(14:0), (16:0), (16:1), (18:0), (18:1), (18:2), (20:1), (22:1), (22:5)<br>•**O-LPC**(16:0), (18:0), (18:1) → **O-PC**(30:0), (32:0), (32:1), (34:0), (34:1), (34:2), (36:1), (38:1), (38:5), (36:2), (40:5), (38:2)<br>•**LPE**(16:0), (18:0), (18:1) → **PE**(32:0), (32:1), (34:1), (34:2), (36:1), (36:2), (40:5), (36:3), (38:2), (40:6)<br>•**PE**(32:0), (32:1), (34:1), (34:2), (35:2), (36:1), (36:2), (36:3), (38:2), (38:4), (38:6), (40:3), (40:4), (40:5), (40:7) → **PC**(32:0), (32:1), (34:1), (34:2), (35:2), (36:1), (36:2), (36:3), (38:2), (38:4), (38:6), (40:3), (40:4), (40:5), (40:7)<br>•**PS**(34:1), (34:2), (36:1), (36:2), (38:3), (38:4), (40:4), (40:5), (40:6) → **PE**(34:1), (34:2), (36:1), (36:2), (38:3), (38:4), (40:4), (40:5), (40:6)	•**PC**(30:0), (32:0), (32:1), (34:1), (34:2), (36:1), (36:2), (38:4) → **DG**(30:0), (32:0), (32:1), (34:1), (34:2), (36:1), (36:2), (38:4)<br>•**PE**(32:0), (34:1) → **LPE**(16:0), (18:0), (18:1)<br>•**TG**(46:0), (48:0), (48:1), (50:1), (50:2), (52:1), (52:2), (54:4), (54:5) → **DG**(30:0), (32:0), (32:1), (34:1), (34:2), (36:1), (36:2), (38:4), (38:5)<br>•**O-PC**(32:0), (34:0), (34:1) → **O-LPC**(16:0), (18:0), (18:1)

Supplementary Figures

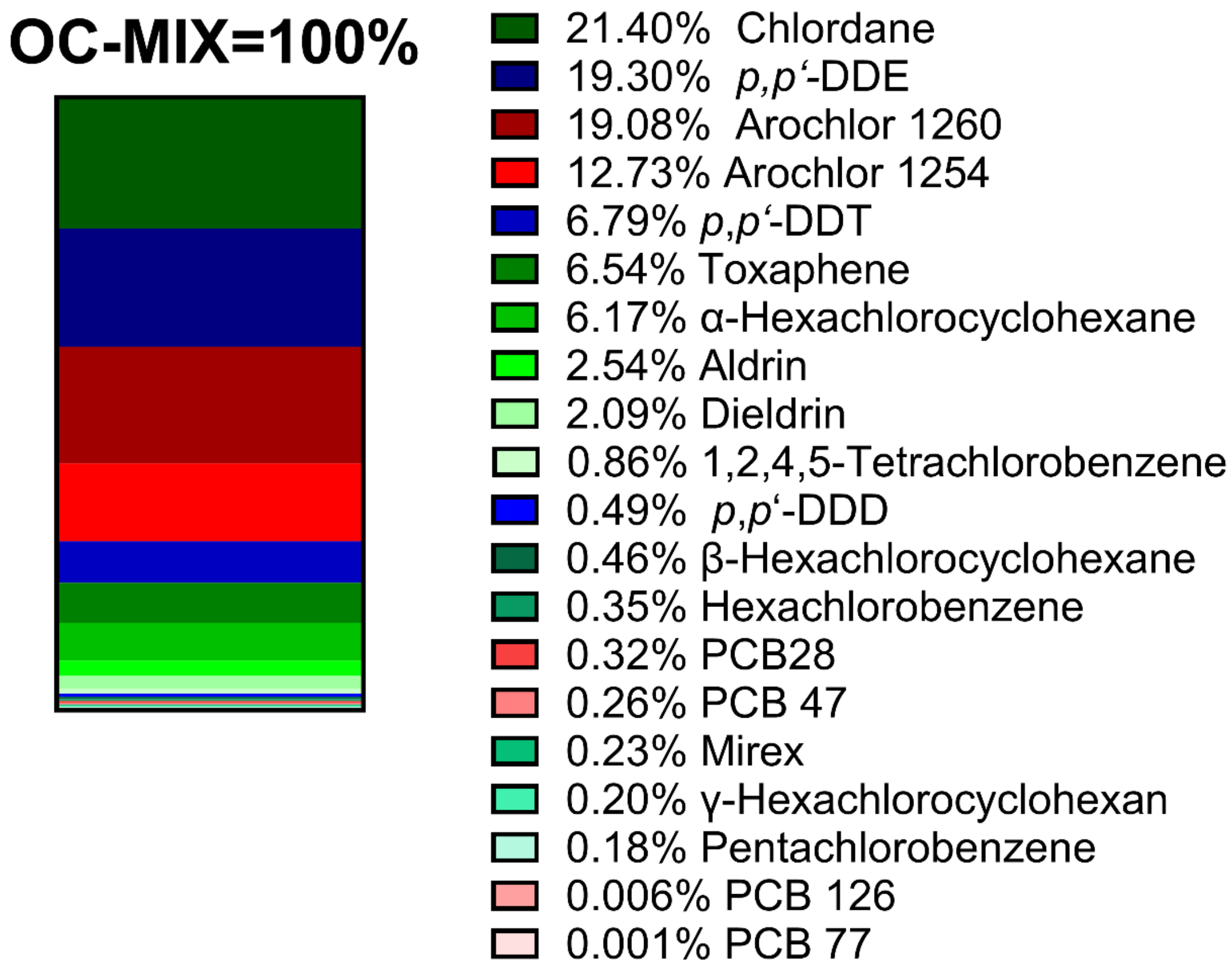

Supplementary Figure S1: **Composition of the organochlorine mixture of interest, OC-MIX.** The figure illustrates the weight percentages and mixing proportions of individual components in OC-MIX, summing to 100%. Green and its shades denote organochlorine pesticides, blue and its shades represent their metabolites, and red and its shades indicate polychlorinated biphenyls (PCBs). Detailed characterizations of each component are provided in **Supplementary Table S1**.

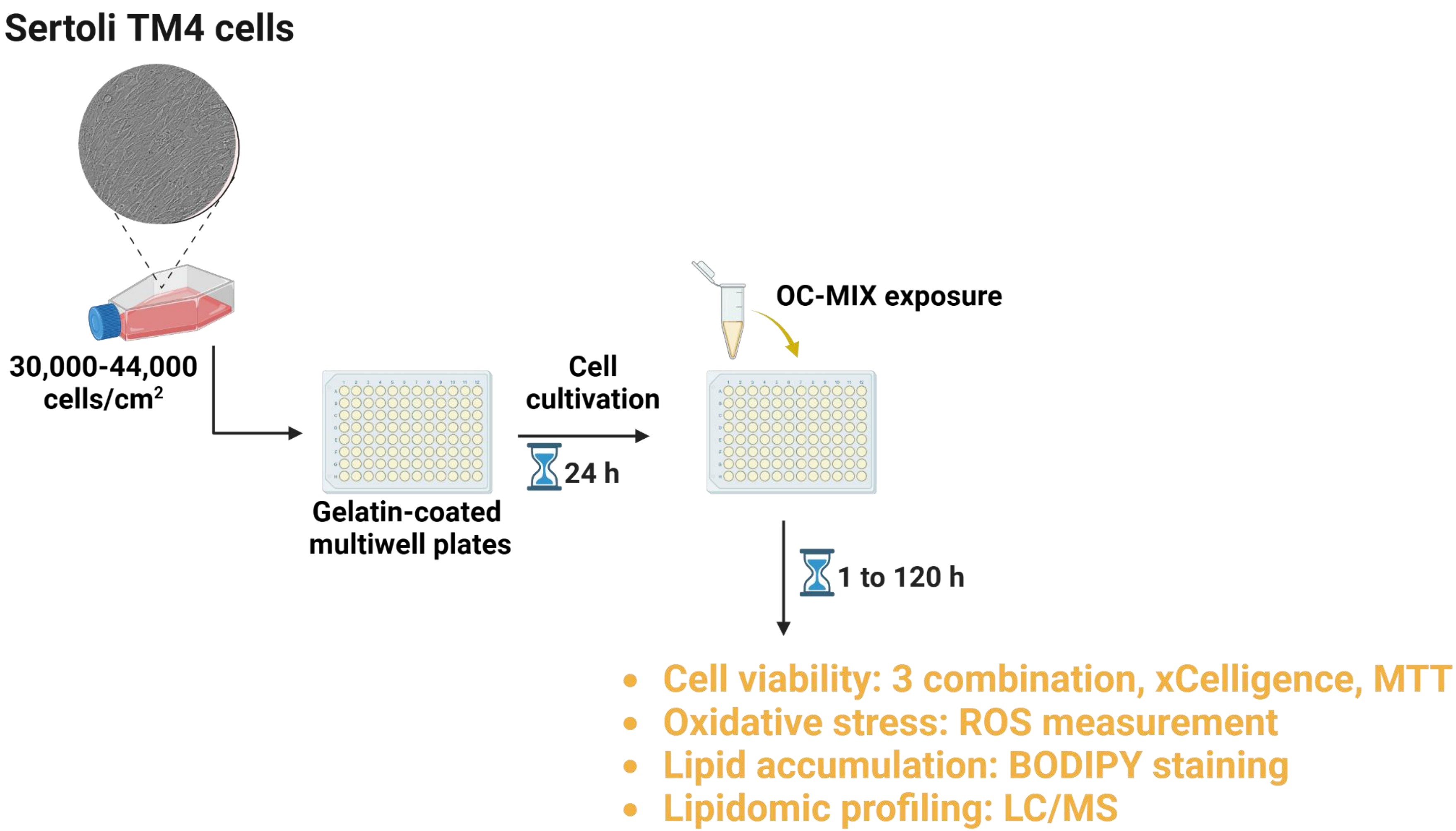

Supplementary Figure S2: Schematic representation of the experimental design involving direct interactions with murine non-tumorigenic Sertoli TM4 cells, a model of immature Sertoli cells. Experiment validity was assessed using predefined negative and positive controls, including criteria for cell contamination, unexpected cell death, equipment failures, or errors. The figure was created with BioRender.com.

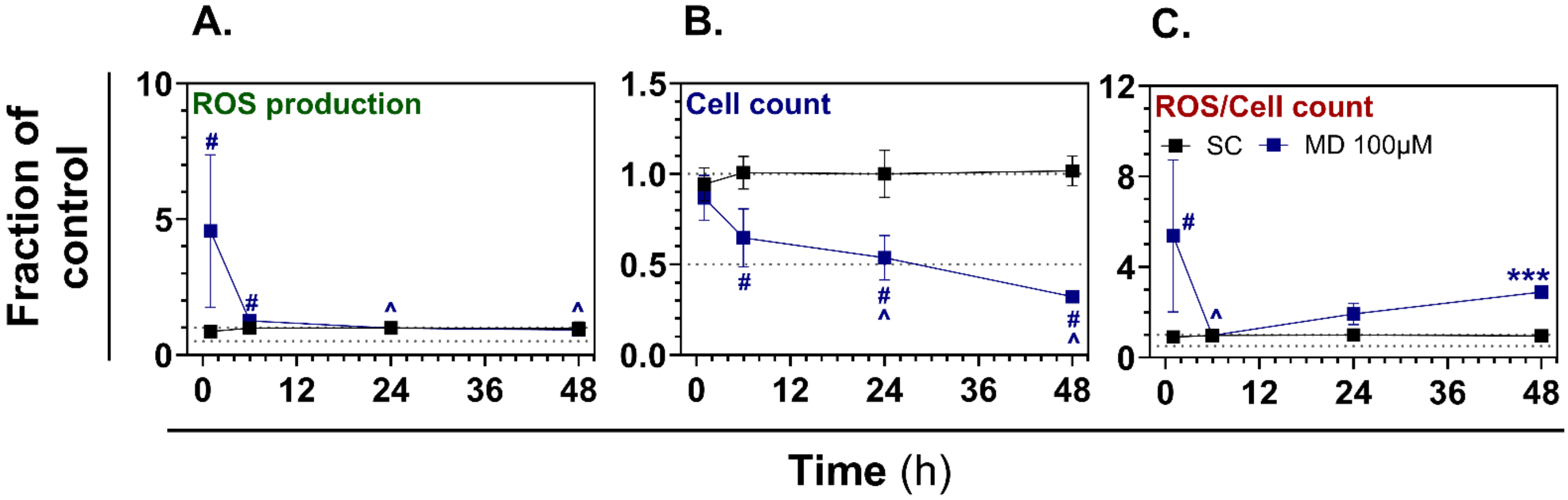

Supplementary Figure S3: **ROS production induced by menadione (MD, 100 µM) in Sertoli TM4 cells after 1-h up to 48-h exposures**. ROS (reactive oxygen species) production was determined by staining with CellRoX™ Green reagent, with nuclei counterstained with Hoechst 33342. The mean and standard deviation (SD) are shown from minimal 3 independent experiments. The asterisk (*) and hashtag (#) denote statistically significant ($p \leq 0.05$) changes compared to the appropriate solvent control (**SC**, DMSO 0.1%), and ^ denotes changes compared to the 1-h exposure (***, $p < 0.001$, one-way ANOVA followed by Dunnett's test # ^, $p < 0.05$, Kruskal-Wallis ANOVA on ranks followed by Dunnet's or Dunn's test).

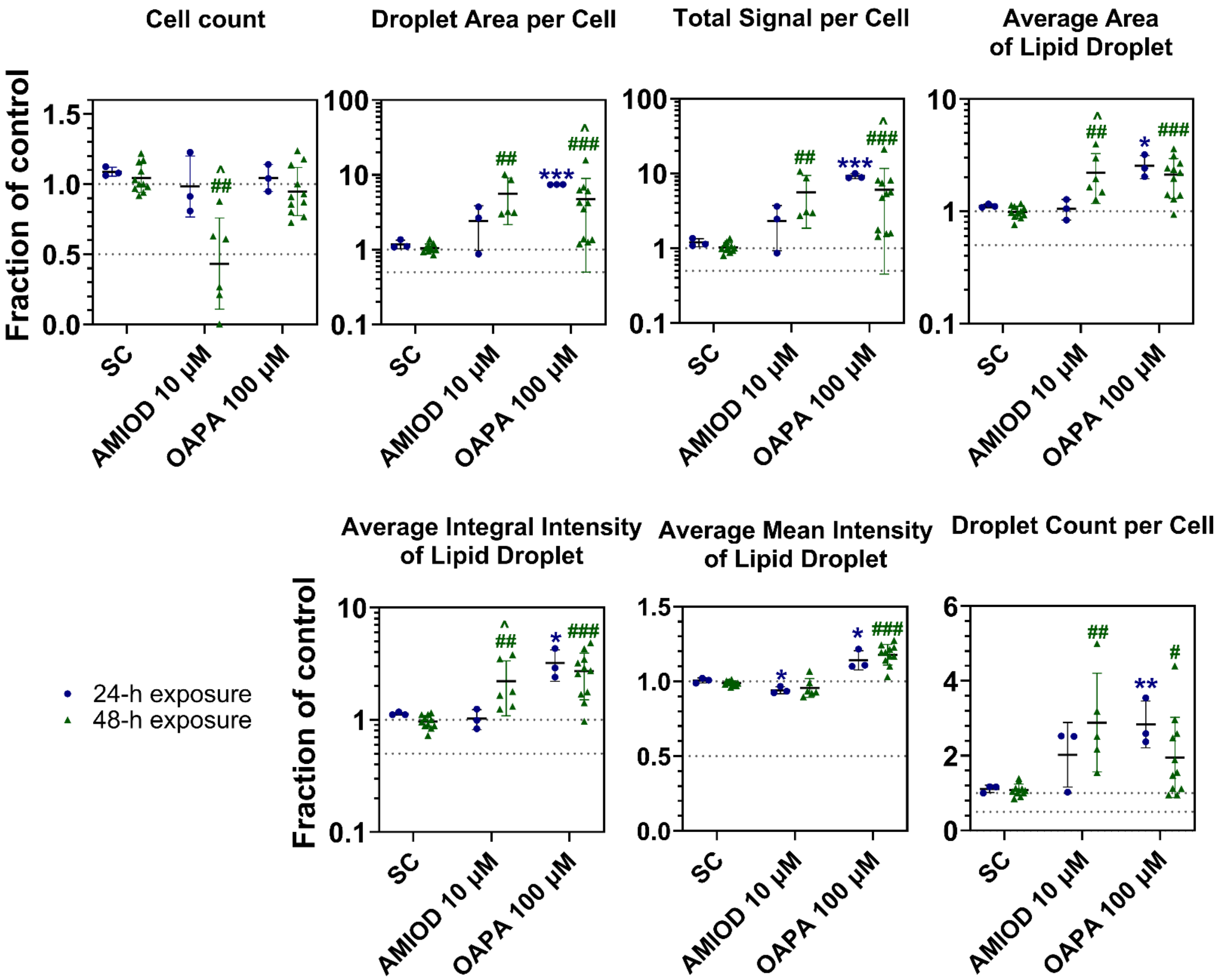

Supplementary Figure S4: **Induction of neutral lipid accumulation observed in Sertoli TM4 cells after 24-h and 48-h exposures to amiodarone (AMIOD, 10 µM) and the fatty acid mixture (OAPA, 100 µM).** Neutral lipid accumulation was detected using BODIPY 493/503 staining, while nuclei were co-stained with DAPI. Representative images are in **Supplementary Figure S5**. OAPA consists of oleic acid and palmitic acid in a 2:1 ratio. The figures display the mean and standard deviation (SD) from at least three independent experiments, along with individual data points. Statistically significant changes are denoted by asterisks (*) and hashtags (#): *p≤0.05 compared to the appropriate solvent control (SC, DMSO 0.1%), and ^ indicates changes relative to the 1-h exposure (***p < 0.001, one-way ANOVA followed by Dunnet's test; #^p < 0.05, Kruskal-Wallis ANOVA on ranks followed by Dunnet's or Dunn's test).

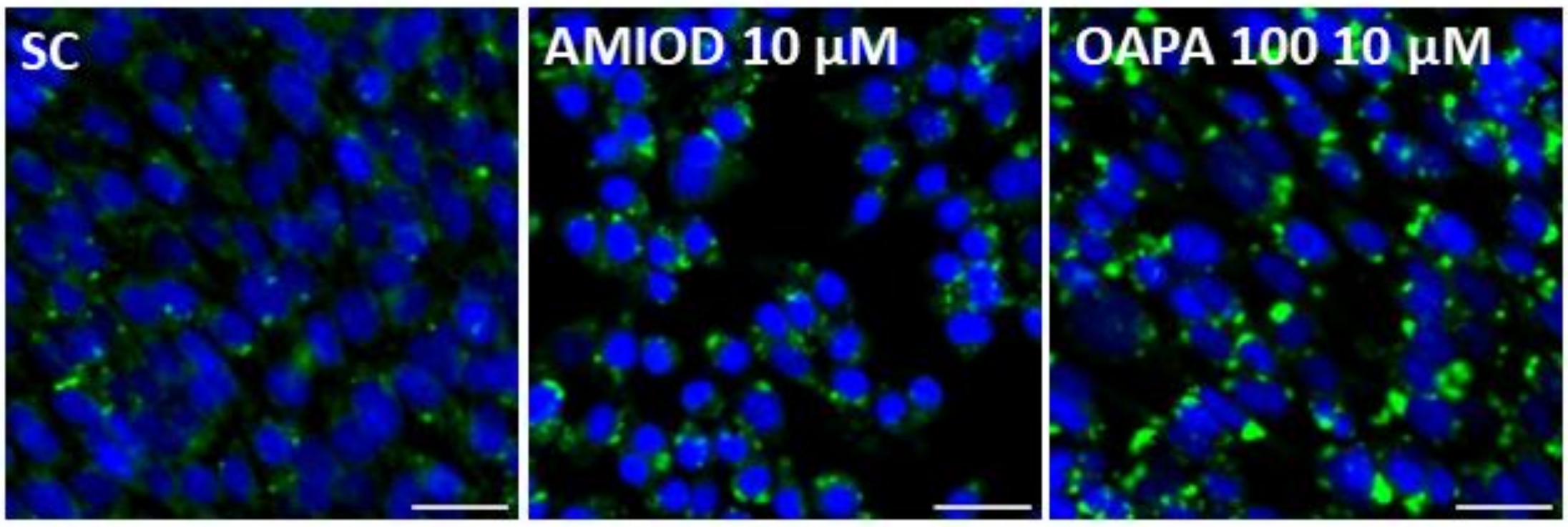

Supplementary Figure S5: **Representative images of neutral lipid accumulation induced by the mixture of oleic and palmitic acid (OAPA, 2:1 ratio, 100 μM) and amiodarone (AMIOD, 10 μM) in Sertoli TM4 cells along with the solvent control (DMSO 0.1%) after 48-h exposure**. Neutral lipid accumulation was determined by staining the lipid droplets with BODIPY™ 493/503 (**green**), with nuclei counterstained using DAPI (**blue**). Scale bar = 30 μm.

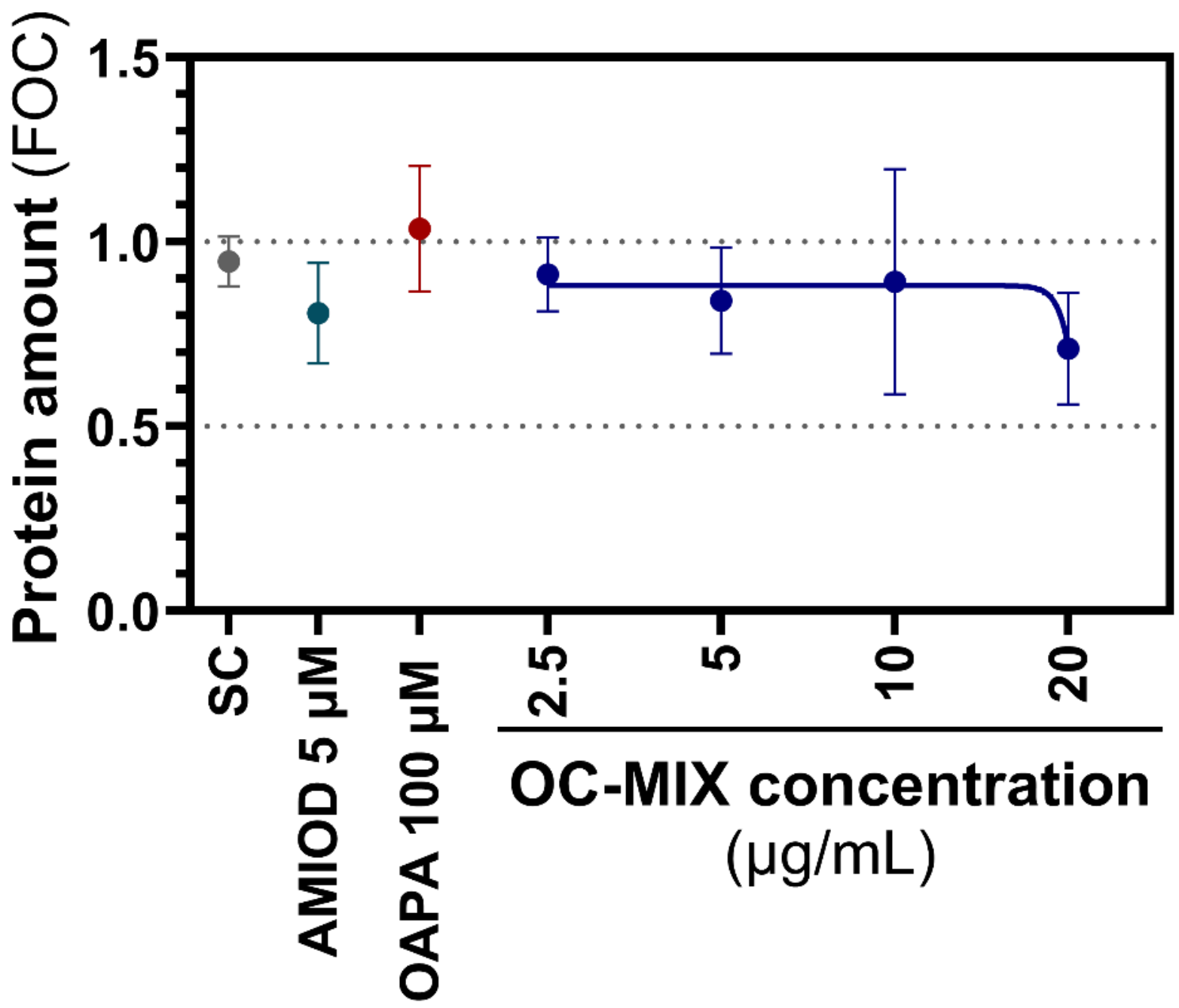

Supplementary Figure S6: The impact of the organochlorine mixture OC-MIX and amiodarone (5 μM) and the fatty acid mixture (OAPA, 100 μM) on protein levels in Sertoli TM4 cells after 48 h. Protein quantification was evaluated using a BCA (bicinchoninic acid) protein assay kit. The data were normalized to untreated cells and expressed as a fraction of control (FOC). No statistically significant differences from the solvent control (0.1% DMSO) were observed for any treatment ($p > 0.05$, one-way ANOVA).

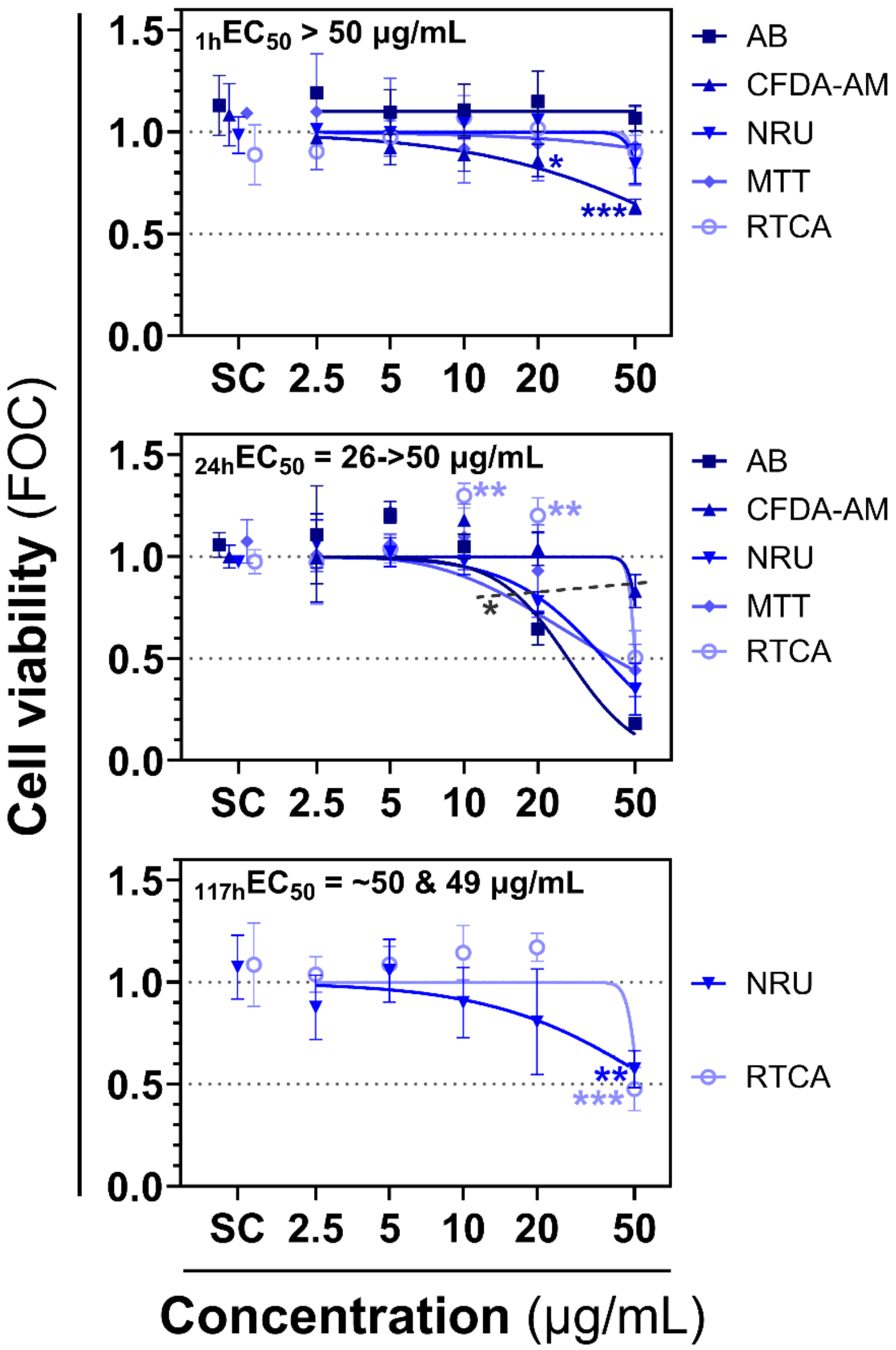

Supplementary Figure S7: **The impact of the organochlorine mixture (OC-MIX) on Sertoli cell TM4 viability determined by using a variety of conventional assays, including alamarBlue (AB), CFDA-AM, neutral red (NR), and MTT assays at 1 h, 24 h, and 117 h**. The mean and standard deviation (SD) are shown from a minimum of 3 independent experiments. Significant deviations from the solvent control (SC, DMSO 0.1%) are denoted by an asterisk (*). Statistical significance was determined using one-way ANOVA followed by Dunnett's test (*p≤0.05, **p≤0.01, ***p≤0.001).

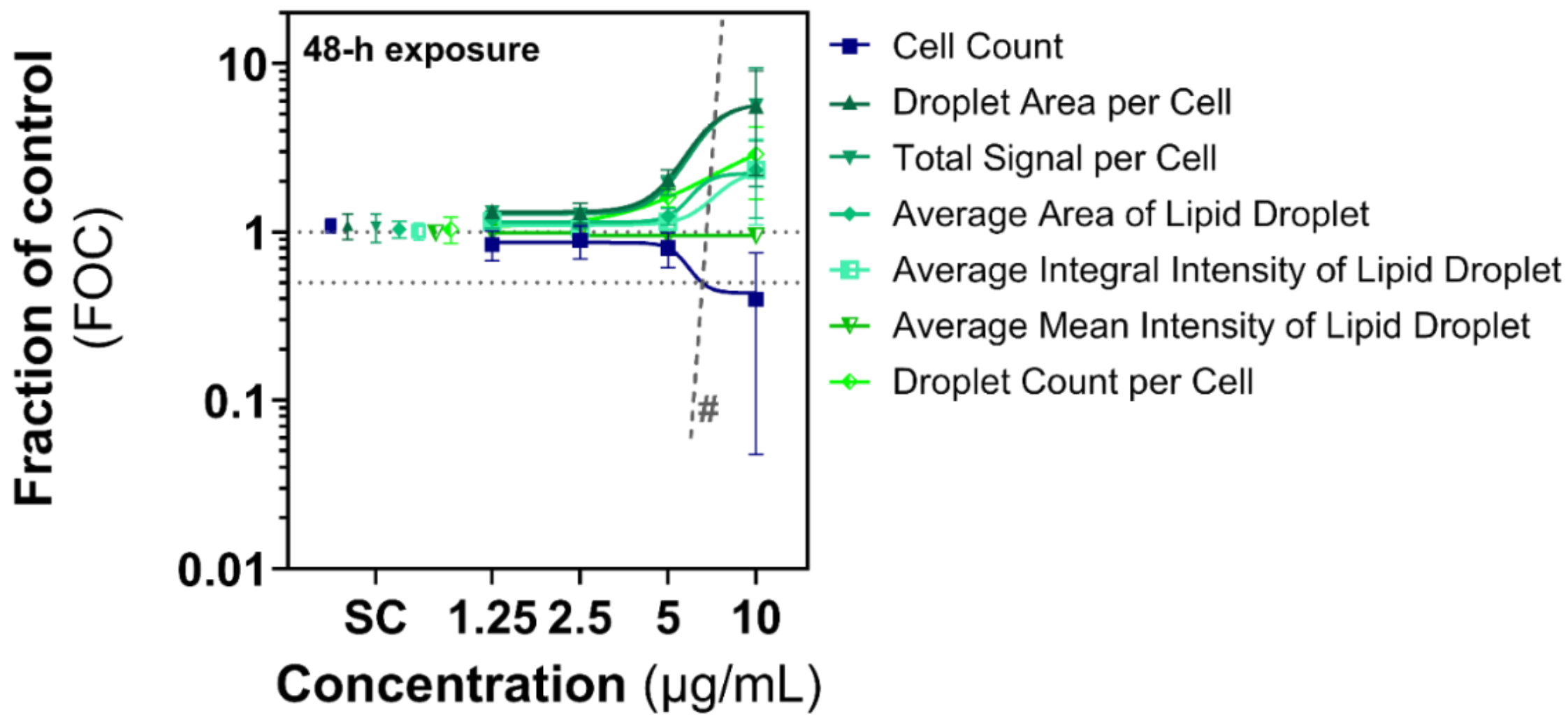

Supplementary Figure S8: Concentration-dependent lipid accumulation induced by amiodarone in Sertoli TM4 cells after 48-h exposures. Lipid accumulation was evaluated by staining neutral lipids with BODIPY 493/503 (green) and co-staining with DAPI (blue). Quantitative analysis of lipid accumulation and cell numbers after 48-h exposure to amiodarone (ranging from 1.25 to 10 µM). The mean and standard deviation (SD) are based on at least three independent experiments (n=3-6). Statistical significance from the solvent control (SC, 0.1% v/v DMSO) was determined using Kruskal-Wallis analysis followed by Dunn's test (#).

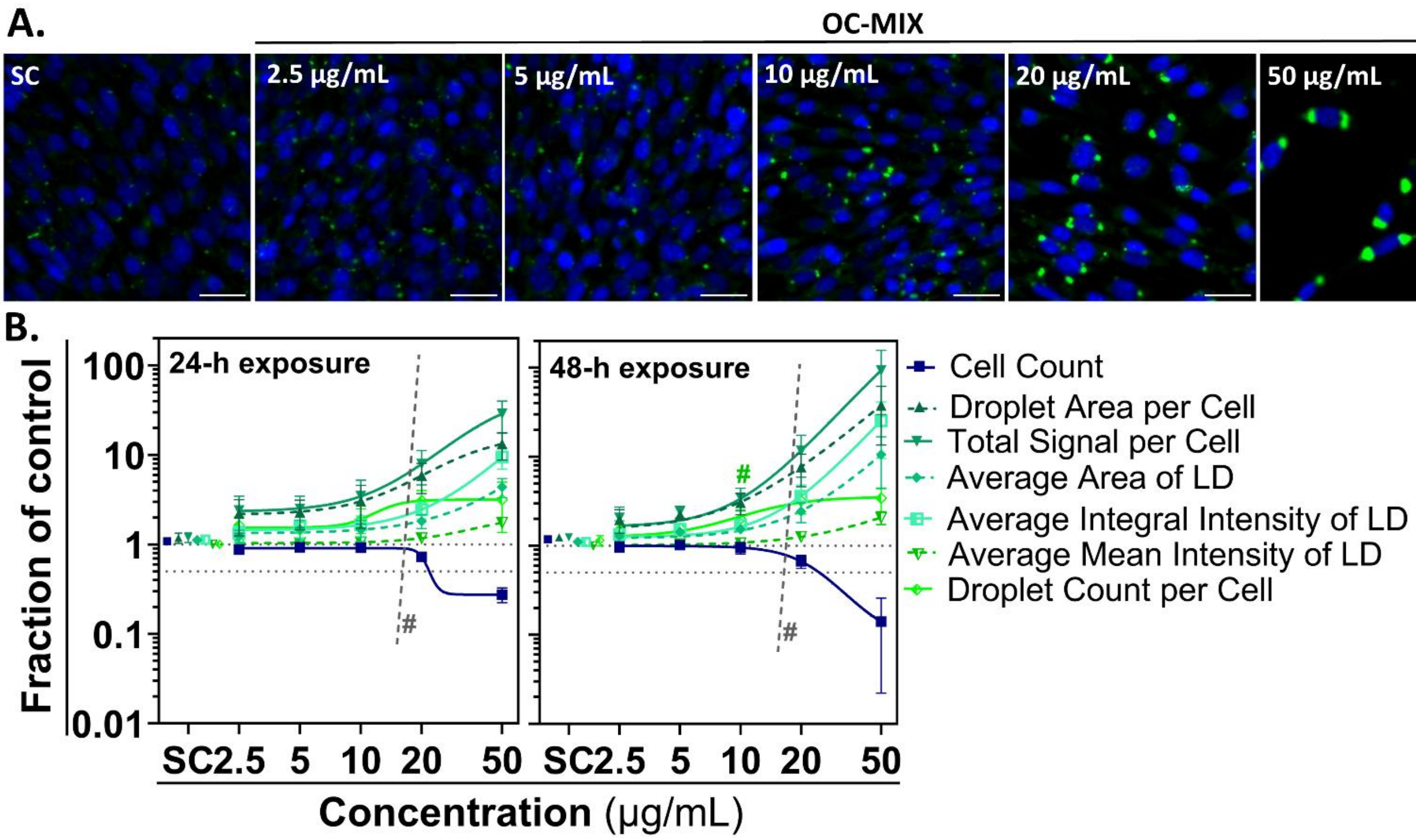

Supplementary Figure S9: Induction of lipid droplet (LD) accumulation by the organochlorine mixture OC-MIC in Sertoli TM4 cells after 24- and 48-h exposures. Lipid accumulation was evaluated by staining neutral lipids with BODIPY 493/503 (green) and co-staining with DAPI (blue). (**A.**) Representative images depicting the accumulation of neutral lipids in Sertoli TM4 cells treated with the solvent control (DMSO 0.1%, SC) and OC-MIX at concentrations of 2.5-50 µg/mL for 48 h. Scale bar = 30 µm. (**B.**) Quantitative analysis of lipid accumulation and cell numbers after 24- and 48-h exposures to OC-MIX. The mean and standard deviation (SD) are based on three independent experiments (n=3). Statistical significance from the solvent control (SC, 0.1% v/v DMSO) was determined using Kruskal-Wallis analysis followed by Dunnett's test (#).

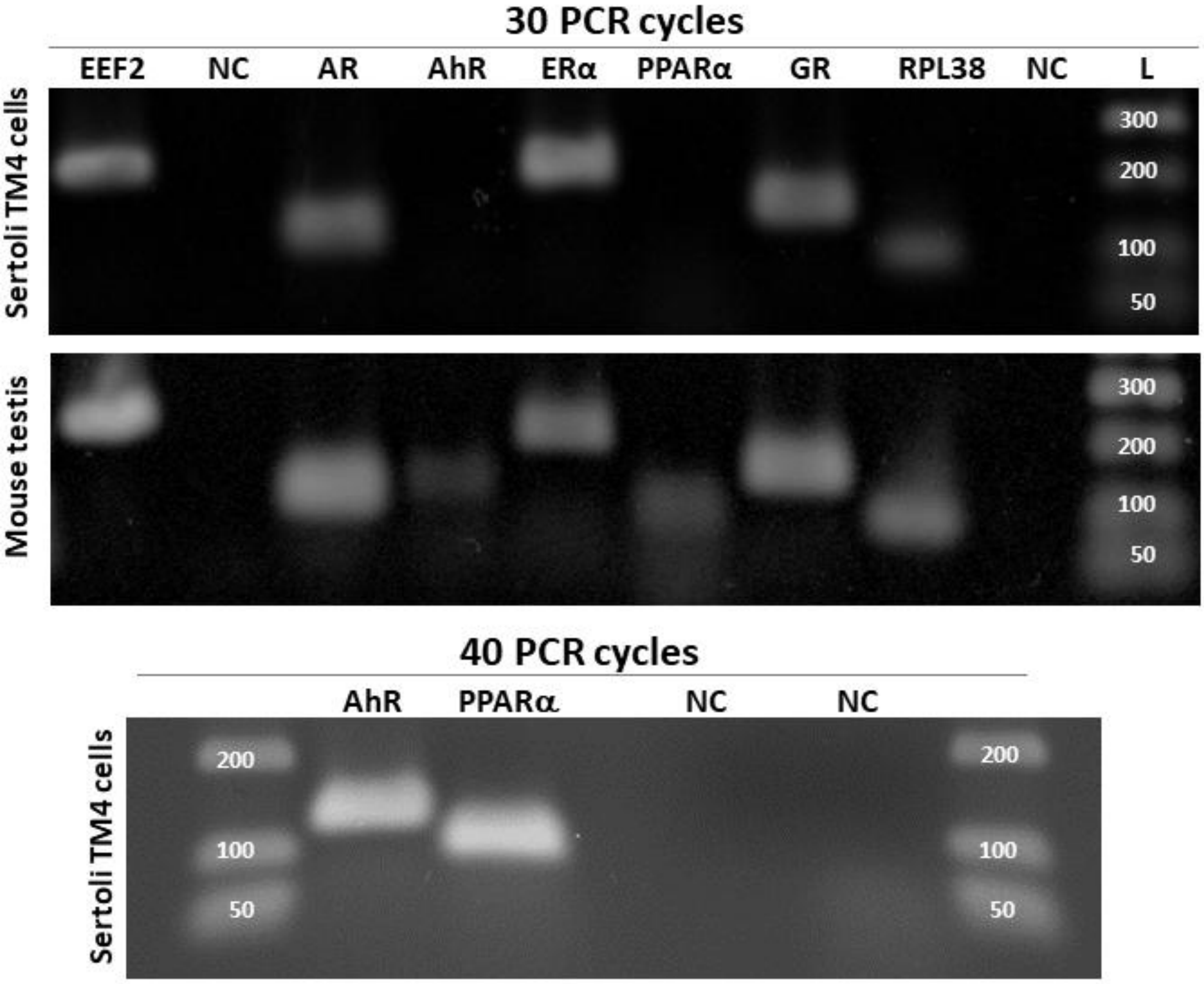

Supplementary Figure S10: **Expression of selected receptors in Sertoli TM4 cells and mouse adult testis at the mRNA level**. RT-PCR screening of mouse AhR (aryl hydrocarbon receptor), AR (androgen receptor), ERα (estrogen receptor α), GR (glucocorticoid receptor), PPARα (peroxisome proliferator-activated receptor α), and reference genes (EEF2 - eukaryotic elongation factor 2, RPL38 - ribosomal protein L38) in Sertoli TM4 cells and adult mice testis. Representative images of RT-PCR analyses of independent samples and mRNA isolations. NC, negative control (without cDNA).

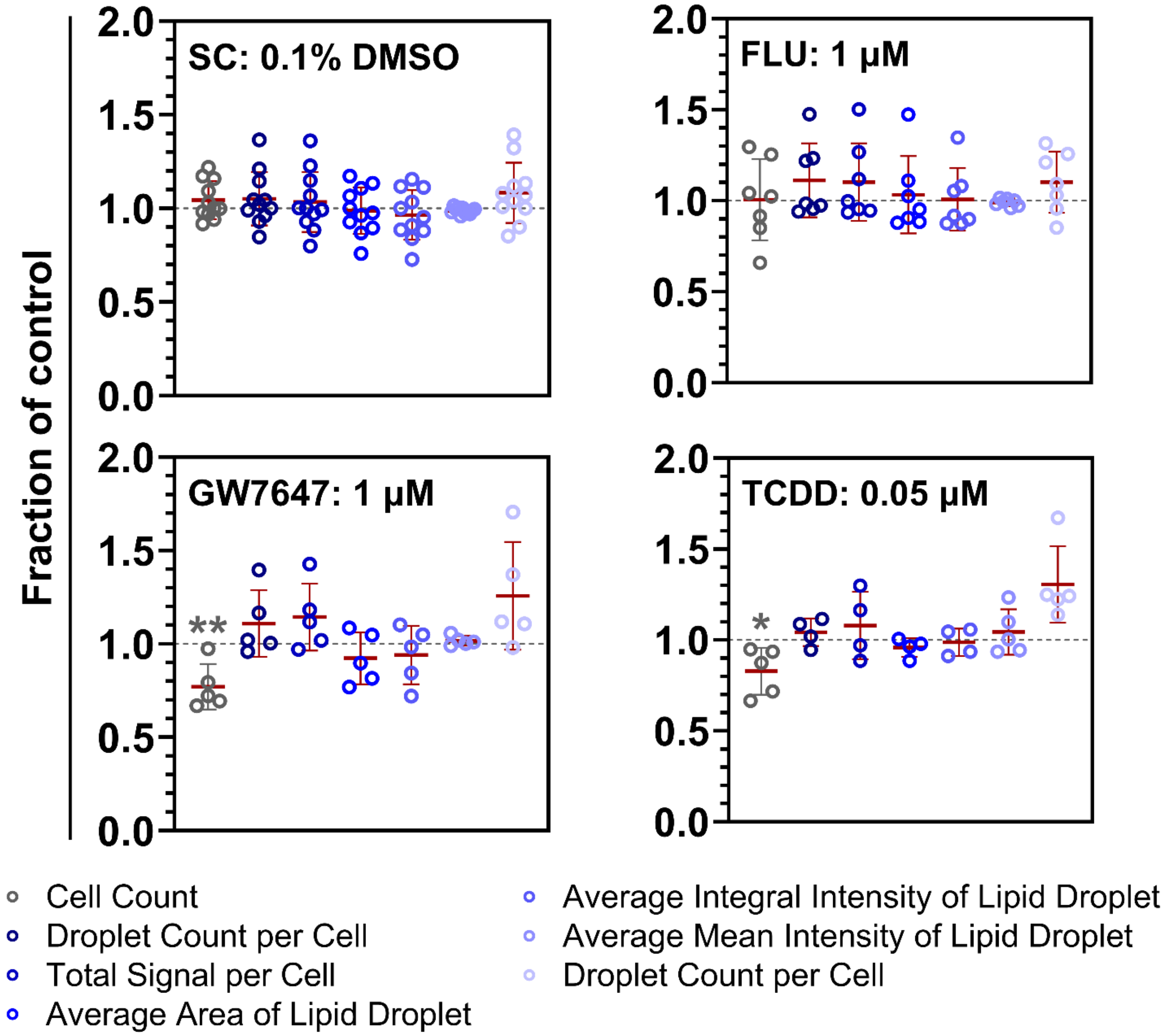

Supplementary Figure S11: **No significant neutral lipid accumulation in Sertoli TM4 cells after 48-h exposure to androgen receptor antagonist flutamide (1 µM), peroxisome proliferator-activated receptor alpha PPARα agonist GW76647 (1 µM), and aryl hydrocarbon receptor agonist TCDD (0.05 µM).** Neutral lipid accumulation was detected using BODIPY 493/503 staining, while cells were co-stained with DAPI. The figures display the mean and standard deviation (SD) from at least four independent experiments (n = 4-7), along with individual data points. Statistical analysis was performed using one-way ANOVA or Kruskal-Wallis ANOVA on ranks. Asterisks (**p<0.01, *p≤0.05; one-way ANOVA followed by Dunnett's test) indicate changes relative to the solvent control (**SC**, 0.1% v/v DMSO). **TCDD**; 2,3,7,8-tetrachlorodibenzodioxin.

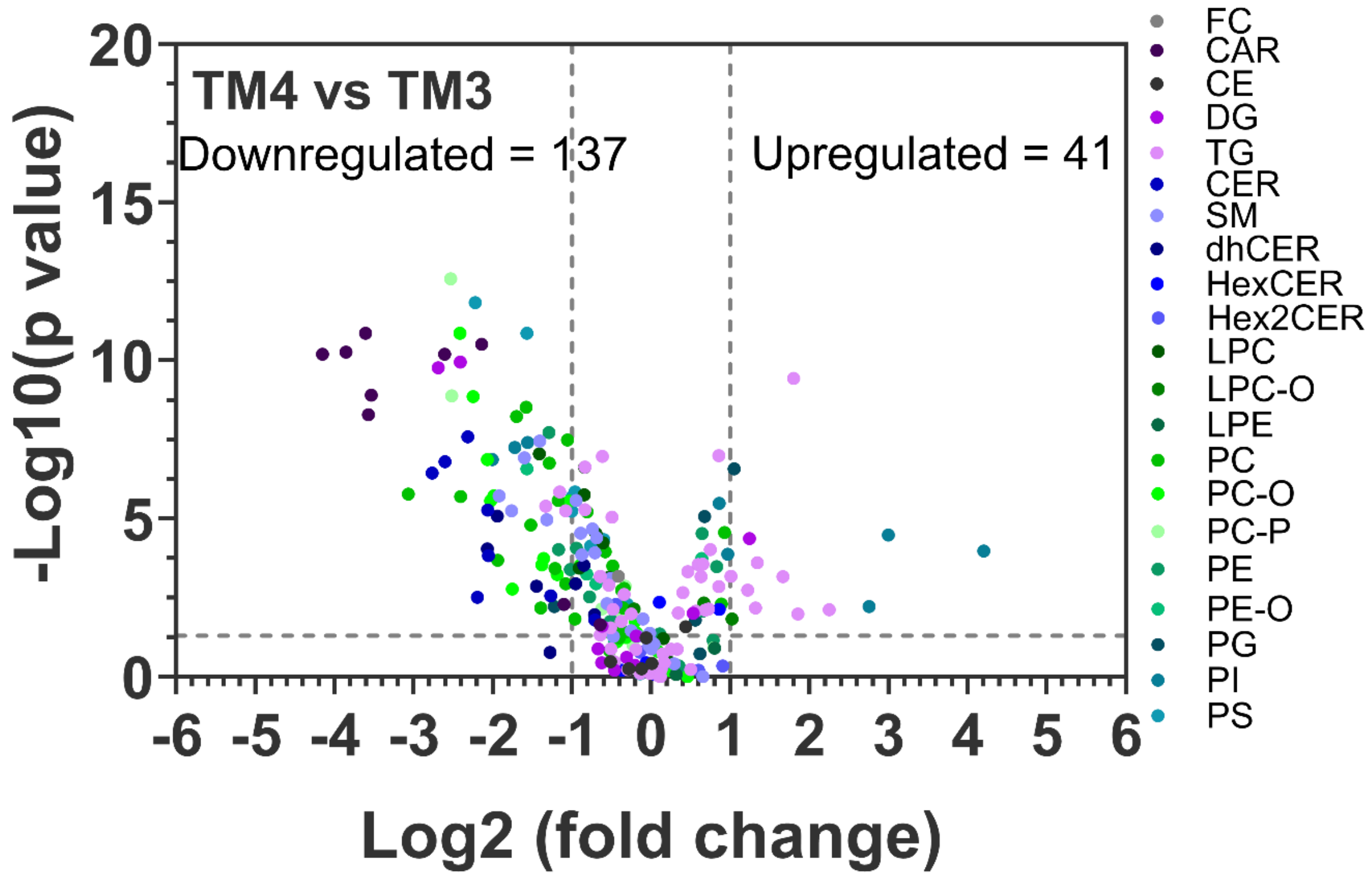

Supplementary Figure S12: Targeted lipidomic analysis of Sertoli TM4 cells compared to Leydig TM3 cells, focusing on individual lipid species levels. A volcano plot shows Log2(fold change) (Sertoli TM4 vs. Leydig TM3 cells) on the x-axis and -Log10(false discovery rate)-corrected p-values on the y-axis. A horizontal line indicates a -Log10(p-value) of 1.3, indicating a significance level of p = 0.05. Vertical lines emphasize Log2(fold change) values of -1 and +1 (fold change values of 0.5 and 2). Each data point represents a distinct lipid species within various subclasses, including free cholesterol (**FC**), acylcarnitines (**CAR**), , cholesteryl esters (**CE**), diacylglycerols (**DG**), triacylglycerols (**TG**), ceramides (**CER**), sphingomyelins (**SM**), dihydroceramides (**dhCER**), , hexosylceramides (**HexCER**), dihexosylceramides (**Hex2CER**), lysophosphatidylcholine (**LPC**), alkyl ether-linked lysophosphatidylcholines (**LPC-O**), lysophosphatidylethanolamines (**LPE**), phosphatidylcholines (**PC**), alkylphosphatidylcholines (**PC-O**), alkenylphosphatidylcholines (**PC-P**), , phosphatidylethanolamines (**PE**), ether-linked phosphatidylethanolamine (**PE-O**), phosphatidylglycerol (**PG**), phosphatidylinositol (**PI**), phosphatidylserine (**PS**).

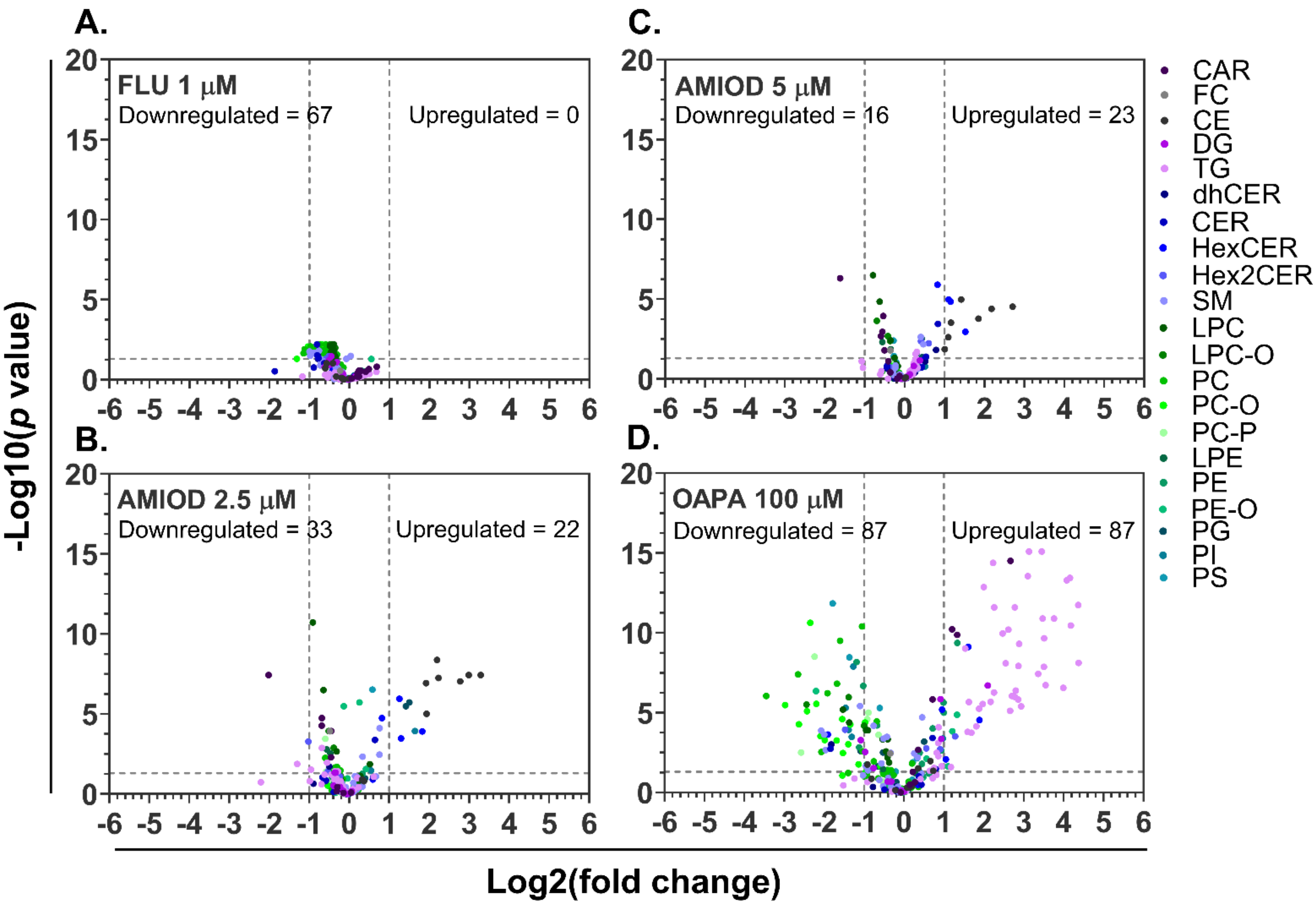

Supplementary Figure S13: Targeted lipidomic analysis of Sertoli TM4 cells exposed to flutamide (A.) and positive controls: amiodarone (2.5 µM (B.) and 5 µM (C.) and the mixture of oleic and palmitic acid (OAPA, 2:1 ratio, 100 µM) (D.) for 48 h, focusing on individual lipid species levels. Volcano plots show Log2(fold change) (treatment vs. solvent control – 0.1% DMSO) on the x-axis and -Log10(false discovery rate)-corrected p-values on the y-axis. A horizontal line indicates a -Log10(p-value) of 1.3, indicating a significance level of p = 0.05. Vertical lines emphasize Log2(fold change) values of -1 and +1 (fold change values of 0.5 and 2). Each data point represents a distinct lipid species within various subclasses, including acylcarnitines (**CAR**), free cholesterol (**FC**), cholesteryl esters (**CE**), diacylglycerols (**DG**), triacylglycerols (**TG**), dihydroceramides (**dhCER**), ceramides (**CER**), hexosylceramides (**HexCER**), dihexosylceramides (**Hex2CER**), and sphingomyelins (**SM**), lysophosphatidylcholine (**LPC**), alkyl ether-linked lysophosphatidylcholines (**LPC-O**), phosphatidylcholines (**PC**), alkylphosphatidylcholines (**PC-O**), alkenylphosphatidylcholines (**PC-P**), lysophosphatidylethanolamines (**LPE**), phosphatidylethanolamines (**PE**), ether-linked phosphatidylethanolamine (**PE-O**), phosphatidylglycerol (**PG**), phosphatidylinositol (**PI**), phosphatidylserine (**PS**).

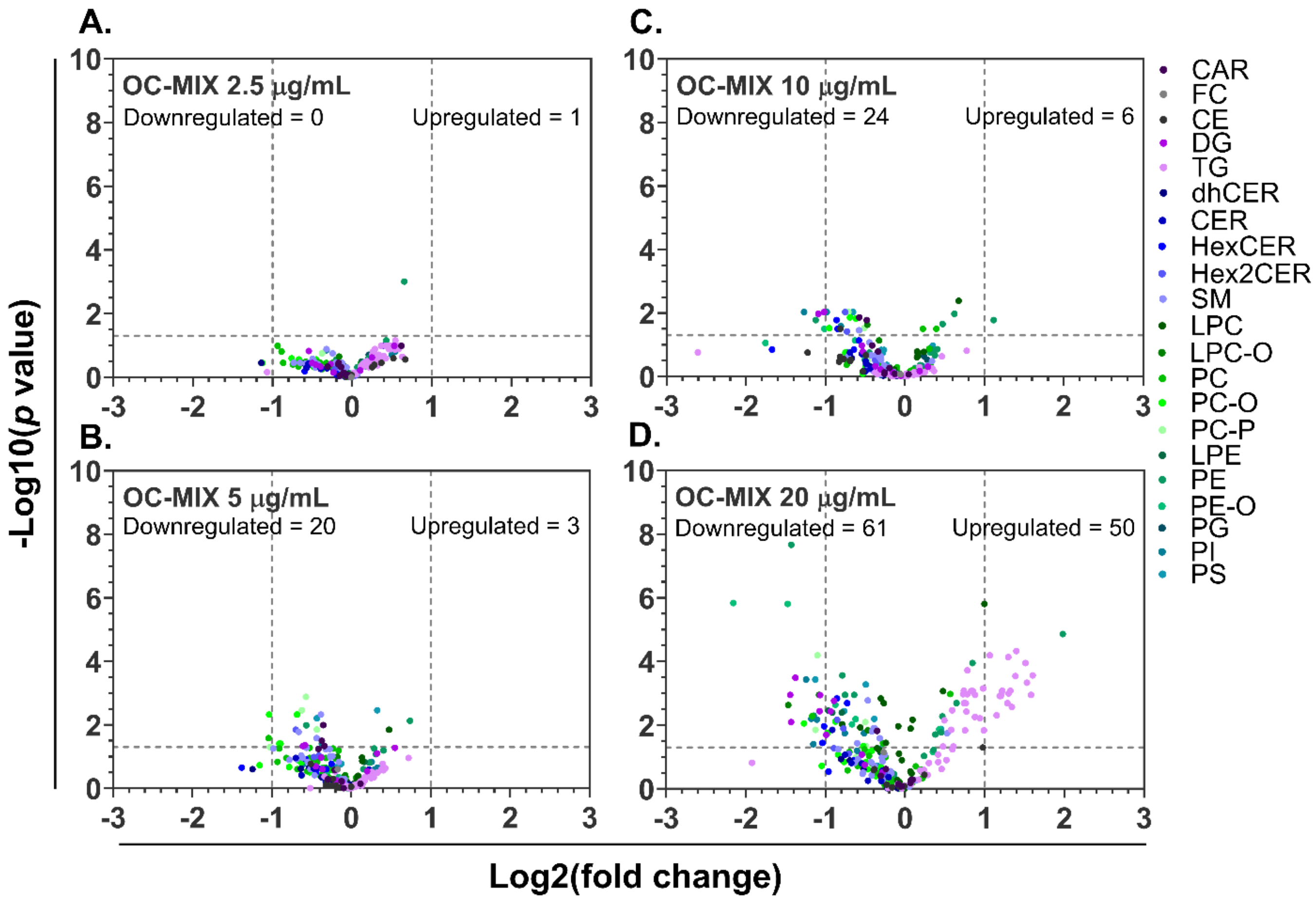

Supplementary Figure S14: Targeted lipidomic analysis of Sertoli TM4 cells exposed to the organochlorine mixture OC-MIX for 48 h, focusing on individual lipid species levels at 2.5 (A.), 5 (B.), 10 (C.), and 20 µg/mL (D.). Volcano plots show Log2(fold change) (OC-MIX vs. solvent control – 0.1% DMSO) on the x-axis and -Log10(false discovery rate)-corrected p-values on the y-axis. A horizontal line indicates a -Log10(p-value) of 1.3, indicating a significance level of p = 0.05. Vertical lines emphasize Log2(fold change) values of -1 and +1 (fold change values of 0.5 and 2). Each data point represents a distinct lipid species within various subclasses, including acylcarnitines (**CAR**), free cholesterol (**FC**), cholesteryl esters (**CE**), diacylglycerols (**DG**), triacylglycerols (**TG**), dihydroceramides (**dhCER**), ceramides (**CER**), hexosylceramides (**HexCER**), dihexosylceramides (**Hex2CER**), and sphingomyelins (**SM**), lysophosphatidylcholine (**LPC**), alkyl ether-linked lysophosphatidylcholines (**LPC-O**), phosphatidylcholines (**PC**), alkylphosphatidylcholines (**PC-O**), alkenylphosphatidylcholines (**PC-P**), lysophosphatidylethanolamines (**LPE**), phosphatidylethanolamines (**PE**), ether-linked phosphatidylethanolamine (**PE-O**), phosphatidylglycerol (**PG**), phosphatidylinositol (**PI**), phosphatidylserine (**PS**).

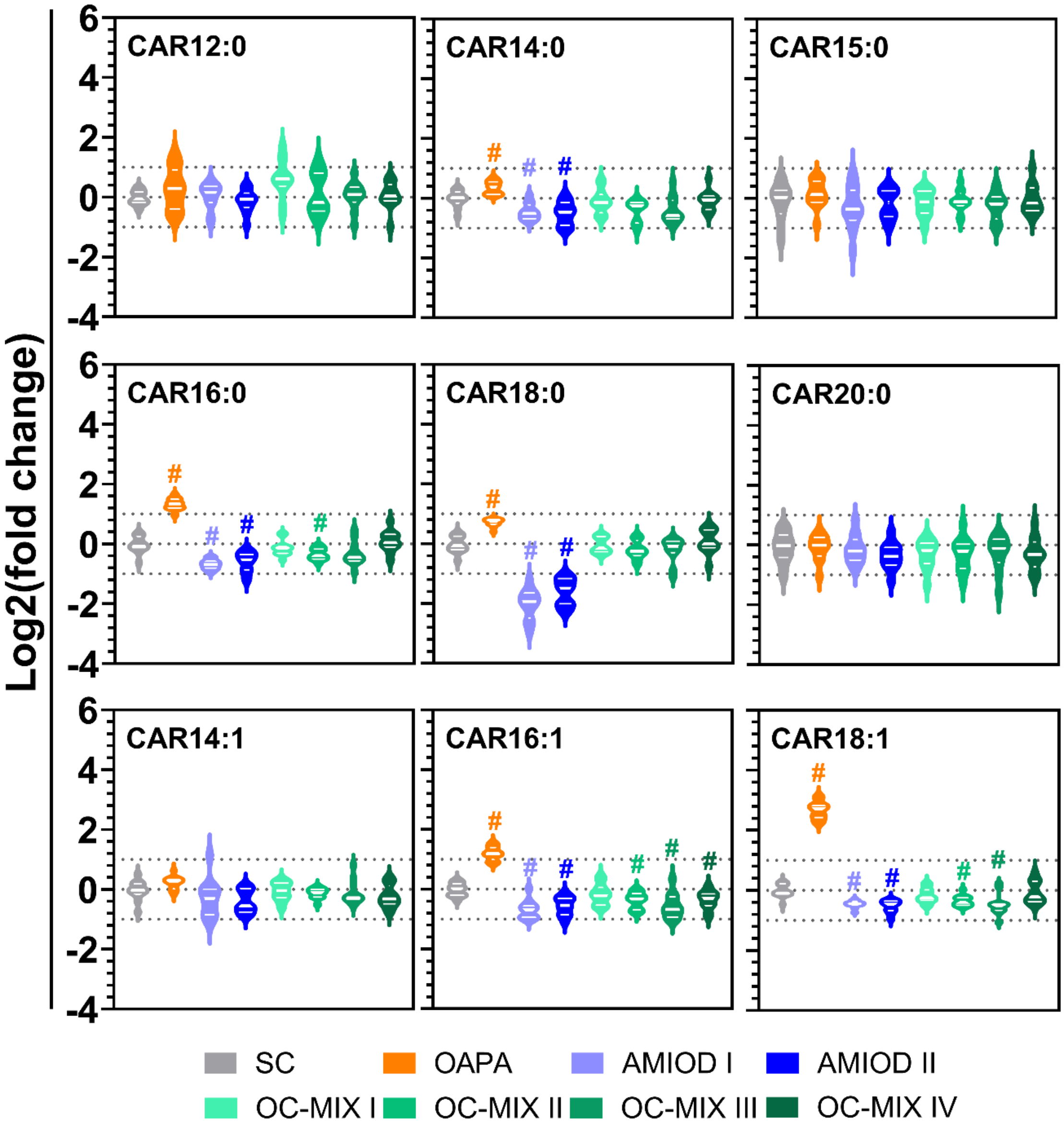

Supplementary Figure S15: Changes in individual acylcarnitines (CAR) in Sertoli TM4 cells after 48-h exposure to the OC-MIX and control compounds. Treatments: OC-MIX (I: 2.5, II: 5, III: 10, IV: 20 µg/mL), OAPA (oleic/palmitic acid mixture at a 2:1 ratio, 100 µM), AMIOD (amiodarone, I: 2.5, II: 5 µM), and solvent control (SC, 0.1% DMSO). Data are shown as $\log_2$(fold-change) relative to SC. Violin plots display the first, median (white line), and third quartiles. # indicates statistical significance vs. SC ($p < 0.05$) (non-parametric t-test). Experiments were conducted three times independently in quadruplicate ($n = 12$).

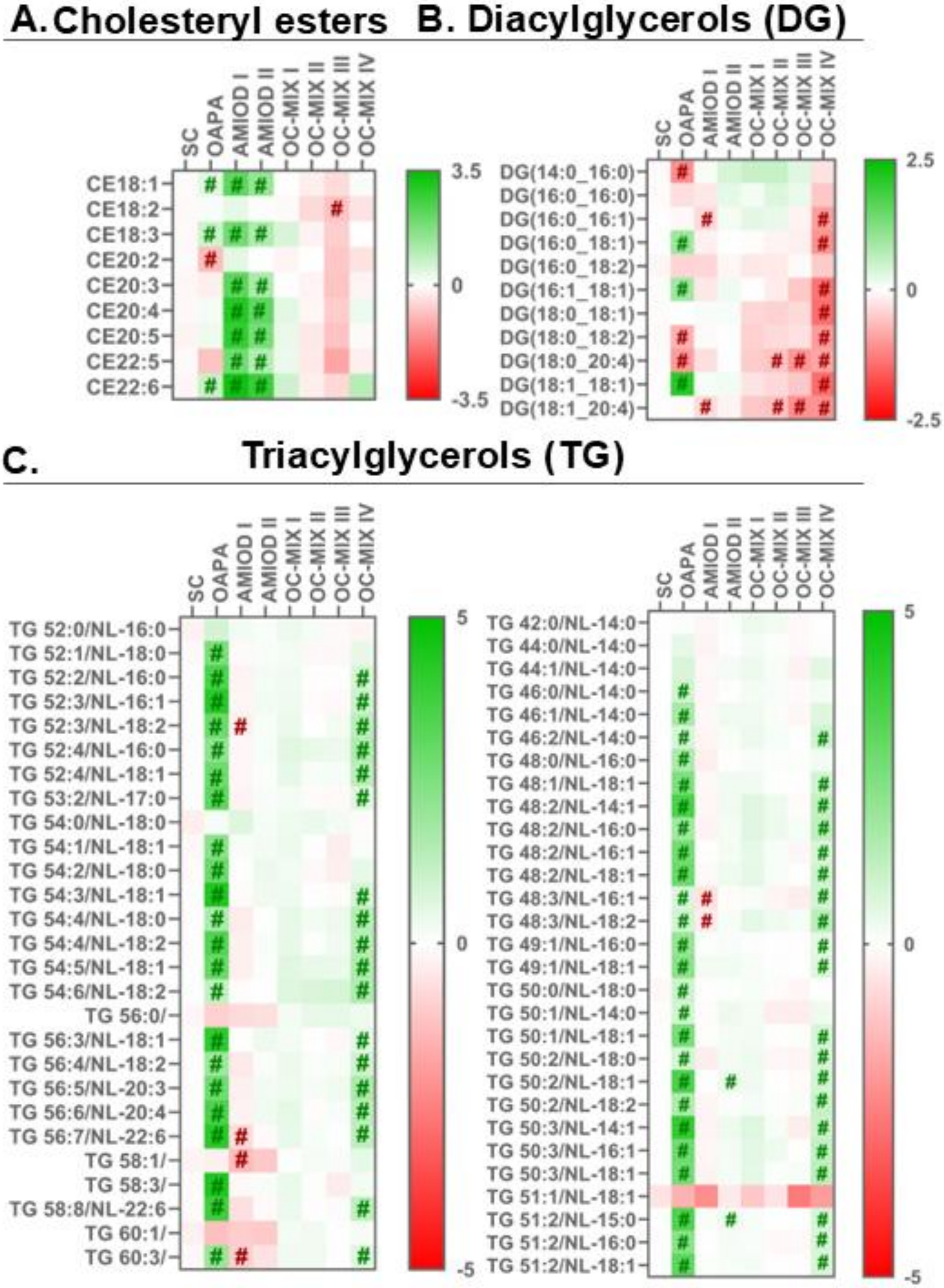

Supplementary Figure S16: Impact of the organochlorine mixture OC-MIX along with negative and positive (OAPA, AMIOD) controls on individual neutral lipid species of cholesteryl esters (CE, A.), diacylglycerols (DG, B.), and triacylglycerols (TG, B.) — in Sertoli TM4 cells following 48-h exposure. Heatmaps showcasing alterations in lipid profiles of cells exposed to various compounds, a combination of oleic acid and palmitic acid in a 2:1 ratio (**OAPA**, 100 µM), amiodarone (**AMIOD**; **I**: 2.5 µM, **II**: 5 µM), and **OC-MIX** (**I**: 2.5 µg/mL, **II**: 5 µg/mL, **III**: 10 µg/mL, **IV**: 20 µg/mL) (columns). Results are presented as the mean of Log2(fold-change) (treatment *vs*. solvent control, SC) for individual lipid species (rows). The fold changes are color-coded by green (upregulation), red (downregulation), and white (no change). Symbols (#) indicate statistical significance ($p < 0.05$) from SC (non-parametric t-test). The experiments were conducted three times independently in quadruplicate (n = 12).

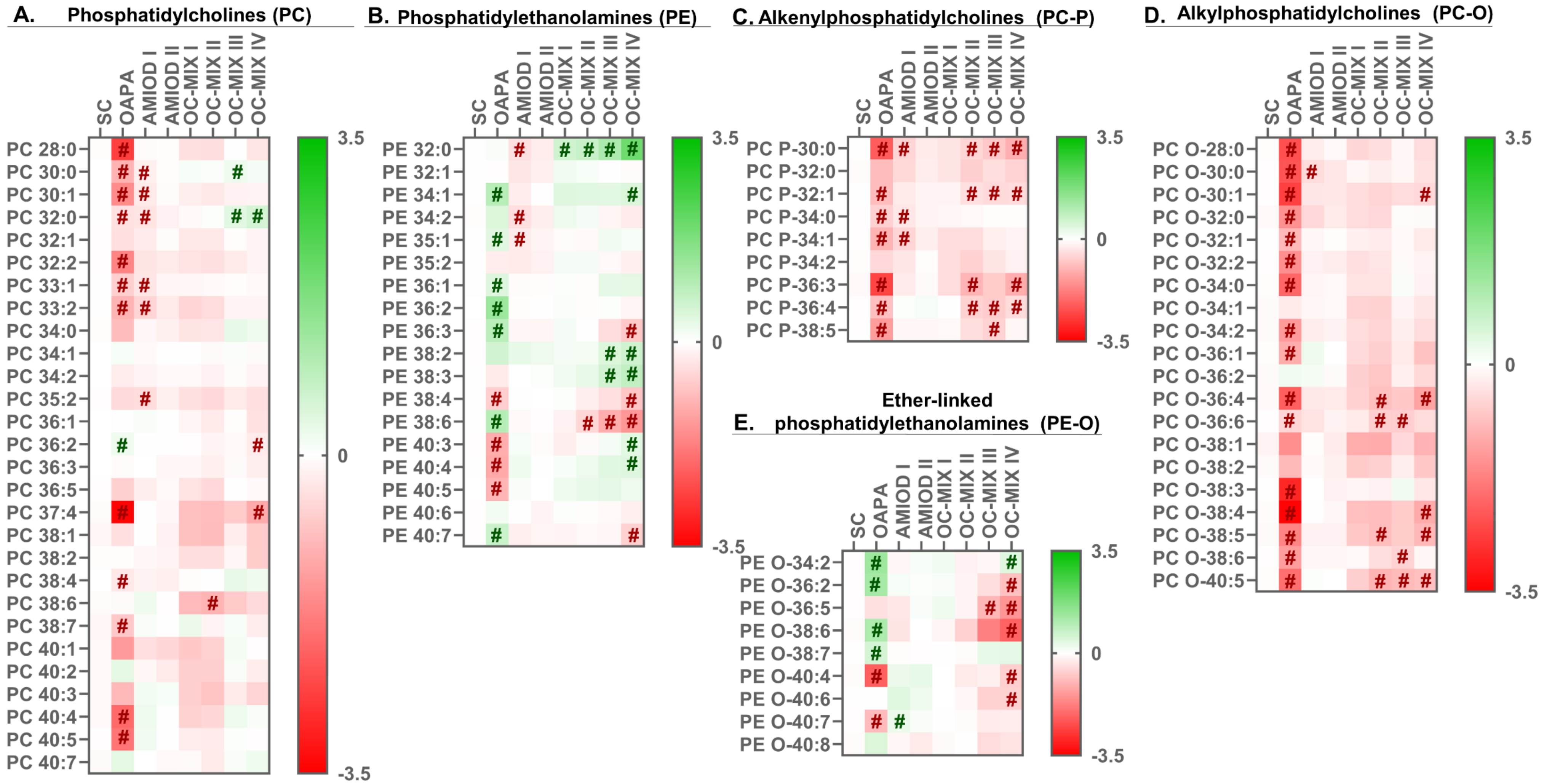

Supplementary Figure S17: Impact of the organochlorine mixture OC-MIX along with negative and positive (OAPA, AMIOD) controls on individual lipid species of lipid subclasses — phosphatidylcholines (PCs, A.), phosphatidylethanolamines (PEs, B.), alkenylphosphatidylcholines (PC-P, C.), alkyl ether-linked phosphatidylcholines (PC-O, D.), and alkyl ether-linked phosphatidylethanolamines (PE-O, E.) — in Sertoli TM4 cells following 48-h exposure. Heatmaps showcasing alterations in lipid profiles of cells exposed to various compounds, a combination of oleic acid and palmitic acid in a 2:1 ratio (**OAPA**, 100 µM), amiodarone (**AMIOD**; I: 2.5 µM, II: 5 µM), and **OC-MIX** (**I**: 2.5 µg/mL, **II**: 5 µg/mL, **III**: 10 µg/mL, **IV**: 20 µg/mL) (columns). Results are presented as the mean of Log2(fold-change) (treatment *vs*. solvent control, SC) for individual lipid species (rows). The fold changes are color-coded by green (upregulation), red (downregulation), and white (no change). Symbols (#) indicate statistical significance ($p < 0.05$) from SC (non-parametric t-test). The experiments were conducted three times independently in quadruplicate (n = 12).

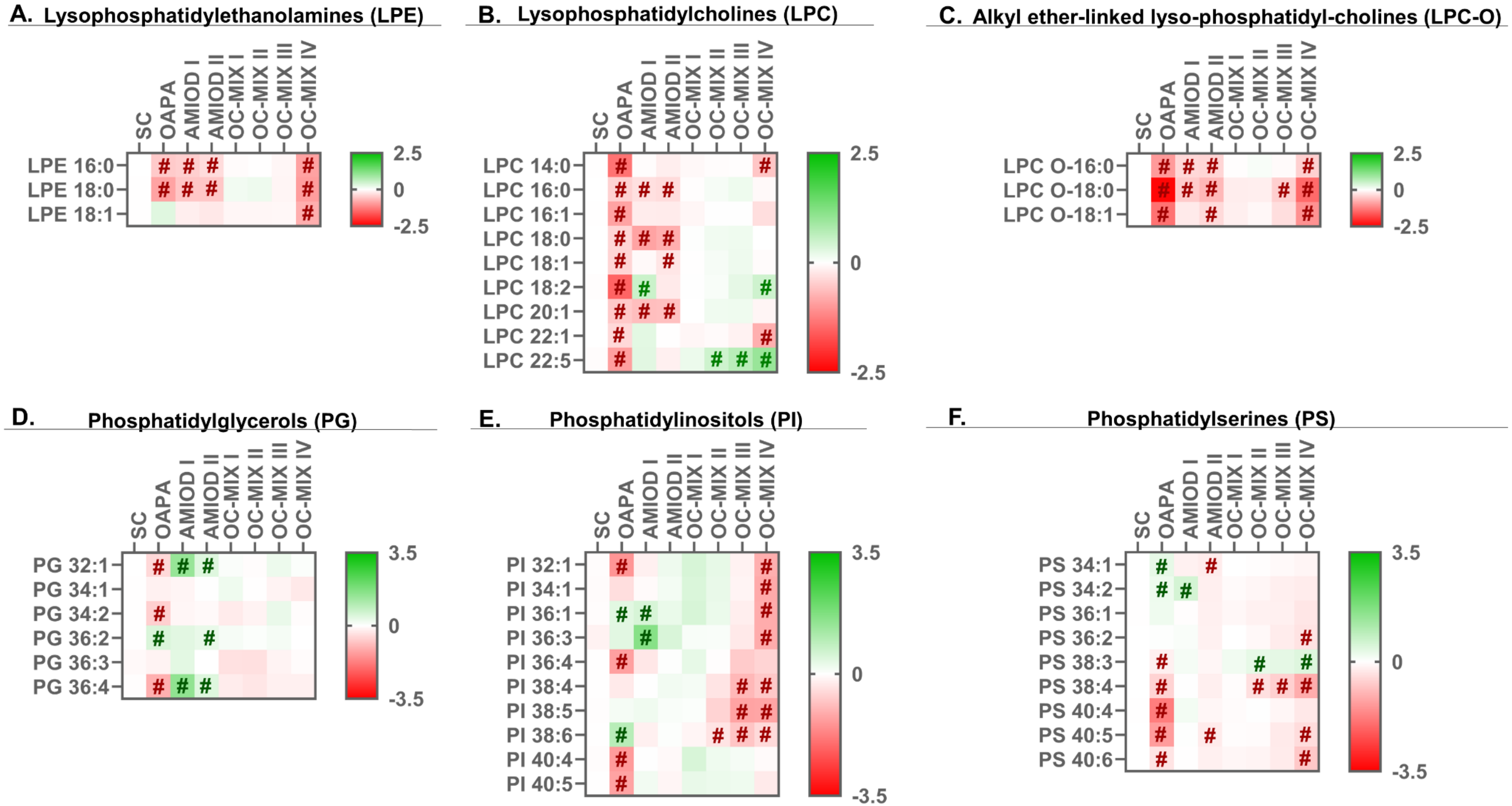

Supplementary Figure S18: Impact of the OC-MIX along with negative and positive (OAPA, AMIOD) controls on individual lipid species of lipid subclasses — lysophosphatidylethanolamines (LPEs, A.), lysophosphatidylcholines (LPCs, B.), alkyl ether-linked lysophosphatidylcholines (LPC-O, C.), phosphatidylglycerols (PG, D.), phosphatidylinositols (PIs, E.), and phosphatidylserines (PSs, E.) — in Sertoli TM4 cells following 48-h exposure. Heatmaps showcasing alterations in lipid profiles of cells exposed to various compounds, a combination of oleic acid and palmitic acid in a 2:1 ratio (**OAPA**, 100 µM), amiodarone (**AMIOD**; I: 2.5 µM, II: 5 µM), and **OC-MIX** (**I**: 2.5 µg/mL, **II**: 5 µg/mL, **III**: 10 µg/mL, **IV**: 20 µg/mL) (columns). Results are presented as the mean of Log2(fold-change) (treatment *vs*. solvent control, SC) for individual lipid species (rows). The fold changes are colored-coded by green (upregulation), red (downregulation), and white (no change). Symbols (#) indicate statistical significance ($p < 0.05$) from SC (non-parametric t-test). The experiments were conducted three times independently in quadruplicate (n = 12).

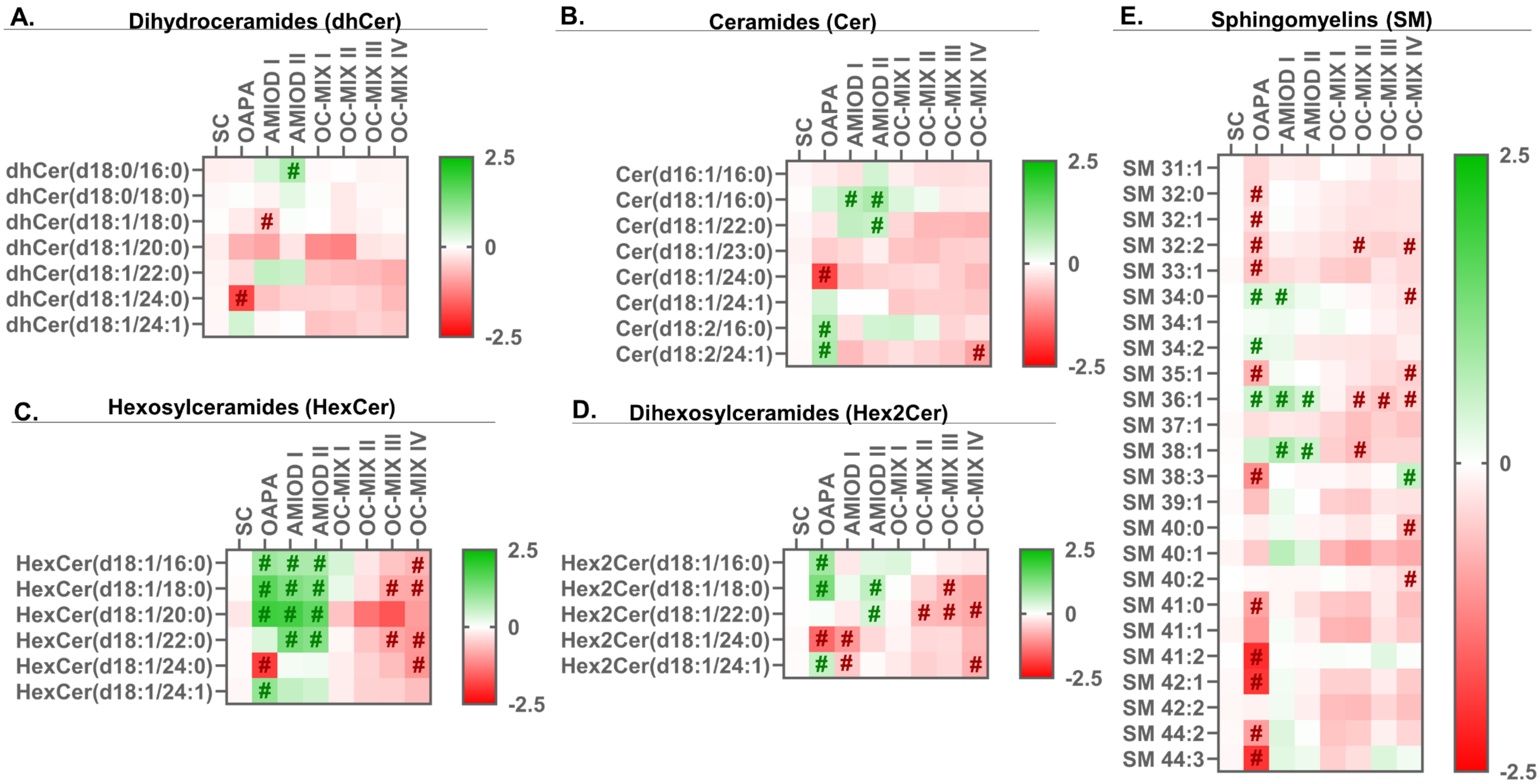

Supplementary Figure S19: Impact of the OC-MIX along with negative and positive (OAPA, AMIOD) controls on individual lipid species of lipid subclasses — dihydroceramides(dhCers, A.), ceramides (Cers, B.), hexosylceramides (HexCers, C.), dihexosylceramides (Hex2Cers, D.), and sphingomyelins (SMs, E.) — in Sertoli TM4 cells following 48-h exposure. Heatmaps showcasing alterations in lipid profiles of cells exposed to various compounds, a combination of oleic acid and palmitic acid in a 2:1 ratio (**OAPA**, 100 µM), amiodarone (**AMIOD**; **I**: 2.5 µM, **II**: 5 µM), and **OC-MIX** (**I**: 2.5 µg/mL, **II**: 5 µg/mL, **III**: 10 µg/mL, **IV**: 20 µg/mL) (columns). Results are presented as the mean of Log2(fold-change) (treatment *vs*. solvent control, SC) for individual lipid species (rows). The fold changes are color-coded by green (upregulation), red (downregulation), and white (no change). Symbols (#) indicate statistical significance ($p < 0.05$) from SC (non-parametric t-test). The experiments were conducted three times independently in quadruplicate (n = 12).

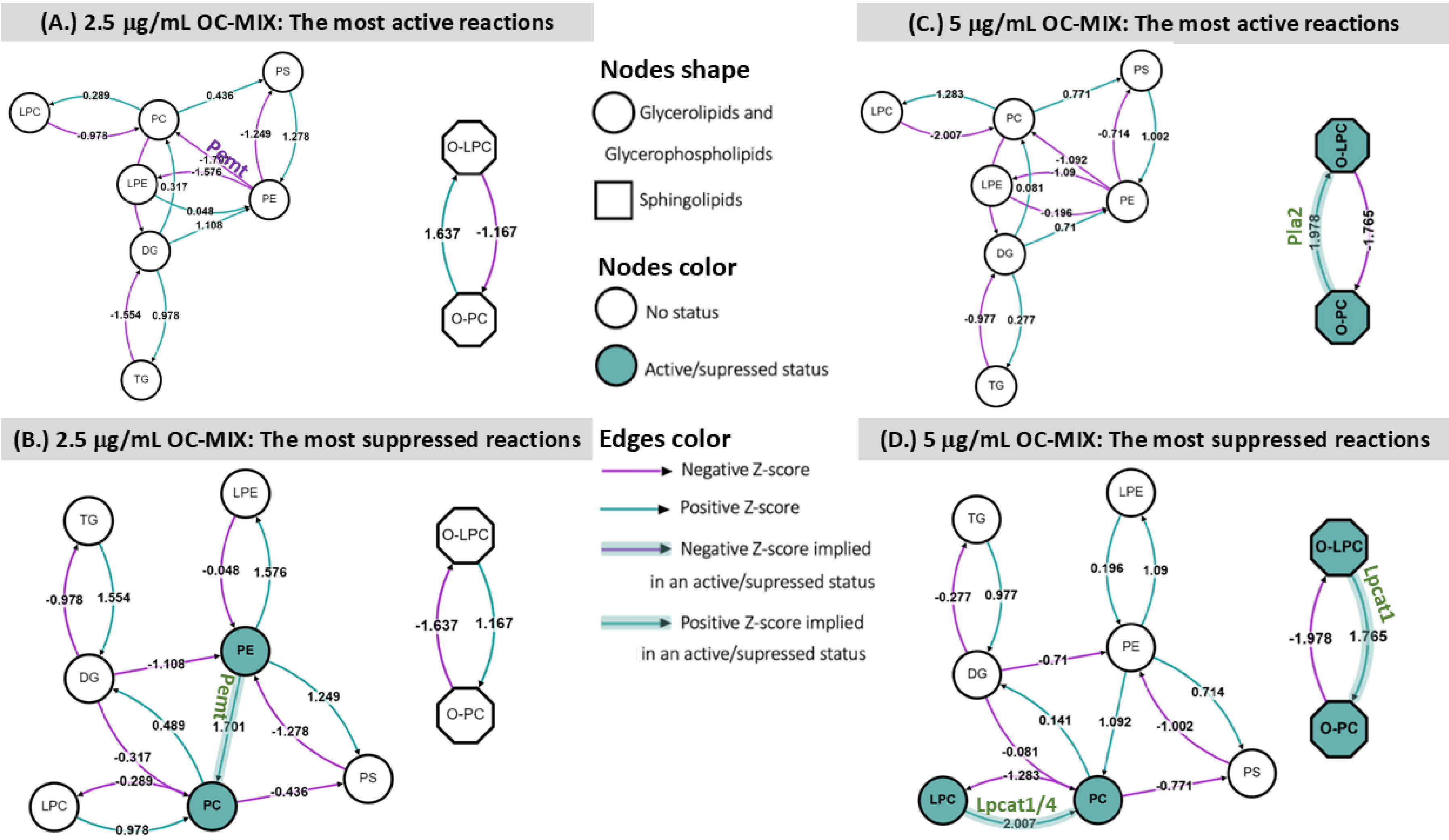

Supplementary Figure S20: Lipid pathway enrichment analysis in Sertoli TM4 cells treated with the organochlorine mixture (OC-MIX, 2.5 and 5 µg/mL) following 48-h exposure. BioPAN tool was used to identify (**A**, **C**) the most active reactions and (**B**, **D**) the most suppressed reactions by comparing OC-MIX-treated Sertoli TM4 cells (2.5 µg/mL: **A**, **B**; 5 µg/mL: **C**, **D**) with the solvent control (DMSO, 0.1%) and to suggest enzymes involved in these reactions. The most significantly dysregulated reactions occurred among lipid classes of phosphatidylethanolamines (**PE**), phosphatidylcholines (**PC**), lysophosphatidylcholine (**LPC**), alkyl ether-linked lysophosphatidylcholines (**LPC-O**), and alkylphosphatidylcholines (**PC-O**). **Lpcat1/4**, lysophosphatidylcholine acyltransferase 1/4; **Pemt**, phosphatidylethanolamine N-methyltransferase; **Pla2**, phospholipase A2. The default BioPAN settings were applied, with a significance threshold of $p \leq 0.05$. Z-scores indicate the direction and magnitude of lipid pathway changes: positive values reflect activation, while negative values indicate suppression, helping to pinpoint key enzymatic reactions affected by chemical exposure.

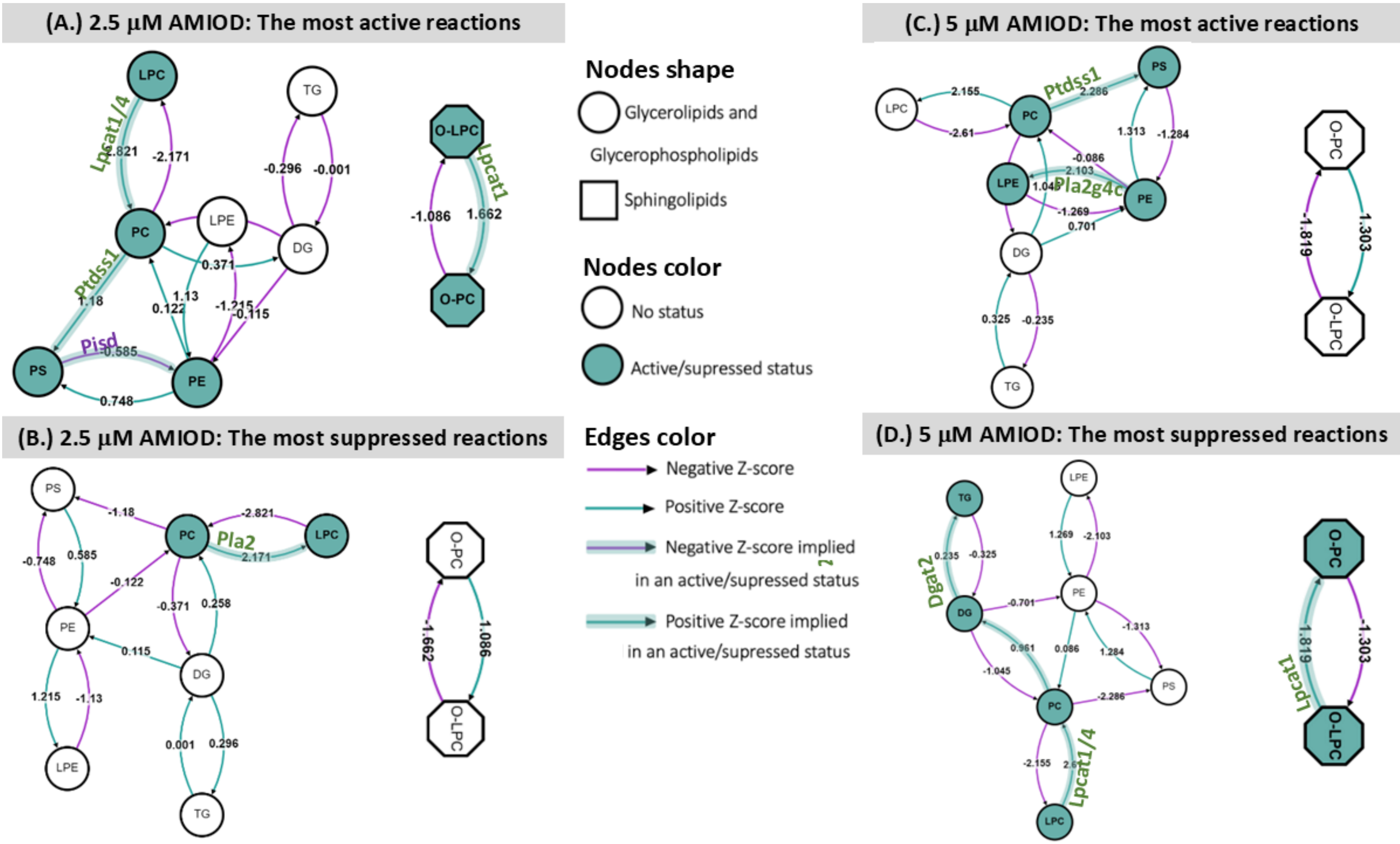

Supplementary Figure S21: Lipid pathway enrichment analysis in Sertoli TM4 cells treated with amiodarone (AMIOD, 2.5 and 5 µM) following 48-h exposure. BioPAN tool was used to identify (**A**, **C**) the most active reactions and (**B**, **D**) the most suppressed reactions by comparing AMIOD-treated Sertoli TM4 cells (2.5 µM: **A**, **B**; 5 µM: **C**, **D**) with the solvent control (DMSO, 0.1%) and to suggest enzymes involved in these reactions. The most significantly dysregulated reactions occurred among lipid classes of phosphatidylcholines (**PC**), diacylglycerides (**DG**), triacylglycerides (**TG**), lysophosphatidylethanolamines (**LPE**), lysophosphatidylcholine (**LPC**), phosphatidylethanolamines (**PE**), phosphatidylserines (**PS**), alkyl ether-linked lysophosphatidylcholines (**LPC-O**), and alkylphosphatidylcholines (**PC-O**). **Dgat1**, diacylglycerol O-acyltransferase; **Lpcat1/4**, lysophosphatidylcholine acyltransferase 1/4; **Pisd**, phosphatidylserine decarboxylase; **Pla2**, phospholipase A2; **Pla2g4c**, phospholipase A2, group IVC (cytosolic, calcium-independent; **Ptdss1**, phosphatidylserine synthase 1. The default BioPAN settings were applied, with a significance threshold of p ≤ 0.05. Z-scores indicate the direction and magnitude of lipid pathway changes: positive values reflect activation, while negative values indicate suppression, helping to pinpoint key enzymatic reactions affected by chemical exposure.

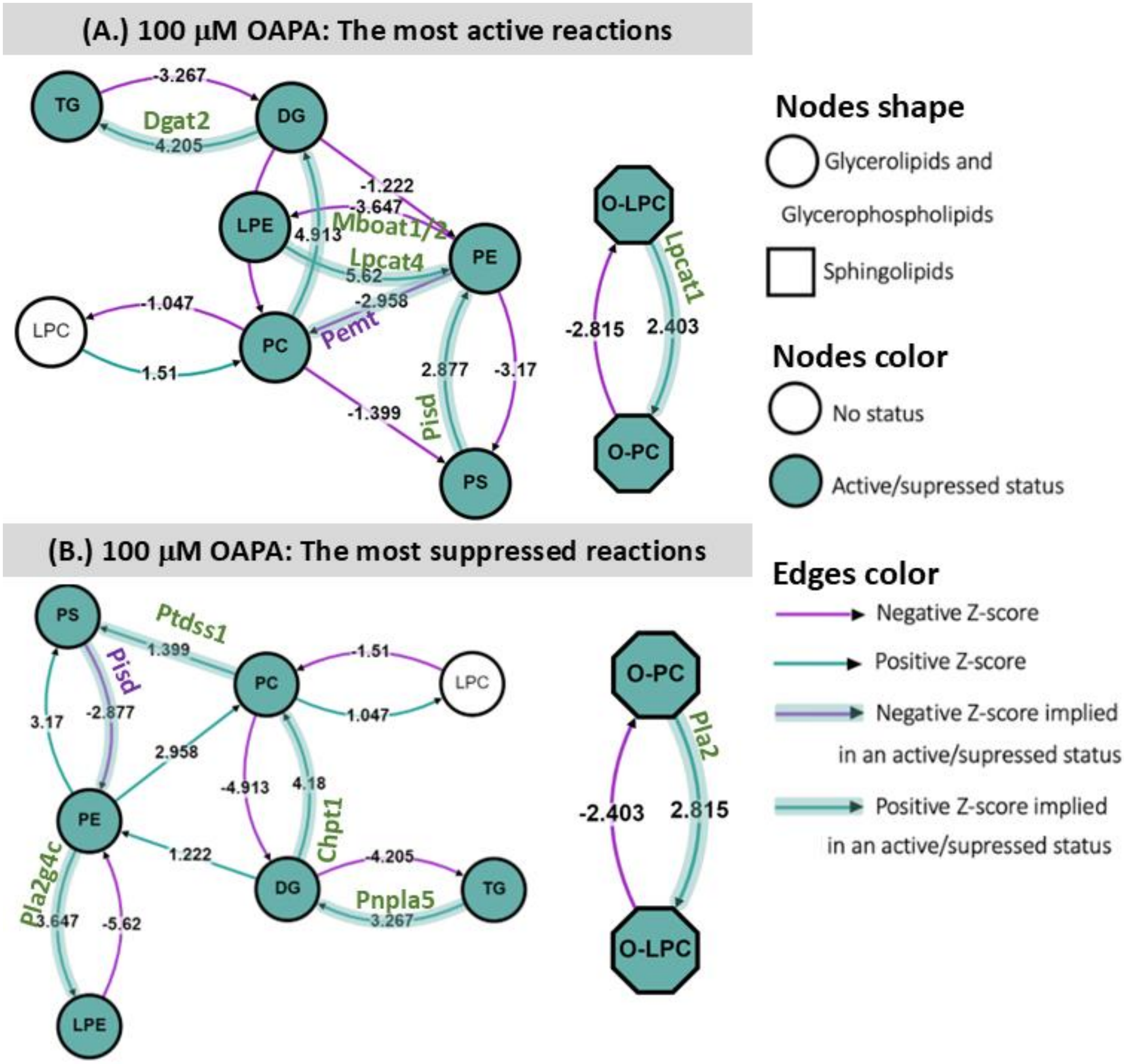

Supplementary Figure S22: Lipid pathway enrichment analysis in Sertoli TM4 cells treated with a mixture of fatty acids (OAPA) following 48-h exposure. OAPA: a combination of oleic acid and palmitic acid in a 2:1 ratio (100 µM). BioPAN tool was used to identify (**A**) the most active reactions and (**B**) the most suppressed reactions by comparing OAPA-treated Sertoli TM4 cells (100 µM, 48 h) with the solvent control (DMSO, 0.1%) and to suggest enzymes involved in these reactions. The most significantly dysregulated reactions occurred among lipid classes of phosphatidylcholines (**PC**), diacylglycerides (**DG**), triacylglycerides (**TG**), lysophosphatidylethanolamines (**LPE**), phosphatidylethanolamines (**PE**), phosphatidylserines (**PS**), alkyl ether-linked lysophosphatidylcholines (**LPC-O**), and alkylphosphatidylcholines (**PC-O**). **Chpt1**, choline phosphotransferase 1; **Dgat1**, diacylglycerol O-acyltransferase; **Lpcat1/4**, lysophosphatidylcholine acyltransferase 1/4; **Mboat1/2**, membrane-bound O-acyltransferase domain-containing 1/2; **Pisd**, phosphatidylserine decarboxylase; **Pla2**, phospholipase A2; **Pla2g4c**, phospholipase A2, group IVC (cytosolic, calcium-independent. The default BioPAN settings were applied, with a significance threshold of $p \leq 0.05$. Z-scores indicate the direction and magnitude of lipid pathway changes: positive values reflect activation, while negative values indicate suppression, helping to pinpoint key enzymatic reactions affected by chemical exposure.

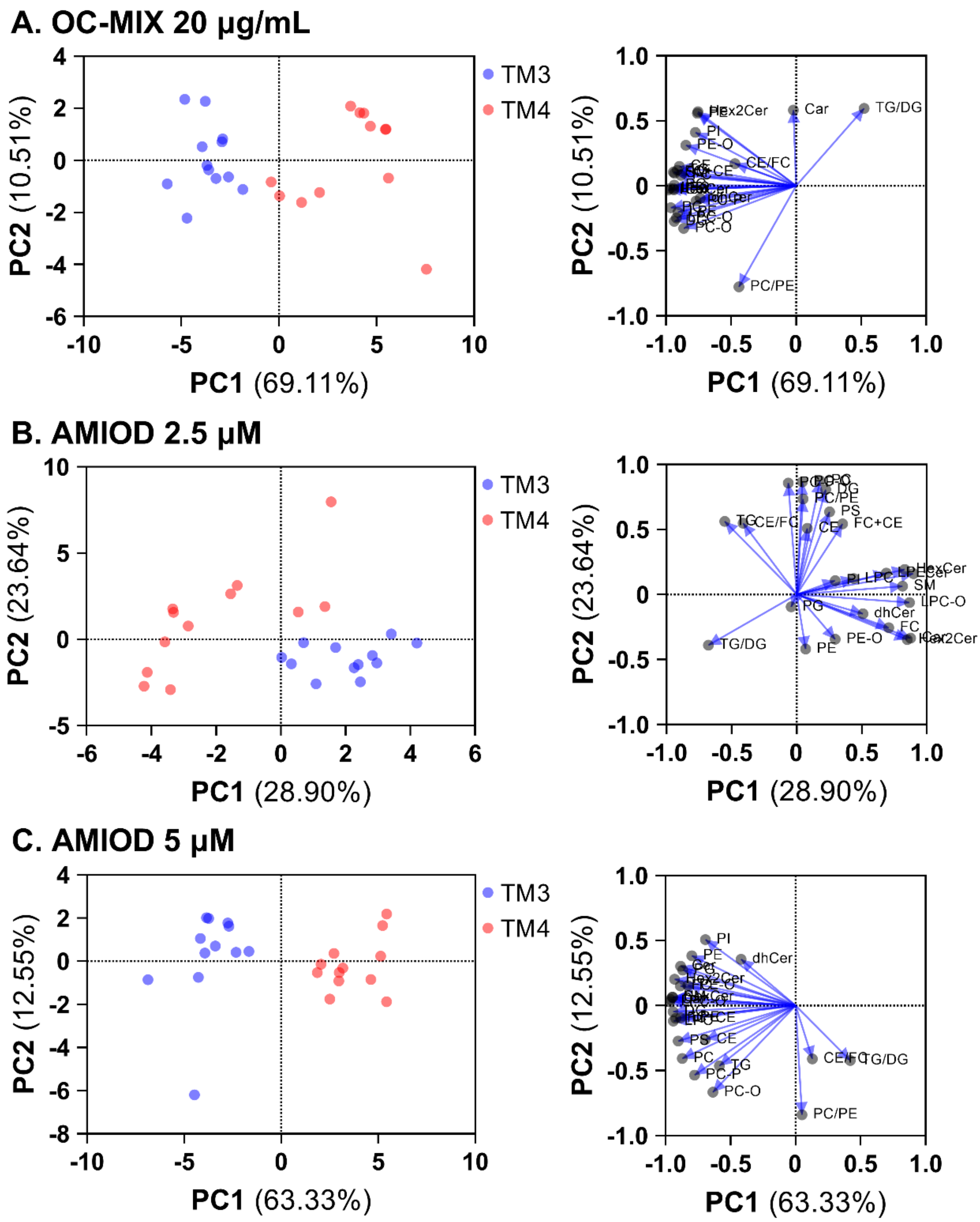

Supplementary Figure S23: Differential lipid-disrupting effects of OC-MIX (A) and amiodarone (B, C) in Sertoli TM4 and Leydig TM3 cells. PCA (principal component analysis) score and loading plots illustrate the lipid profile variations, expressed as Log_2(fold-change) relative to solvent control (SC), across lipid subclasses in treated TM3 and TM4 cells. The experiments were conducted three times independently in quadruplicate (n = 12).

References

Fahy, E., Subramaniam, S., Murphy, R. C., Nishijima, M., Raetz, C. R. H., Shimizu, T., Spener, F., van Meer, G., Wakelam, M. J. O., & Dennis, E. A. (2009). Update of the LIPID MAPS comprehensive classification system for lipids. *Journal of Lipid Research*, *50 Suppl*(Suppl), S9-14. https://doi.org/10.1194/jlr.R800095-JLR200

Gadara, D., Berka, V., & Spacil, Z. (2024). High-Throughput Microbore LC-MS Lipidomics to Investigate APOE Phenotypes. *Analytical Chemistry*, *96*(1), 59–66. https://doi.org/10.1021/acs.analchem.3c02652

Gaud, C., C Sousa, B., Nguyen, A., Fedorova, M., Ni, Z., O'Donnell, V. B., Wakelam, M. J. O., Andrews, S., & Lopez-Clavijo, A. F. (2021). BioPAN: a web-based tool to explore mammalian lipidome metabolic pathways on LIPID MAPS. *F1000Research*, *10*, 4. https://doi.org/10.12688/f1000research.28022.2

Huynh, K., Barlow, C. K., Jayawardana, K. S., Weir, J. M., Mellett, N. A., Cinel, M., Magliano, D. J., Shaw, J. E., Drew, B. G., & Meikle, P. J. (2019). High-Throughput Plasma Lipidomics: Detailed Mapping of the Associations with Cardiometabolic Risk Factors. *Cell Chemical Biology*, *26*(1), 71-84.e4. https://doi.org/https://doi.org/10.1016/j.chembiol.2018.10.008

Kubincová, P., Sychrová, E., Raška, J., Basu, A., Yawer, A., Dydowiczová, A., Babica, P., Sovadinová, I., Kubincova, P., Sychrova, E., Raska, J., Basu, A., Yawer, A., Dydowiczova, A., Babica, P., & Sovadinova, I. (2019). Polycyclic Aromatic Hydrocarbons and Endocrine Disruption: Role of Testicular Gap Junctional Intercellular Communication and Connexins. *Toxicological Sciences : An Official Journal of the Society of Toxicology*, *169*(1), 70–83. https://doi.org/10.1093/toxsci/kfz023

Liebisch, G., Fahy, E., Aoki, J., Dennis, E. A., Durand, T., Ejsing, C. S., Fedorova, M., Feussner, I., Griffiths, W. J., Köfeler, H., Merrill Jr., A. H., Murphy, R. C., O'Donnell, V. B., Oskolkova, O., Subramaniam, S., Wakelam, M. J. O., & Spener, F. (2020). Update on LIPID MAPS classification, nomenclature, and shorthand notation for MS-derived lipid structures. *Journal of Lipid Research*, *61*(12), 1539–1555. https://doi.org/10.1194/jlr.S120001025

Liebisch, G., Vizcaíno, J. A., Köfeler, H., Trötzmüller, M., Griffiths, W. J., Schmitz, G., Spener, F., & Wakelam, M. J. O. (2013). Shorthand notation for lipid structures derived from mass spectrometry. *Journal of Lipid Research*, *54*(6), 1523–1530. https://doi.org/https://doi.org/10.1194/jlr.M033506

Negi, C. K., Gadara, D., Kohoutek, J., Bajard, L., Spáčil, Z., & Blaha, L. (2023). Replacement Flame-Retardant 2-Ethylhexyldiphenyl Phosphate (EHDPP) Disrupts Hepatic Lipidome: Evidence from Human 3D Hepatospheroid Cell Culture. *Environmental Science & Technology*, *57*(5), 2006–2018. https://doi.org/10.1021/acs.est.2c03998

Sychrová, E., Yawer, A., Labohá, P., Basu, A., Dydowiczová, A., Virmani, I., Babica, P., & Sovadinová, I. (2022). In vitro testicular toxicity of environmentally relevant endocrine-disrupting chemicals: 2D vs. 3D models of prepubertal Leydig TM3 cells. *Environmental Toxicology and Pharmacology*, *93*, 103869. https://doi.org/https://doi.org/10.1016/j.etap.2022.103869

Virmani, I., Sychrová, E., Řehůřková, E., Gadara, D., Spáčil, Z., Novák, J., & Sovadinová, I. (2026). Real-life organochlorine mixture-induced lipid dysregulation and oxidative stress in Leydig TM3 cells: Mechanistic insights into male reprotoxicity. *Toxicology*, *519*, 154332. https://doi.org/https://doi.org/10.1016/j.tox.2025.154332

Yawer, A., Sychrová, E., Raška, J., Babica, P., & Sovadinová, I. (2022). Endocrine-disrupting chemicals affect Sertoli TM4 cell functionality through dysregulation of gap junctional intercellular communication in vitro. *Food and Chemical Toxicology : An International Journal Published for the British Industrial Biological Research Association*, *164*, 113004. https://doi.org/10.1016/j.fct.2022.113004
